# Regulation of YAP and Wnt signaling by the endosomal protein MAMDC4

**DOI:** 10.1371/journal.pone.0296003

**Published:** 2024-05-24

**Authors:** Christopher M. Cox, Meng-Han Wu, Marco Padilla-Rodriguez, Isabella Blum, Samina Momtaz, Stefanie A. T. Mitchell, Jean M. Wilson

**Affiliations:** 1 Department of Cellular and Molecular Medicine, University of Arizona, Tucson, AZ, United States of America; 2 The University of Arizona Cancer Center, University of Arizona, Tucson, AZ, United States of America; 3 Bio5 Institute, University of Arizona, Tucson, AZ, United States of America; University of Colorado Boulder, UNITED STATES

## Abstract

Maintenance of the intestinal epithelium requires constant self-renewal and regeneration. Tight regulation of proliferation and differentiation of intestinal stem cells within the crypt region is critical to maintaining homeostasis. The transcriptional co-factors β-catenin and YAP are required for proliferation during normal homeostasis as well as intestinal regeneration after injury: aberrant signaling activity results in over proliferation and tumorigenesis. Although both YAP and β-catenin activity are controlled along canonical pathways, it is becoming increasingly clear that non-canonical regulation of these transcriptional regulators plays a role in fine tuning their activity. We have shown previously that MAMDC4 (Endotubin, AEGP), an integral membrane protein present in endosomes, regulates both YAP and β-catenin activity in kidney epithelial cells and in the developing intestinal epithelium. Here we show that MAMDC4 interacts with members of the signalosome and mediates cross-talk between YAP and β-catenin. Interestingly, this cross-talk occurs through a non-canonical pathway involving interactions between AMOT:YAP and AMOT:β-catenin.

## Introduction

The epithelium that lines the gastrointestinal tract is highly dynamic and turns over every 5–7 days. This high turnover necessitates constant renewal through mitosis in the intestinal crypt and then migration of differentiating cells from the crypt to the villus. The canonical Wnt/β-catenin signaling pathway plays a central role in both intestinal development and stem cell homeostasis and proliferation, and dysregulation of this signaling leads to aberrant development and cancer [1–6]. In addition, other signaling pathways, such as those regulating the co-transcriptional regulator YAP, control proliferation after injury to maintain homeostasis [7, 8].

MAMDC4 (Endotubin, AEGP) is a single pass integral membrane protein that was first identified as a marker of the apical endocytic complex in the developing intestine, but is ubiquitously expressed in epithelial cells [9]. It is highly conserved, with 84% similarity and 74% identity between mouse and human. Furthermore, over-expression of MAMDC4 leads to an increase in proliferation and loss of contact inhibition in MDCK cells [10]. Unlike canonical growth regulation that is mediated by the Hippo kinase cascade, the overgrowth and proliferation phenotype observed with over-expression of MAMDC4 is mediated through regulation of YAP interaction with the junctional protein AMOT [10]. *In vivo*, knockout of MAMDC4 in the developing intestine leads to defects in membrane trafficking and loss of both apical endocytic vesicles and the apical giant lysosome in villus epithelial cells [11]. Importantly, *in vivo* deletion of MAMDC4 in the developing intestine also results in decreased Wnt signaling and epithelial proliferation in the intervillus region/crypt at this stage of development [12].

Wnt signaling is essential for epithelial homeostasis in the intestine, and previous studies have shown that Wnt interacts with YAP to regulate intestinal epithelial proliferation [13–15]. We have identified independent roles for MAMDC4 in YAP and Wnt signaling [10, 11]. In these studies, we asked whether MAMDC4 could modulate cross-talk between YAP and Wnt signaling. Here, we show that deletion of MAMDC4 results in decreased proliferation in vitro and decreased activation of the YAP and Wnt pathway. In contrast, overexpression of MAMDC4 increased YAP expression levels and Wnt signaling, and this increase is mediated by the cytoplasmic domain of MAMDC4. We also find that MAMDC4 interacts with components of the Wnt signalosome and that the association of both YAP and β-catenin with the scaffolding protein AMOT is modulated by the level of MAMDC4 expression. These results support a model where MAMDC4 acts in a non-canonical pathway to regulate both β-catenin and YAP activity independent of the Wnt ligand and Hippo kinase activity.

## Results

**Fig 1:**
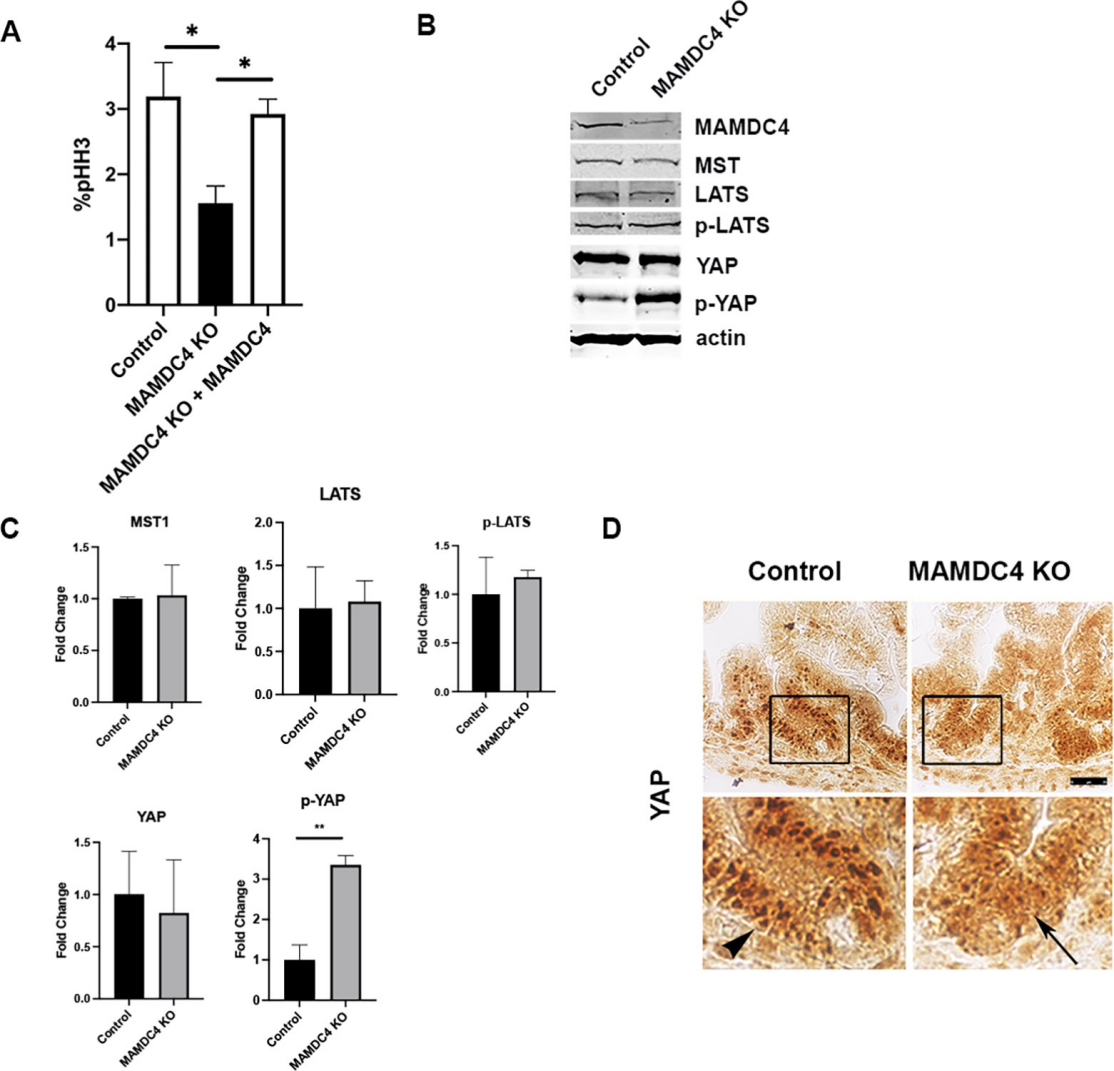
MAMDC4 deletion decreases cell proliferation and YAP nuclear localization. (A) CRISPR/Cas9 Caco2_BBE_ control and MAMDC4 KO cells were grown on coverslips for 48 hours and labeled with antibodies against pHH3 and counterstained with DAPI. pHH3 positive cells were quantified by immunofluorescence. pHH3 labeling is decreased after MAMDC4 KO, and MAMDC4 overexpression rescues the proliferation phenotype. 500 to 1000 cells were counted for each experimental condition (n = 3). (B, C) CRISPR/Cas9 Caco2_BBE_ control and MAMDC4 KO cells were analyzed by Western blot. There is a 3-fold increase in p-YAP in MAMDC4 KO cells and no change in levels of the upstream Hippo kinase pathway activators of YAP. Error bars represent SD. *P<0.05, **P<0.01. Statistical significance was determined by unpaired Student’s t-test (n = 3). Residual MAMDC4 is due to persistence of cells that escaped selection. (D) Postnatal day 3 (P3) ileum from MAMDC4 control and MAMDC4 KO mice were labelled with antibodies against YAP. Rectangle denotes areas of inset below. YAP protein is enriched in the nucleus in control ileum (arrowhead) and nuclear labeling is decreased in MAMDC4 KO ileum (arrow). *Scale bar*: 25μm.

MAMDC4 is an integral membrane protein that localizes to the apical endosomal compartment of epithelial cells [9, 16, 17] and, in a kidney cell line *in vitro*, knockdown of MAMDC4 decreases proliferation whereas overexpression of either the cytoplasmic tail or the full length form of MAMDC4 increases proliferation [10]. We have also shown that, during development of the intestine, knockout of MAMDC4 decreases proliferation [12]. To test if expression of MAMDC4 impacts proliferation in intestinal epithelial cells *in vitro*, we utilized the colon cancer Caco2_BBE_ cell line. MAMDC4 was knocked out (KO) via CRISPR/Cas9 gene editing [10] and proliferation was measured by phospho-Histone H3 (pHH3) labeling of cells grown on coverslips. MAMDC4 KO cells had reduced pHH3 immunolabeling compared to control cells, indicating decreased proliferation (Fig 1A). To control for off-target effects, we transfected the MAMDC4 KO cells with a plasmid expressing MAMDC4. Overexpression of MAMDC4 in the Caco2_BBE_ MAMDC4 KO cells rescues the proliferation defect (Fig 1A and S1 Fig).

YAP activity is coordinated by several pathways that control the phosphorylation state of YAP [18, 19]. The classical Hippo pathway regulates YAP activity by phosphorylation of serine 127 (S127) by LATS kinase, which results in cytoplasmic retention and degradation of YAP [20, 21]. We previously found that YAP mediates the increased proliferation when MAMDC4 is overexpressed in kidney epithelial cells, but not through YAP phosphorylation [10]. To test if MAMDC4 impacts YAP phosphorylation in intestinal epithelial cells, we examined the phosphorylation state of YAP in control and MAMDC4 KO cells using antibodies against YAP-S127. Interestingly, MAMDC4 KO cells have increased phosphorylated YAP-S127 compared to control cells (Fig 1B and 1C). However, this appears not to be due to regulation by Hippo signaling, as there is no change in the levels of upstream kinases or of the phosphorylation state of the upstream kinase LATS (Fig 1B and 1C). These results suggest that MAMDC4 regulates YAP localization and activity independent of Hippo signaling.

To determine if loss of MAMDC4 results in changes in nuclear localization of YAP, we induced MAMDC4 KO at embryonic day 15 (e15) through 4-hydroxytamoxifen (4-OHT) induction of the Cre recombinase under control of the villin promoter in MAMDC4fl/fl, Cre+ mice, with MAMDC4fl/fl, Cre- mice as controls [22]. Ileum was collected at postnatal day 3 and labelled with antibodies against YAP. In control intestinal epithelium, YAP is localized to the nucleus in cells of the intervillous region of the ileum (Fig 1D; arrowhead). However, MAMDC4 KO results in a loss of nuclear localization of YAP in these cells. (Fig 1D; arrow), suggesting that MAMDC4 regulates localization of YAP in the proliferative zone in the developing intestine.

**Fig 2:**
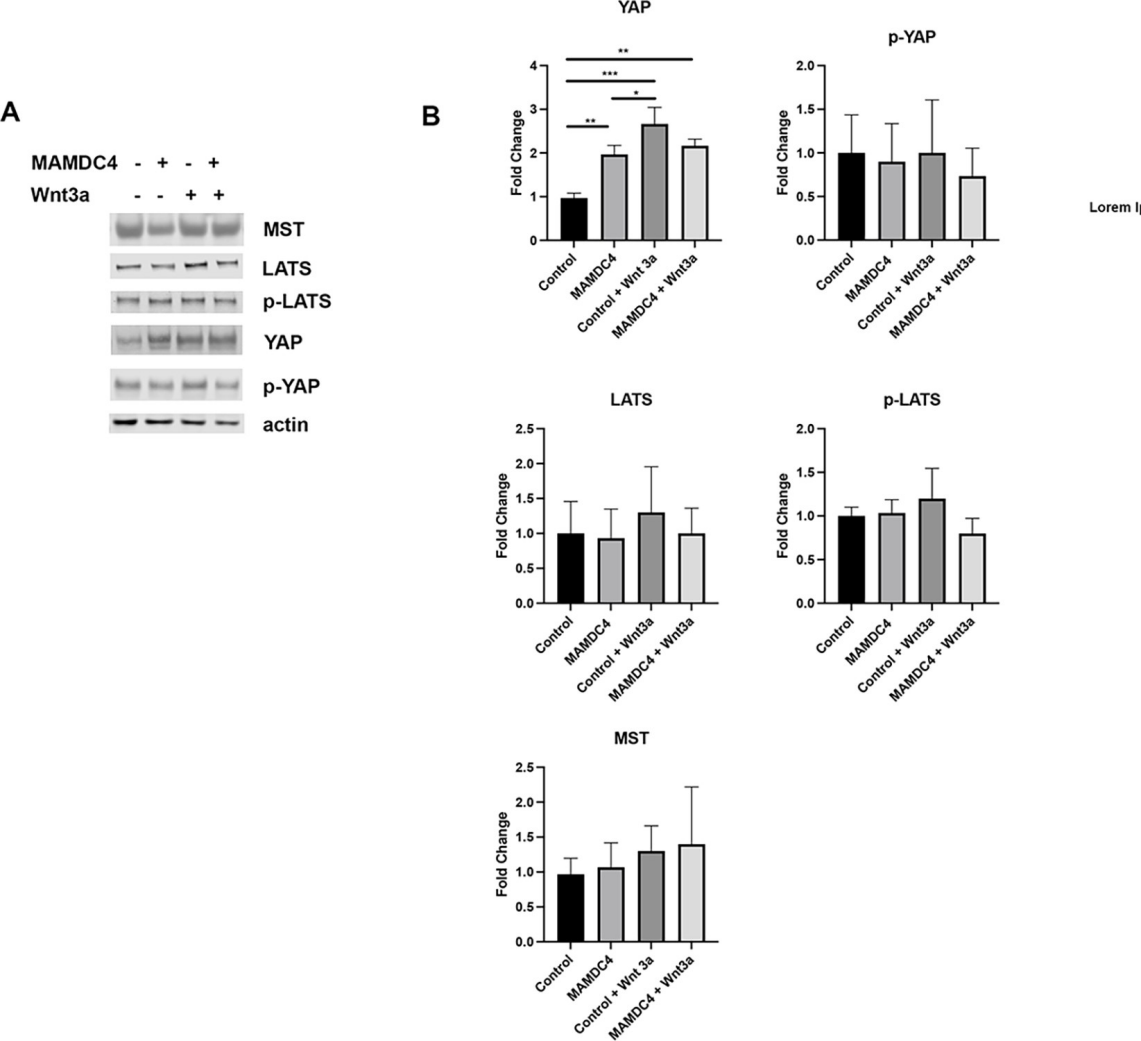
MAMDC4 overexpression increases YAP protein expression. (A) Western blot analysis of HEK293 cells in the presence and absence of Wnt and with or without MAMDC4 overexpression. (B) Quantification of A shows that MAMDC4 overexpression and Wnt3a treatment results in increased YAP. There is no change in phosphorylation of the upstream Hippo kinase LATS or expression levels of LATS or MST. Error bars represent SD. **P<0.05, **P<0.01, ***P<0.001. Statistical significance was determined using an Ordinary one-way ANOVA with Tukey’s Multiple Comparisons (n = 3).

In addition to our findings that MAMDC4 impacts YAP signaling, we have shown that knockout of MAMDC4 impacts Wnt signaling in the developing intestine as measured by TCF/LEF-GFP expression in enteroids and active β-catenin levels in intestinal epithelium [12]. Importantly, in the intestine, YAP and Wnt/β-catenin signaling pathways can reciprocally regulate the activity of their counterpart through multiple mechanisms [23]. To test the role that MAMDC4 plays in regulation of YAP in the context of normal Wnt signaling, we overexpressed MAMDC4 in HEK293 cells, which have a functional Wnt signaling pathway and have been used extensively to define Wnt signaling [24–30]. Similar to our previous results using MDCK cells, overexpression of MAMDC4 increases YAP protein levels independent of Hippo signaling (Fig 2A and 2B) [10]. Unlike with MAMDC4 knockout, YAP-S127 levels do not change with MAMDC4 overexpression, further supporting the finding that MAMDC4 does not regulate Hippo kinase activity in intestinal epithelial cells (Fig 2A and 2B).

**Fig 3:**
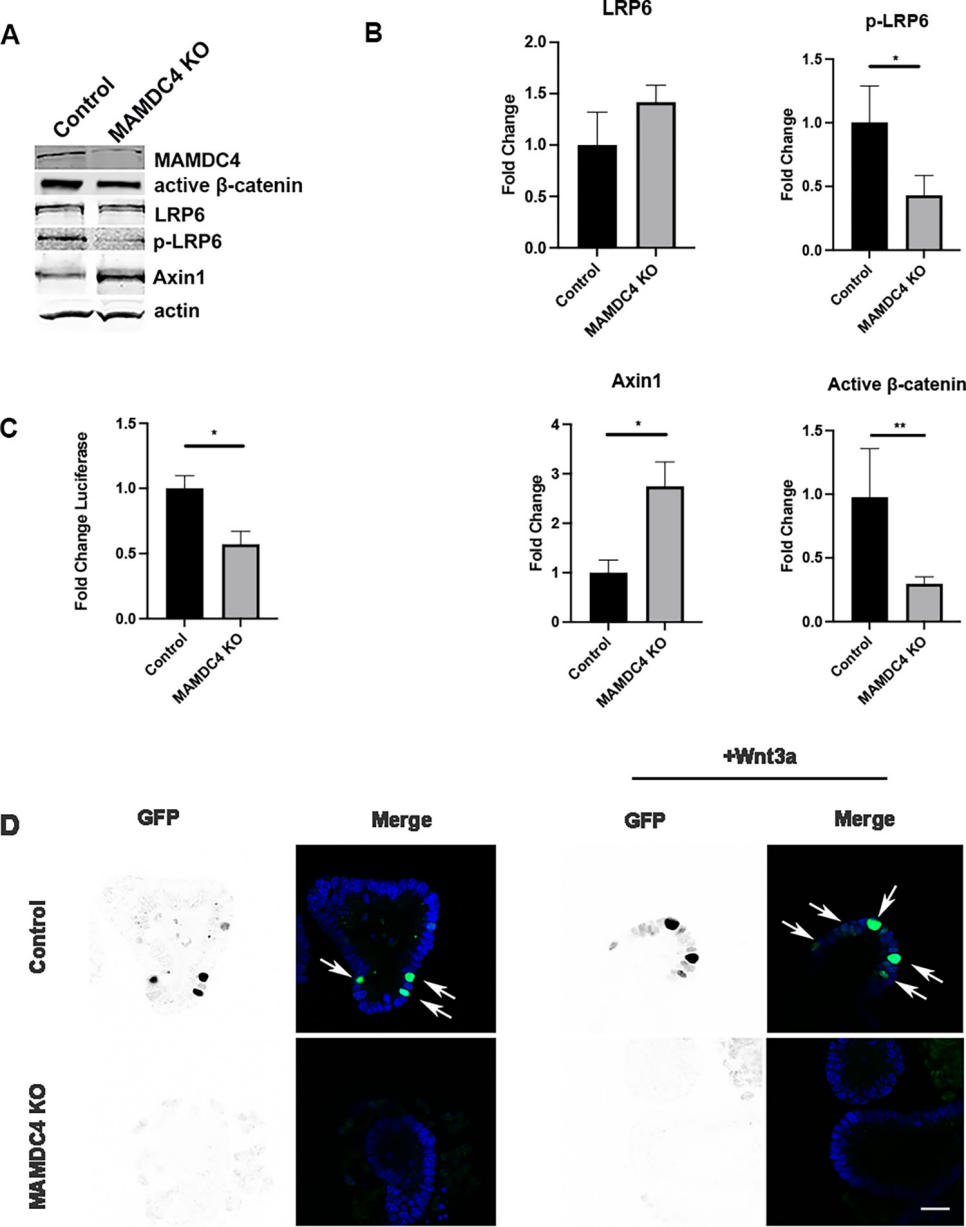
MAMDC4 deletion inhibits Wnt signaling. (A) CRISPR/Cas9 Caco2BBE control and MAMDC4 KO cells were analyzed by Western blot. (B) Quantification of Western blots in A. There is a decrease in non-phospho (active) b-catenin and p-LRP6 (Ser 1490) and an increase in Axin1 in MAMDC4 KO cells. Residual MAMDC4 is due to persistence of cells that escaped selection (n = 3). (C) CRISPR/Cas9 Caco2_BBE_ control and MAMDC4 KO cells were transfected with Super TOPFlash TCF/LEF luciferase reporter and renilla control plasmid and grown for 48 hours. Wnt reporter activity is decreased in MAMDC4 KO cells (n = 5). (D) Enteroids were derived from postnatal day 11 mice, grown in Matrigel and treated for 6hrs with Wnt3a, fixed, and counterstained with DAPI. TCF/LEF:H2B -GFP expression (arrows) was visualized by confocal microscopy. Nuclear TCF/LEF:H2B -GFP expression increases with Wnt stimulation in control enteroids (arrows), There is no TCF/LEF:H2B -GFP expression in MAMDC4 KO enteroids in nuclei in control or Wnt stimulation conditions, indicateing a loss of β-catenin localization to the nucleus in MAMDC4 KO enteroids. Error bars represent SD. *P<0.05, ***P<0.001. Statistical significance was determined by unpaired Student’s t-test *Scale bar*: 20μm.

Our findings that MAMDC4 impacts both YAP expression and localization and Wnt signaling in the developing intestine [12] (Fig 1D) led us to probe in more detail the effects of MAMDC4 on Wnt signaling. To test the effects of MAMDC4 expression on Wnt pathway components, we performed immunoblot analysis of control and MAMDC4 KO Caco2_BBE_ cells. As in the developing intestine [12], this analysis shows decreased phospho-LRP6 (p-LRP6), an indicator of Wnt receptor activation, increased Axin1 levels, and decreased active β-catenin after MAMDC4 knockout (Fig 3A and 3B). Combined, these results suggest that Wnt activity is impaired with loss of MAMDC4. To further test for β-catenin activity in these cells, we performed luciferase assays using the M50 Super TOPFlash TCF/LEF reporter [31]. For these experiments, MAMDC4 KO Caco2_BBE_ and control cells were transfected with the reporter constructs and a renilla plasmid to provide a control for transfection efficiency. As shown in Fig 3C, MAMDC4 knockout results in decreased luciferase signal compared to control cells. To further examine β-catenin localization we derived enteroids from MAMDC4^fl/fl^ Cre−/TCF/LEF:H2B -GFP+ and MAMDC4^fl/fl^ Cre+/TCF/LEF:H2B-GFP+ as described previously [12]. TCF/LEF:H2B-GFP expression is localized to the nucleus in control enteroids, and this expression is increased with Wnt3a treatment. However, there is a no nuclear GFP expression in the MAMDC4 KO enteroids which is unchanged with Wnt3a treatment (Fig 3D).

Together, these results indicate that MAMDC4 mediates both YAP and β-catenin nuclear localization and signaling.

**Fig 4:**
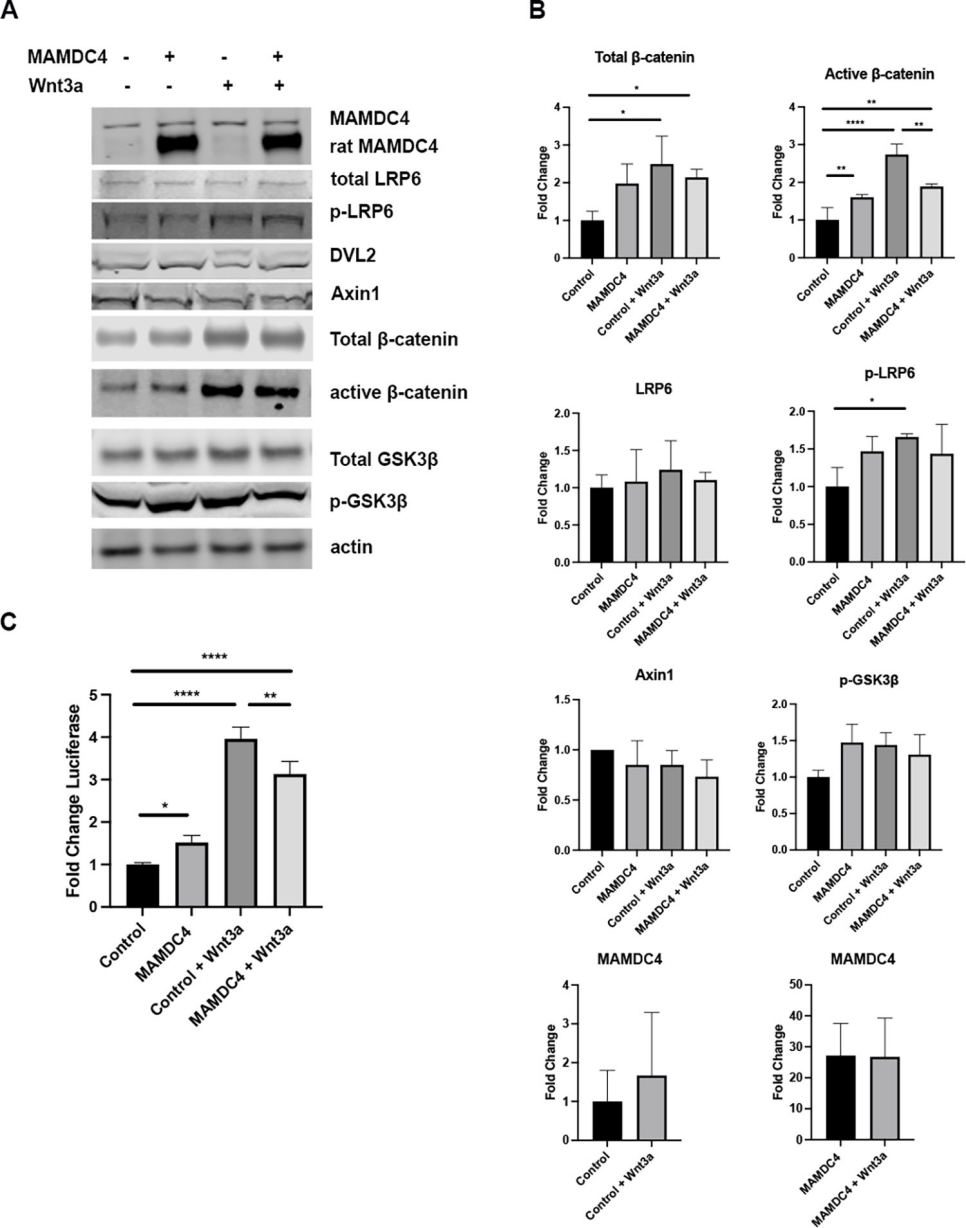
MAMDC4 promotes Wnt/β-catenin signaling. (A) Western blot analysis of HEK293 cells in the presence and absence of Wnt and with or without MAMDC4 overexpression. (B) Quantification of A shows that MAMDC4 overexpression and Wnt3a treatment results in increased non-phospho (active) β-catenin and p-LRP6. There is decreased active β-catenin in Wnt3a stimulated cells over-expressing MAMDC4 relative to Wnt3a treated vector control cells. The are no changes in total LRP6 or Axin1 levels with MAMDC4 overexpression or Wnt stimulation. Both endogenous and exogenous MAMDC4 levels are unchanged with Wnt stimulation (n = 3). (C) HEK293 cells expressing MAMDC4 or vector control were transfected with Super TOPFlash TCF/LEF reporter and renilla control vector. Cells were stimulated with Wnt3a conditioned media for 6 hours. There is an increase in luciferase with MAMDC4 overexpression. In control transfected cells, Wnt3a stimulates luciferase activity 4-fold, however when MAMDC4 is overexpressed, this increase is dampened. Error bars represent SD. *P<0.05, **P<0.01, ***P<0.001, ****P<0.0001. Statistical significance was determined using an Ordinary one-way ANOVA with Tukey’s Multiple Comparisons. (n = 3).

As described above, overexpression of MAMDC4 results in increased levels of YAP protein and increased Wnt signaling. Conversely, MAMDC4 knockout decreases YAP and Wnt signaling. To further define the mechanisms by which MAMDC4 could impact these pathways, we transfected HEK293 cells with full length MAMDC4 [17] and stimulated with Wnt3a for 6 hours, followed by immunoblotting for components of the Wnt signaling pathway. Wnt treatment of HEK293 cells results in an increase in total β-catenin and p-LRP6, as expected. As shown in Fig 4A and 4B, there is increased active β-catenin with MAMDC4 overexpression that is increased further after Wnt3a treatment. Notably, MAMDC4 overexpressing cells treated with Wnt3a show decreased levels of active β-catenin relative to control cells treated with Wnt3a (Fig 4). These results suggest that MAMDC4 has a dampening effect on ligand-induced Wnt signaling. However, total LRP6 and MAMDC4 levels do not change with Wnt stimulation, suggesting that Wnt is not targeting these factors to the lysosome. Interestingly, while MAMDC4 KO in Caco2_BBE_ cells (which contain mutations in the Wnt pathway) have increased Axin1 and decreased p-LRP6 levels (Fig 3A and 3B), there is no change in Axin1 or p-LRP6 when MAMDC4 is overexpressed in HEK293 cells (Fig 4A). In addition, there is no change in DVL2 or GSK3β in MAMDC4 overexpressing cells while there is a trend toward increased p-GSK3β in MAMDC4 overexpressing cells (Fig 4A). These results suggest that either MAMDC4 is a limiting component or that MAMDC4 is acting through a non-canonical pathway.

In addition to changes in Wnt signaling pathway components, assay of β-catenin activation using the Super TOPFlash luciferase assay showed that overexpression of MAMDC4 resulted in increased reporter activity (Fig 4C). Again, as expected, addition of Wnt3a increased luciferase activity substantially in control cells. However, while Wnt stimulation of cells overexpressing MAMDC4 resulted in increased luciferase activity, the response was also dampened compared to Wnt stimulation alone, similar to what was observed with levels of active β-catenin (Fig 4A and 4B).

**Fig 5:**
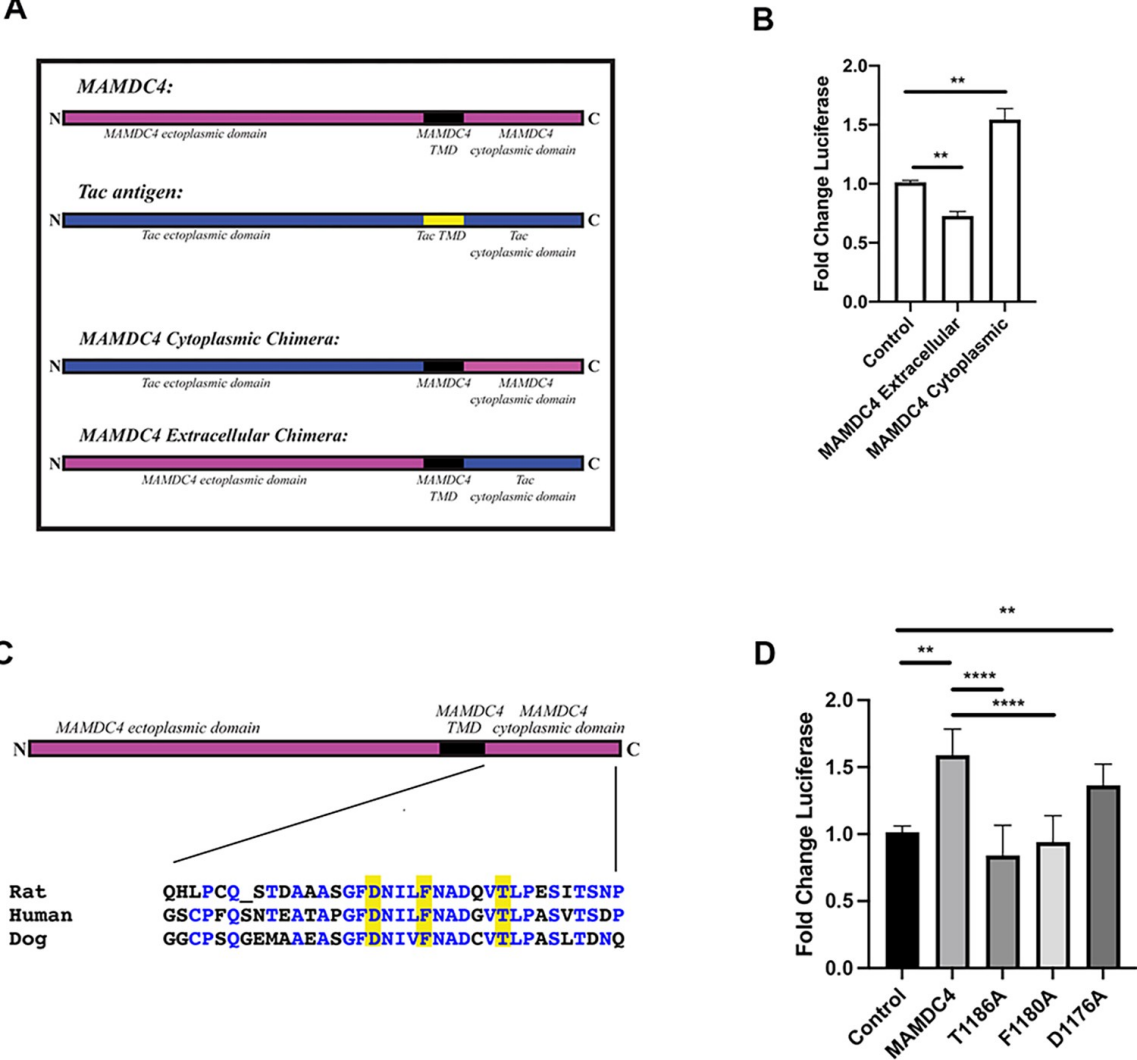
Increased Wnt signaling is mediated by the MAMDC4 cytoplasmic domain. (A) Schematic of MAMDC4/Tac cytoplasmic and extracellular chimeras. (B) HEK293 cells expressing vector control, or a chimera containing the MAMDC4 extra-cellular domain or MAMDC4 cytoplasmic domain were transfected with Super TOPFlash TCF/LEF reporter and renilla control vector. There is an increase in reporter activity with the MAMDC4 cytoplasmic domain (n = 4). (C) Schematic of MAMDC4 point mutation constructs and alignment of cytoplasmic domain amino acid sequences. (D) HEK293 cells expressing MAMDC4 constructs with point mutations in the cytoplasmic tail were transfected with Super TOPFlash TCF/LEF reporter and renilla control vector. The T1186A and F1180A mutations show decreased luciferase activity. n = 3 for T1186A, n = 4 for F1180A and D1176A. **P<0.001, ****P<0.0001. Statistical significance was determined using an Ordinary one-way ANOVA with Tukey’s Multiple Comparisons.

MAMDC4 is a single pass transmembrane protein with a large extracellular domain and a 40 amino acid cytoplasmic domain [17]. We have shown previously that the overexpression of the cytoplasmic domain of MAMDC4 in cells in culture increases YAP signaling and promotes proliferation [10]. To test if the cytoplasmic tail also impacts Wnt signaling, we transfected cells with chimeric constructs containing either the transmembrane and cytoplasmic domain of MAMDC4 with the Tac receptor comprising the opposite domain (Fig 5A) [32]. These constructs have been used previously to define the targeting of MAMDC4 in kidney epithelial cells in culture [32]. As shown in Fig 5B, expression of the chimera containing the MAMDC4 cytoplasmic domain resulted in increased Wnt reporter activity compared to the empty vector control. In contrast, cells expressing the extracellular domain had decreased reporter activity. The increased reporter activity observed with the cytoplasmic domain chimera was similar to that seen with full-length MAMDC4 (Fig 4C). These results show that increased Wnt signaling observed with full-length MAMDC4 is mediated through the cytoplasmic domain and suggest that the extracellular domain may modulate interaction with Wnt ligand or prevent the FZD/LRP6 interaction.

To further define the amino acids in the cytoplasmic domain of MAMDC4 that are responsible for increased Wnt activity, we assayed luciferase activity in cells expressing MAMDC4 constructs that have been engineered to express point mutations that are known to regulate the localization of MAMDC4 in epithelial cells [33]. Importantly, these amino acids are conserved in mammalian MAMDC4, including in humans (Fig 5C). In epithelial cells, mutation of phenylalanine 1180 or threonine 1186 (rat sequence) to alanine (F1180A or T1186A) results in mis-localization of MAMDC4 from the apical endosomes [33]. In contrast, the D1176A point mutation is targeted correctly [33]. To determine if the point mutations also play a role in regulation of Wnt/β-catenin activity we overexpressed the MAMDC4 point mutations in HEK293 cells (S2 Fig). Measurement of Wnt reporter activity in cells expressing these constructs shows decreased reporter activity with expression of either the F1180A and T1186A mutants, but not with expression of D1176A mutation (Fig 5D). These results suggest that either apical endosomal localization of MAMDC4 is required for modulation of Wnt/β-catenin activity or that these amino acids are necessary for incorporation of MAMDC4 into the signalosome.

**Fig 6:**
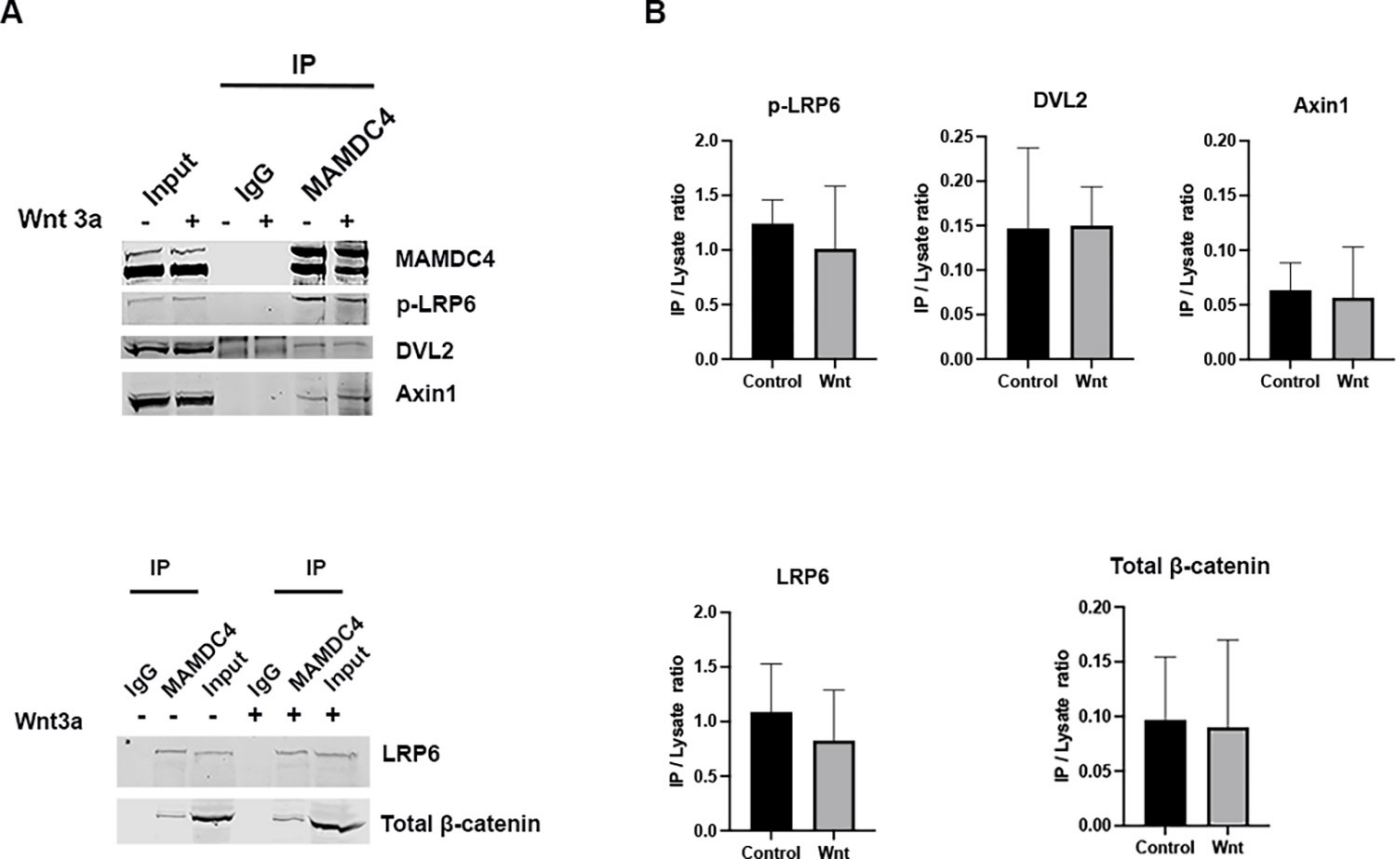
MAMDC4 associates with the Wnt signalosome. (A) HEK293 cells over-expressing MAMDC4 were stimulated with control or Wnt3a conditioned media. Lysates were immunoprecipitated with antibodies against MAMDC4 or control IgG. MAMDC4 associates with LRP6, p-LRP6, total β-catenin, DVL2 and Axin in control and Wnt3a stimulated conditions. There is a substantial amount of LRP6 and p-LRP6 (relative to input) associated with MAMDC4 in both Wnt stimulated and unstimulated conditions. (B) Quantification of signal from immunoprecipitation relative to input. LRP6 and p-LRP6 have a ratio of approximately 1 while the ratio of β-catenin and Axin1 have a ratio of IP to input that is <1/10^th^ of the amount of LRP6 and p-LRP6 immunoprecipitated. All immunoprecipitations were repeated 3 to 5 times and the input lanes represents 1/50^th^ of lysate used for the immunoprecipitation.

To determine if MAMDC4 associates with components of the signalosome, MAMDC4 was overexpressed and immunoprecipitated, followed by blotting for p-LRP6 (Ser 1490), LRP6, Axin1, DVL2, and total β-catenin. MAMDC4 co-immunoprecipitated with all of these signalosome components (Fig 6). However, although MAMDC4 immunoprecipitated multiple signalosome components, only LRP6 and p-LRP6 were substantially enriched with MAMDC4 immunoprecipitation (Fig 6). Importantly, immunoprecipitation following Wnt3a stimulation co-immunoprecipitated similar amounts of LRP6 and p-LRP6 (Fig 6), indicating that the MAMDC4 interaction with the signalosome is ligand-independent.

Canonical Wnt signaling involves ligand binding to the receptor complex, aggregation of the receptor complex which is dependent on localization of DVL2 to the cytosolic region of the receptor [34–37], while Axin1, an integral member of the destruction complex, is recruited to the membrane following Wnt stimulation [38–40]. To test if MAMDC4 influences the localization of the signalosome, we overexpressed DVL2 in MDCK cells expressing control or shRNA directed to MAMDC4. While DVL2 form puncta in 34% of control cells, only 19% of MAMDC4 KD cells show DVL2 puncta (S3 Fig). This data suggests that MAMDC4 regulates signalosome formation.

**Fig 7:**
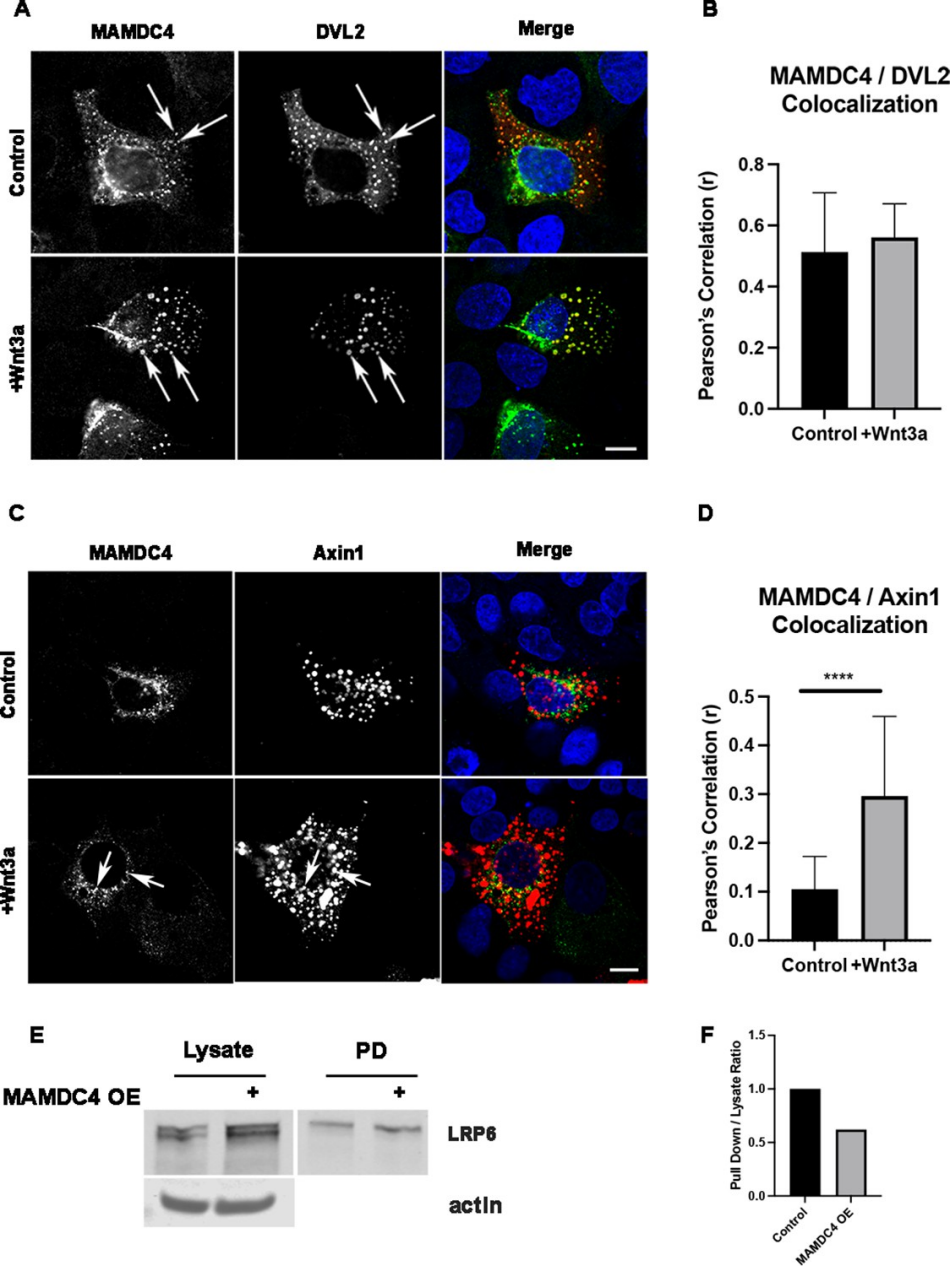
MAMDC4 is colocalized with the WNT signalosome. (A) SKCO cells were transfected with MAMDC4 and DVL2. 48 hours post transfection cultures were treated with vehicle or Wnt3a for 1hr. Cells were labeled with antibodies to MAMDC4 (green) and DVL2 (red) and counterstained with DAPI. MAMDC4 and DVL2 are colocalized in puncta regardless of Wnt3a stimulation *Scale bar*: 10μm. (B) Quantification of Pearson’s Correlation Coefficient (r). Wnt3a treatment does not influence MAMDC4 and DVL2 colocalization. (C) SKCO cells were transfected with MAMDC4 and Axin1. 48 hours post transfection cultures were treated with vehicle or Wnt3a for 5min. Cells were labeled with antibodies to MAMDC4 (green) and Axin1 (red) and counterstained with DAPI. MAMDC4 and Axin1 are colocalized in some puncta after Wnt3a stimulation (arrows). (D) Quantification of Pearson’s Correlation Coefficient (r). There is an increase in MAMDC4 and Axin1 colocalization that is dependent on Wnt3a stimulation. (E) Immunoblot of NeutrAvidin Pull down. (F) Quantification of LRP6 Pull down / Lysate ratio.

Wnt stimulation induces the aggregation of LRP6 at the plasma membrane, and this aggregation is dependent on DVL2 function [34–37]. To assess the level of colocalization of MAMDC4 with members of the signalosome we utilized the intestinal cell line SKCO. MAMDC4 and DVL2 were overexpressed in SKCO cells and 48hrs after transfection cells were treated with vehicle or Wnt3a for 1 hour, fixed and labeled with antibodies to MAMDC4 and DVL2. Confocal imaging shows colocalization of MAMDC4 and DVL2 puncta under both control and Wnt3a stimulation (Fig 7A). There is no change in the Pearson’s correlation coefficient of MAMDC4:DVL2 following Wnt treatment (Fig 7B). Axin is recruited to the membrane 5 min post Wnt stimulation [40]. To test the association of MAMDC4 with Axin1 we overexpressed MAMDC4 and Axin1 in SKCO cells. MAMDC4 and Axin1 show minimal colocalization prior to Wnt stimulation. However, 5min after Wnt3a stimulation, there is a significant increase in colocalization (Fig 7C and 7D). These results suggest that localization of MAMDC4 with DVL2 is independent of Wnt stimulation and the interaction of MAMDC4 with Axin1 requires Wnt3a stimulation. As Axin1 is expressed at high levels and is primarily associated with the destruction complex, these results suggest the Axin1 recruitment to complexes containing MAMDC4 requires Wnt stimulation.

Wnt signaling requires binding of ligand to the receptor complex and endocytosis of the receptor/signalosome which requires both membrane-associated proteins like clathrin, caveolin, and dynamin, in addition to early endosome proteins such as RAB5 [40–44]. MAMDC4 is an endosomal protein and when downregulated in developing intestinal epithelium leads to a loss of the apical endocytic complex and decreased endocytosis in Caco2_BBE_ cells [11]. To test the role of MAMDC4 in Wnt receptor trafficking, we overexpressed MAMDC4 in HEK293 cells, followed by biotinylating proteins on the cell surface. NeutrAvidin pull-down was followed by immunoblotting for LRP6 resulted in a 38% decrease in the amount of cell surface LRP6 when MAMDC4 is overexpressed (Fig 7E and 7F). These results are consistent with increased Wnt signaling with MAMDC4 overexpression.

**Fig 8:**
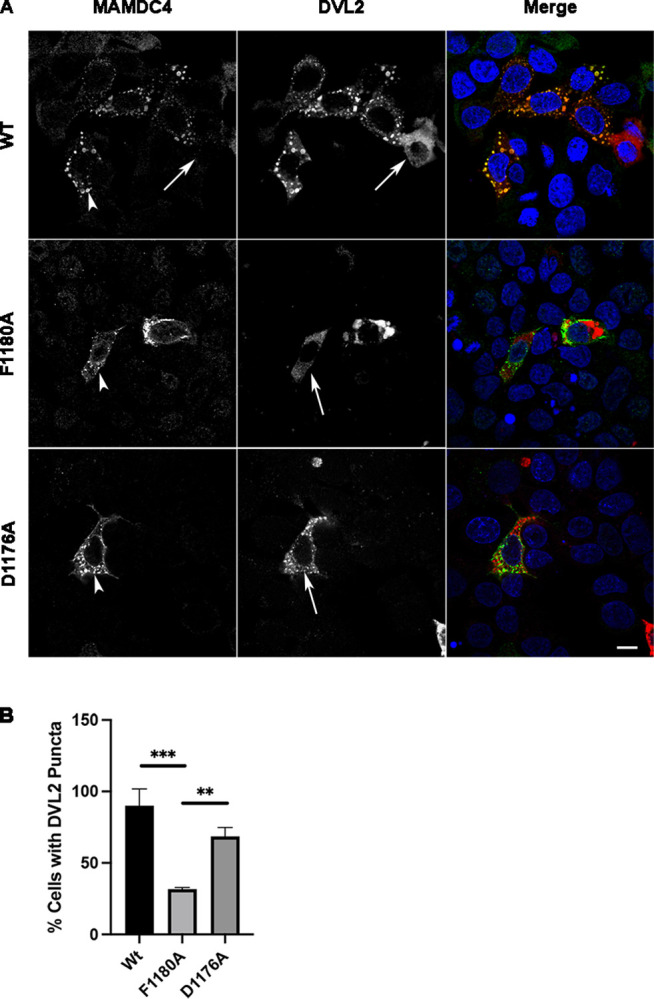
MAMDC4 motifs regulate signalosome localization. SKCO cells were co-transfected with either MAMDC4 or MAMDC4 mutants and DVL2. 48 hours post transfection cells were labeled with antibodies to MAMDC4 (green) and DVL2 (red) and counterstained with DAPI. (A) MAMDC4 and DVL2 are colocalized in puncta, however, DVL2 has diffuse labeling in cells lacking MAMDC4 overexpression (arrow) (B) Quantification of number of cells expressing both MAMDC4 and DVL2, which contain DVL2 puncta. 35 to 45 cells were assessed for each condition, n = 3. **P<0.001, ***P<0.001. Statistical significance was determined using an Ordinary one-way ANOVA. *Scale bar*: 10μm.

Previously, we have shown that MAMDC4 is involved in endocytosis in intestinal epithelial cells and formation of the apical endocytic complex in the developing intestine [11]. More importantly, we have shown that the cytoplasmic region plays a critical role in localization of MAMDC4 and specific, conserved residues within the cytoplasmic region control targeting of MAMDC4 to specialized endosomes [32, 33]. In this study we show the motifs that control targeting of MAMDC4 also influence Wnt/β-catenin signaling. To test if these motifs influence the Wnt signalosome, we overexpressed the MAMDC4 mutants and DVL2 in SKCO cells. Confocal imaging reveals a decrease in DVL2 puncta without MAMDC4 overexpression (Fig 8A, top panels, arrows). Additionally, the F1180A mutation, which fails to induce Wnt/β-catenin signaling, shows reduced DVL2 puncta formation (Fig 8A, middle panels, arrow), while the D1176A mutation which induces β-catenin activity shows an increase in DVL2 puncta (Fig 8B). These data show that correct trafficking of MAMDC4 regulates signalosome organization.

**Fig 9:**
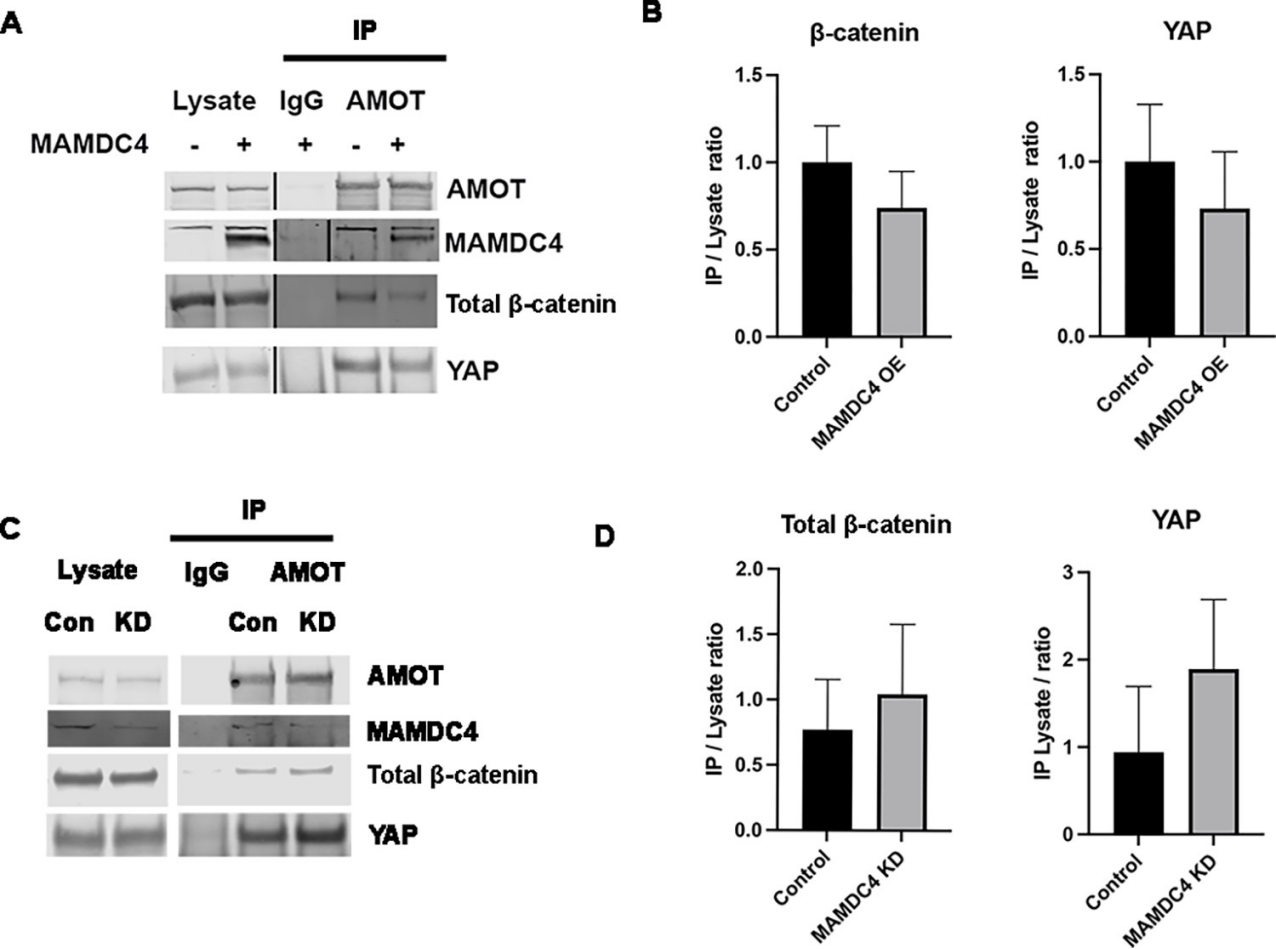
MAMDC4 overexpression disrupts the YAP:AMOT and β-catenin:AMOT interaction. (A) HEK293 cells were transfected with MAMDC4 or empty vector control. Lysates were immunoprecipitated with antibodies to AMOT and examined by immunoblot. The amount of β-catenin and YAP immunoprecipitated with AMOT is reduced when MAMDC4 is overexpressed. Immunoprecipitation was repeated 3 times and a representative image is shown. A single lane, on all immunoblots was eliminated and this is indicated by a vertical line separating the lysate and IgG lanes. Lysate lanes for both MAMDC4 and β-catenin were scanned at lower intensity to avoid over exposure. (B) Quantification of IP/lysate ratios show a decrease in β-catenin and YAP association with AMOT in MAMDC4 overexpressing cells. (C) Lysates were collected from HEK293 cells expressing control or shRNA targeting MAMDC4. Lysates were immunoprecipitated with antibodies to AMOT and examined by immunoblot with antibodies to AMOT, total β-catenin, MAMDC4, and YAP. (D) Quantification of IP/lysate ratio show increase of β-catenin and YAP with AMOT in MAMDC4 KD cells.

The membrane associated proteins AMOT and AMOTL2 have known binding domains for YAP and β-catenin, co-immunoprecipitate with signalosome components, and have been shown to regulate these signaling pathways [13, 45–50]. To test if MAMDC4 regulates the association of β-catenin and YAP with AMOT, we overexpressed MAMDC4 in HEK293 cells. Lysates were immunoprecipitated with antibodies to AMOT followed by immunoblotting for β-catenin and YAP. Both β-catenin and YAP co-immunoprecipitated with AMOT and, in the presence of MAMDC4 overexpression, there is a reduction in the amount of β-catenin and YAP co-immunoprecipitated (Fig 9). To further test this model, we generated control and MAMDC4 KD HEK293 cells (S4 Fig). With MAMDC4 KD, there is an increase in the amount of β-catenin and YAP co-immunoprecipitated with AMOT. These results suggest that MAMDC4 competes with β-catenin and YAP for binding to AMOT, thus regulating the amount of β-catenin and YAP sequestered in the cytoplasm.

## Discussion

Membrane trafficking modulates an array of signaling pathways [51–53]. In the Wnt pathway, endocytosis promotes activation of the β-catenin, and maturation of the endosomes leads to the degradation of the complex [54–56]. In addition, specialized and recycling endosomes regulate cytoplasmic localization of β-catenin and YAP [10, 42, 46, 49, 57, 58]. Here, we report that the endosomal protein MAMDC4 regulates YAP and Wnt signaling to control epithelial proliferation.

Multiple signaling pathways are essential for the development, homeostasis and regeneration of the intestinal epithelium [59–62]. The canonical Wnt/β-catenin pathway is the primary proliferative signal in the developing intestine and also plays a central role in stem cell homeostasis; dysregulation leads to aberrant development and cancer [1, 2, 5, 63–66]. While the major players that mediate canonical Wnt signaling have been known for some time, it is now known that additional membrane proteins can enhance or suppress Wnt signaling [67, 68]. Multiple integral membrane proteins, or TMEM’s, impact β-catenin by promoting degradation of the destruction complex (TMEM9) or inhibit β-catenin signaling by sequestering the destruction complex, along with β-catenin in MVB’s (TMEM88) [69]. MAMDC4 is a Type 1 integral membrane protein with a short cytoplasmic domain [16, 17]. Like TMEM59 [70], which associates with the signalosome to promote β-catenin signaling, we find that MAMDC4 interacts with components of the signalosome. Further, overexpression MAMDC4 in the full length form as well as the cytoplasmic domain increases β-catenin activity. Interestingly, mutations in MAMDC4 that regulate the endosomal localization of MAMDC4 disrupt DVL2 puncta formation. These data suggest that the cytoplasmic domain is mediating the interaction with signalosome components.

YAP is a regulator of intestinal stem cell function, and is important for regeneration of the epithelium after damage [71–73]. Furthermore, it is upregulated in intestinal cancers [74, 75]. We have shown that MAMDC4 is localized to stem cells of the intestinal epithelium and loss of MAMDC4 results in a decrease in LGR5+ stem cells [12]. Our finding that MAMDC4 regulates the nuclear localization of YAP in in the proliferative zone of the intestinal epithelium is consistent with our previous results in a kidney cell line where overexpression of MAMDC4 results in localization of YAP to the nucleus [10]. Nuclear localization of YAP is controlled by other integral membrane proteins, including EGFR [76, 77]. The phosphorylation and downregulation of YAP without canonical stimulation shows that MAMDC4 interaction with YAP is independent of Hippo activity. Multiple non-canonical kinases and phosphatases including aPKC, Akt, PP2a, PPM1A and PP1 are known to impact the stability of β-catenin and YAP in intestinal stem cells and multiple cancer cell types [19, 78–81]. In addition to non-canonical regulation of the phosphorylation state of β-catenin and YAP, the membrane proteins AMOT and AMOTL2 have been shown to regulate the activity of both YAP and β-catenin independent of the canonical activation of the Wnt and Hippo pathways [45, 47, 82, 83].

Our findings that MAMDC4 impacts both β-catenin and YAP signaling opens up the possibility that MAMDC4 functions to regulate β-catenin/YAP crosstalk. Crosstalk between these pathways occurs by multiple mechanisms, such as by incorporation of YAP to the β-catenin destruction complex where Wnt stimulation drives YAP nuclear localization independent of the Hippo pathway [13]. Conversely, Hippo pathway activation, which leads to phosphorylation of YAP, induces cytoplasmic sequestration of both β-catenin and DVL2 [7, 84]. Furthermore, β-catenin activity is required for YAP-induced organ overgrowth, and YAP promotes β-catenin signaling, enterocyte renewal and regeneration after injury [85, 86].

We have shown previously that MAMDC4 interacts with the N-terminal region of the junctional protein AMOT, which has also been implicated in regulation of YAP and β-catenin activity [10, 45, 46, 48]. Importantly, it is the same N-terminal region of AMOT and AMOTL2 that is required for binding of MAMDC4, YAP, and β-catenin. Furthermore, AMOT is expressed in undifferentiated cells of the intestinal epithelium where β-catenin and YAP signaling are active (https://www.proteinatlas.org). MAMDC4 colocalizes with AMOT and AMOTL2 on endosomal membranes [10] and regulates β-catenin signaling and YAP nuclear localization in the intestinal epithelium [12, 57]. The signalosome protein, Axin1, interacts with AMOT, and this interaction is dependent on YAP [13]. Here, we show that YAP and β-catenin interaction with AMOT is regulated by MAMDC4. Together, this supports a model where non-canonical regulation of YAP and β-catenin occurs in an endosomal compartment where MAMDC4 promotes YAP and β-catenin signaling through competition for binding with AMOT.

## Experimental procedures

### Mice

The EDTB (MAMDC4) and Villin-CreERT2 mouse colonies were previously described [11]. MAMDC4fl/+/ Villin-CreERT2 males were mated with MAMDC4fl/fl females. Pregnant females were gavaged with 4-hydroxytamoxifen (4-OHT; cat. T176-50; Sigma) on day 15 of gestation (E15), and offspring were collected at postnatal day 3 (P3) as described [12].

TCF/LEF:H2BGFP (013752) mice were crossed with the MAMDC4fl/fl / Villin-CreERT2 to generate MAMDC4fl/fl / TCF/LEF:H2BGFP females and MAMDC4fl/fl / Villin-CreRTt2 / TCF/LEF:H2BGFP males. Dams were gavaged with 4-OHT for 5 days starting at postnatal day 5 and enteroids were generated from postnatal day 11 pups as described [12].

All genotyping was carried out by the University of Arizona Genetics Core. The care, maintenance, and treatment of mice used in these studies was approved by the University of Arizona Institutional Animal Care and Use Committee.

### Enteroids

Details for isolation of intestinal ileum, generation and growth of enteroids has been described in detail [12]. MAMDC4 fl/fl Cre- / TCF/LEF:H2BGFP+ and MAMDC4fl/fl Cre+/ TCF/LEF:H2BGFP+ enteroids were treated with 100ng/ml of Wnt3a (cat. 31520; Peprotech) for 6hrs fixed and counter stained with DAPI as described [12]. Microscopy of enteroids was performed on an Olympus FluoView FV1200 laser-scanning confocal microscope using a 20X Apo 0.75 NA Olympus objective. Images were collected and processed as described [12].

### Cell culture

HEK293 cells were grown in DMEM supplemented with nonessential amino acids, 1000U penicillin, 1mg/ml streptomycin (Pen/Strep), 20mM L-glutamine and 10% fetal bovine serum (FBS). Caco2_BBE_ cells were grown in DMEM as above with the addition of 10mM HEPES. Caco2_BBE_ CRISPR/Cas9 MAMDC4 knockout and control cells lines, described previously [11] were grown with 3ug/ml puromycin. Media was changed every 2 to 3 days. Control and MAMDC4 knockdown (KD) MDCK cell lines have been described [10]. Cells were grown in DMEM supplemented with nonessential amino acids, 1000U penicillin, 1mg/ml streptomycin (Pen/Strep), 20mM L-glutamine and 10% fetal bovine serum (FBS) supplemented with 2ug/ml puromycin.

### MDCK transfection and immunolabeling

MDCK control and MAMDC4 KD cells were plated on coverslips in 24 well plates at 60% confluency. Cells were transfected with 3XFLAG (WT) DVL2 (cat 24803; addgene; [87]) using Lipofectamine 2000 (cat. 1168500; ThermoFisher). Mouse anti FLAG (cat. F-4042; Sigma) was used at 1:500 to detect DVL2. Alexa Flour 488 donkey anti mouse at 1:1000 (cat. A21202; ThermoFisher) was used as the secondary antibody. See Immunolabeling of Cells on Coverslips for procedure.

### SKCO transfection and immunolabeling

SKCO cells were plated on coverslips in 24 well plates at 60% confluency. Cells were transfected with 3XFLAG (WT) DVL2 (cat. 24803; addgene, [87]) and MAMDC4 (Wilson Lab) or Flag-Axin1 (cat. 109370; addgene, [88] and MAMDC4 (Wilson Lab) using Lipofectamine LTX (cat. 15338030; ThermoFisher) following the manufacturer’s instructions. Mouse anti DVL2 (cat. 390303; Santa Cruz Biotechnology) was used at 1:50 and rabbit anti MAMDC4 (cat. PA5-663000; ThermoFisher) was used at 1:50. Alexa Flour 488 donkey anti rabbit at 1:1000 (cat. A21202; ThermoFisher) and Alexa Flour 568 donkey anti mouse (cat. A16025; ThermoFishar) at 1:1000 was used as the secondary antibody to detect MAMDC4 and DVL2. For Axin1 we used rabbit anti FLAG (cat. 14793; Cell Signaling Technology (CST)) at 1:100 and mouse and MAMDC4 (5F11; Wilson Lab) at 1:2. See Immunolabeling of Cells on Coverslips for procedure.

### Confocal microscopy and colocalization analysis

For confocal microscopy, images were obtained using an Olympus Fluoview 1200 equipped with 60X Plan- Apo 1.40 NA oil immersion objective. Representative single plane images were captured with 0.5μm thickness. For colocalization analysis identical imaging parameters were used for all images obtained. Images for analysis were processed using NIH-ImageJ, where background subtraction and threshold settings were kept constant for all images analyzed. A region of interest (ROI) was selected using DVL2 or Axin1 and the Pearson’s r value was determined using Costes Threshold Regression.

### HEK293 control and MAMDC4 KD cell lines

Plasmids used to generate Lentivirus for control and MAMDC4 KD shRNA cell lines were purchased from Transomic Technologies using their shERWOOD-Lentiviral-UltramiR-shRNA vector with hCMV promoter and puromycin selection. Sequence for the MAMDC4 targeting shRNA are 3’ AAACACGATCTGGAACTCCTTG, 5’ AAAGGAGTTCCAGATCGTGTTT, and Loop TAGTGAAGCCACAGATGTA, for control shRNA 3’ ATGCTTGCATACTTCTGCCTG, 5’ AAGGCAGAAGTATGCAAGCAT, and Loop TAGTGAAGCCACAGATGTA. Production of viral particles and generation HEK293 cell lines were created using the manufacturer’s instructions.

### Immunolabeling of cells on coverslips

Cells were fixed in 4% PFA for 20min 48hrs post transfection. Cells were washed 3 X with PBS, aldehydes were quenched with 50mM NH_4_Cl for 15min, washed with PBS, and blocked using 0.2% saponin (cat. S-7900; Sigma), 20% FBS in PBS for 30min. For SKCO labeling cells were permeabilized with 0.1% TX-100 on PBS for 10min prior to blocking. Primary antibody, was diluted in 0.2% saponin/FBS blocking solution and incubated for 2hrs. Following 3 X 5min was in PBS, cells were incubated with secondary antibody for 45min at 1:1000 in saponin/FBS block, washed with PBS and mounted to slides with ProLong Gold containing DAPI (cat.P36931; ThermoFisher).

### Caco2_BBE_ proliferation

Cells were plated at 2 X 10^5^ cells/coverslip and fixed 48hrs later for pHH3 (cat. 9710; CST) labeling as previously described [10]. For the MAMDC4 rescue experiment 1 X 10^5^ Caco2_BBE_ were plated on coverslips and transfected with MAMDC4 24hrs after plating at a PEI:DNA ratio of 3:1 with and 1.5μg of PEI and 500ng of DNA. Media was replaced 16hrs post-transfection and cells were fixed 24hrs after media change. Three images were collected for each coverslip. Cell number for each image ranged from 100 to 300 for each image. 500 to 1000 cells per condition were counted and the rescue was repeated 3 times.

### NeutrAvidin pull down

MAMDC4 was overexpressed in HEK293 cells in 10cm plates as described above. When cultures reached 90% confluence plates were transferred to room temperature (RT), washed 2 X with PBS and complete media with 0.5mg/ml Sulfo-NHS-SS-Biotin was added (cat. 21331; ThermoFisher). Cells were incubated 30min at RT, washed with 50mM Tris pH8.0 and 4 X with cold PBS. Cells were lysed in NP-40 lysis buffer [10] and 950ug of total protein in equal concentration was used for each pull down. Lysates were incubated with NeutrAvidin Agarose Resin (cat. 29200; ThermoFisher) for 1hr at 4°C and washed 4 X with lysis buffer. Samples were resuspended in 30ul of sample buffer, and immunoblot was probed with antibodies for LRP6 (cat. 2560; CST). Immunoblots were imaged using a Licor-Odyssey infrared imager. LRP6 pull down was normalized to input levels.

### Tissue labeling

The intestine was processed for histochemical analysis as described [11]. Antigen retrieval was performed with 10mL Tris with 1mM EDTA and 0.05% Tween-20 pH 9. Tissue was blocked in TBST with 5% fetal bovine serum. Primary antibody, rabbit anti-YAP (cat. 14074; CST) was used at 1:100 in blocking solution and incubated overnight at 4°C. Slides were incubated with 3% H_2_O_2_ prior to secondary antibody addition. Signal Boost was used as secondary detection (cat. 8114S; Cell Signaling Technology) and the DAB detection kit (cat. 8059S; Cell Signaling Technology) was used for development of signal. Tissue was dehydrated through an ethanol series and mounted using SignalStain mounting media (cat. 14177S; CST).

### Microscopy

For light microscopy, images were obtained using a Leica DMI600 microscope and Leica LAS-x software and processed as previously described [11].

### Wnt3a conditioned media

L-Wnt3a (cat. CRL-2647, ATCC) and control L-Cells (cat. CRL-2648, ATCC) were grown in DMEM with 10% FBS, NEAA, Pen/Strep, and 400μg/ml G418. Prior to collection, cells were grown in media without G418.

### Immunoprecipitation

For immunoprecipitation, cells were grown to 90% confluence in 10cm dishes, treated with 50% Wnt3a conditioned media for 6hrs and lysed in an NP-40 lysis buffer as described [10]. Lysates were precleared with Dynabeads™ Protein G (cat. 1003D; ThermoFisher) for 1hr at 4°C. Protein G beads were incubated in 200ul of binding buffer with 5μg of antibodies for 10min at room temperature. For MAMDC4 immunoprecipitation, beads were incubated with 1.5ml of hybridoma supernatant containing anti-MAMDC4 (Wilson Lab) for 1hr at room temperature. For AMOT immunoprecipitation, beads were incubated with 5ug of rabbit anti AMOT (cat. 24550–1; ProteinTech). Beads bound with antibody were washed and then precleared lysate was added. Lysate and antibody-bound beads were incubated overnight at 4°C with mixing. Beads were washed 3X with lysis buffer and proteins eluted in SDS sample buffer. Immunoprecipitations were repeated 3 to 5 times. Equal amounts of protein (500-800ug) at equal concentrations were used for the immunoprecipitation experiments and 1/50^th^ of lysate was used as the input. 1.2mg– 1.5mg of total protein was used for AMOT immunoprecipitation in control and MAMDC4 KD lysates. Immunoblots were imaged as described below.

### MAMDC4 constructs

Generation of the MAMDC4 amino acid substitutions and chimera constructs used were previously described [32, 33]. For all overexpression control experiments, cells were transfected with empty vector.

### Luciferase reporter assay

Caco2_BBE_ and HEK293 cells used for luciferase reporter assays were plated at 60% confluence in 24-well plates. Cells were transfected with the Super TOPFlash luciferase reporter and renilla control plasmids [31]. PEI was used for transfection at a PEI:DNA ratio of 4:1 for Caco2 and 3:1 for HEK293 as previously described [11]. 0.5μg DNA was used for transfection in 24 well plates, media was replaced after 16 hours. Transfections were done in triplicate and cells were lysed using Dual-Glo Luciferase Assay buffer according to manufacturer’s instructions (cat. 2920; Promega). Lysate from each well was transferred to 2 wells of a 96 well plate and Luciferase signal was measured using a SpectraMax luminometer. Transfections were repeated 3 or 4 times to obtain biological replicates.

### Protein analysis

Cells were collected at ~90% confluence, lysed in RIPA (20mM Tris pH 7.4, 1% Triton X-100, 1% deoxycholate, 0.1% SDS, 100mM NaCl) containing protease and phosphatase inhibitors and analyzed as previously described [11]. Luciferase were collected in Luciferase assay buffer and resuspended in 2X SDS sample buffer. The following antibodies were used: mouse anti-MAMDC4 1:3 (Wilson Lab), rabbit anti-actin (cat. 4970; CST), mouse anti-total β-catenin (cat. 610153; BD Biosciences), rabbit anti total β-catenin (cat. 8480; CST) rabbit anti-non-phospho (active) β-catenin (cat. 19807; CST), rabbit anti-Axin1 (cat. 2074; CST), rabbit anti-LRP6 (cat. 2560; CST), rabbit anti-phospho-LRP6 (Ser 1490) (cat. 2568; CST), rabbit anti-total GSK (cat. 12456; CST), rabbit anti-phospho-GSK3β (Ser 9) (cat. 5558; CST), rabbit anti-DVL2 (cat. 3224; CST), rabbit anti-MST (cat. 3682; CST), rabbit anti-LATS (cat. 3477; CST), rabbit anti-phospho-LATS (cat. 8654; CST), rabbit anti-YAP (cat. 14074; CST), rabbit anti-phospho YAP (cat. 13008; CST), rabbit anti AMOT (cat. 24550–1; ProteinTech). Immunoblots were imaged using a Licor-Odyssey infrared imager. Protein expression was normalized to actin levels.

### Statistical analysis

Statistical comparisons were performed using Prism6 (GraphPad) and analyzed using unpaired Student’s t-test and One-way ANOVA with Tukey’s multiple comparison. Error bars represent the standard deviation.

## Supporting information

S1 Fig MAMDC4 deletion inhibits cell proliferation. (A) CRISPR/Cas9 Caco2_BBE_ control and MAMDC4 KO cells were grown on coverslips for 48 hours and labeled with antibodies against pHH3 and counterstained with DAPI. pHH3 labeling (arrows) is decreased in MAMDC4 KO cells and MAMDC4 overexpression increases pHH3 labeling in MAMDC4 KO cells (arrows). *Scale bar*: 10μm.
(TIF)

S2 Fig MAMDC4 cytoplasmic domain plays a role in MAMDC4 regulation of Wnt/β-catenin activity. Lysates of HEK293 cells overexpressing MAMDC4 point mutations used for measuring luciferase were analyzed by immunoblot for MAMDC4 expression.
(TIF)

S3 Fig Decreased DVL2 puncta formation MAMDC4 KD. MDCK cells expressing control and or shRNA directed to MAMDC4 were transfected with DVL2. 48 hours post transfection cells were labelled with antibodies to DVL2 (green). There is a decrease in the percentage on cells with distinct DVL2 puncta (arrow) in MAMDC4 KD cells. 250–350 cells were assessed per condition, n = 3. *P<0.05. Statistical significance was determined by unpaired Student’s t-test. *Scale bar*: 25μm.
(TIF)

S4 Fig HEK293 cell expressing control and shRNA to MAMDC4 were analyzed by immunoblot for MAMDC4 KD and active β-catenin. MAMDC4 and active β-catenin in HEK293 cells with MAMDC4 knock down (KD).
(TIF)

S1 File Request for change to authorship. (DOCX)

S1 Raw images (PDF)
