# Supplementary figures and images for: Regulation of YAP and Wnt signaling by the endosomal protein MAMDC4
Source: PLoS One. 2024 May 24;19(5):e0296003. doi: 10.1371/journal.pone.0296003 (PMC11125477; doi:10.1371/journal.pone.0296003)

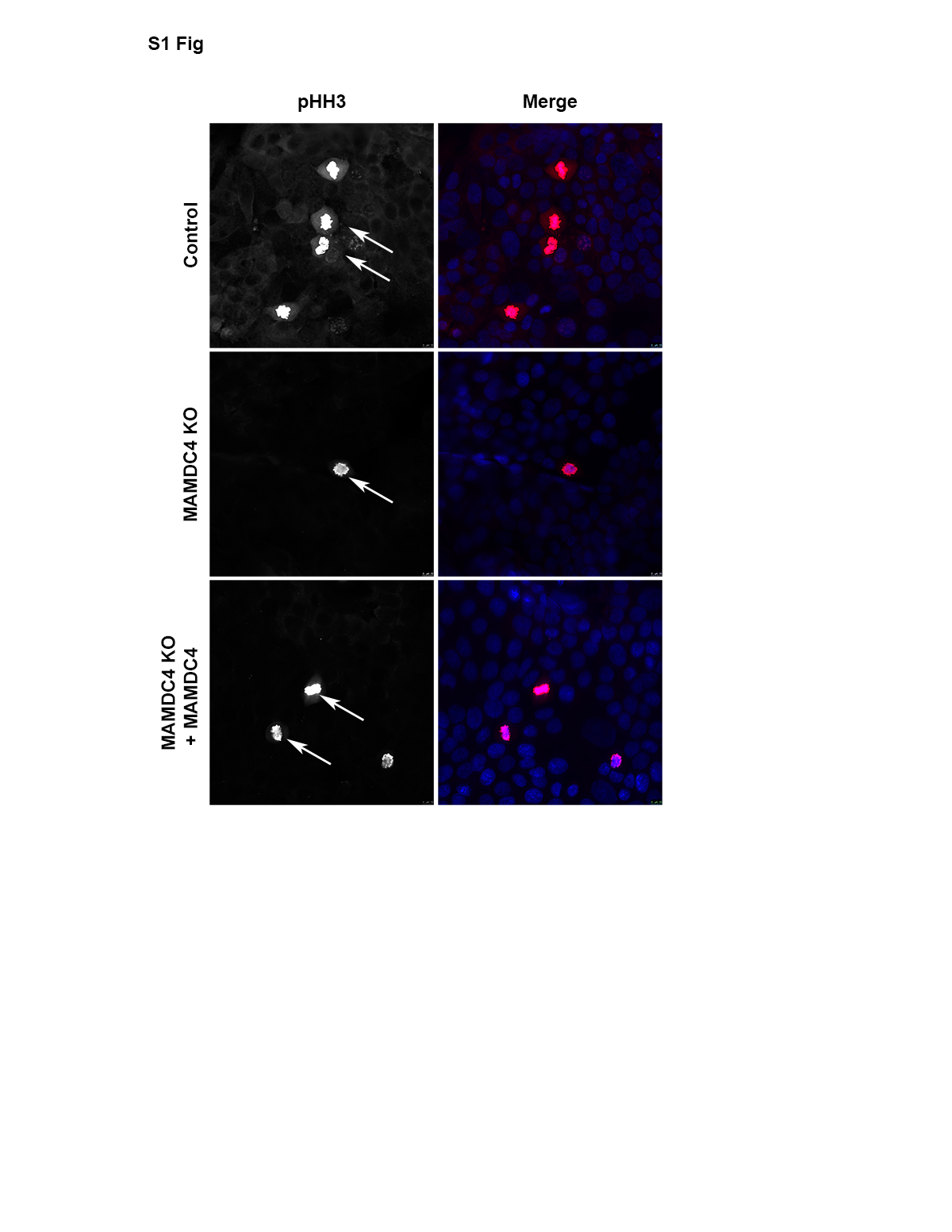

Supplement: S1 Fig — (A) CRISPR/Cas9 Caco2BBE control and MAMDC4 KO cells were grown on coverslips for 48 hours and labeled with antibodies against pHH3 and counterstained with DAPI. pHH3 labeling (arrows) is decreased in MAMDC4 KO cells and MAMDC4 overexpression increases pHH3 labeling in MAMDC4 KO cells (arrows). Scale bar: 10μm. (TIF) [file pone.0296003.s001.tif]

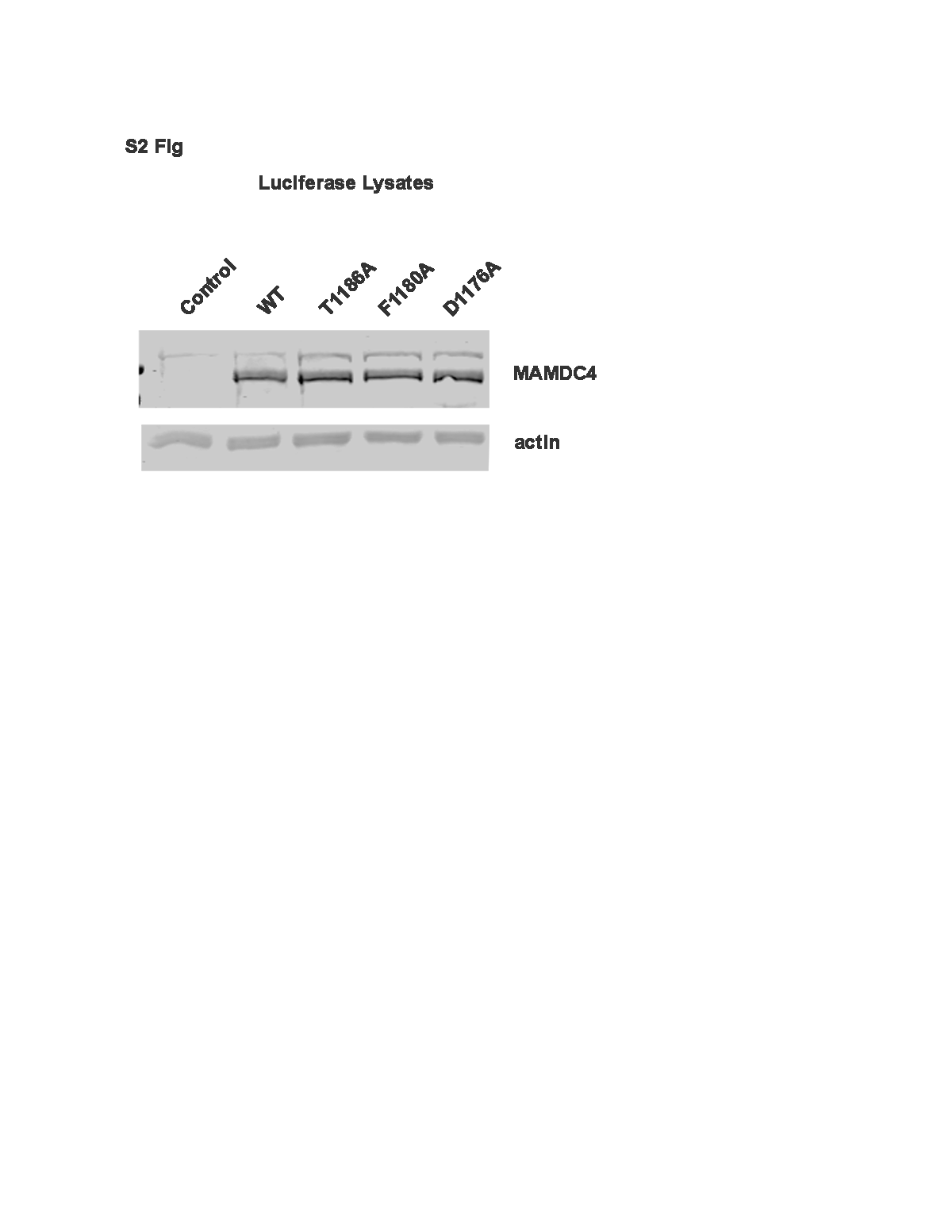

Supplement: S2 Fig — Lysates of HEK293 cells overexpressing MAMDC4 point mutations used for measuring luciferase were analyzed by immunoblot for MAMDC4 expression. (TIF) [file pone.0296003.s002.tif]

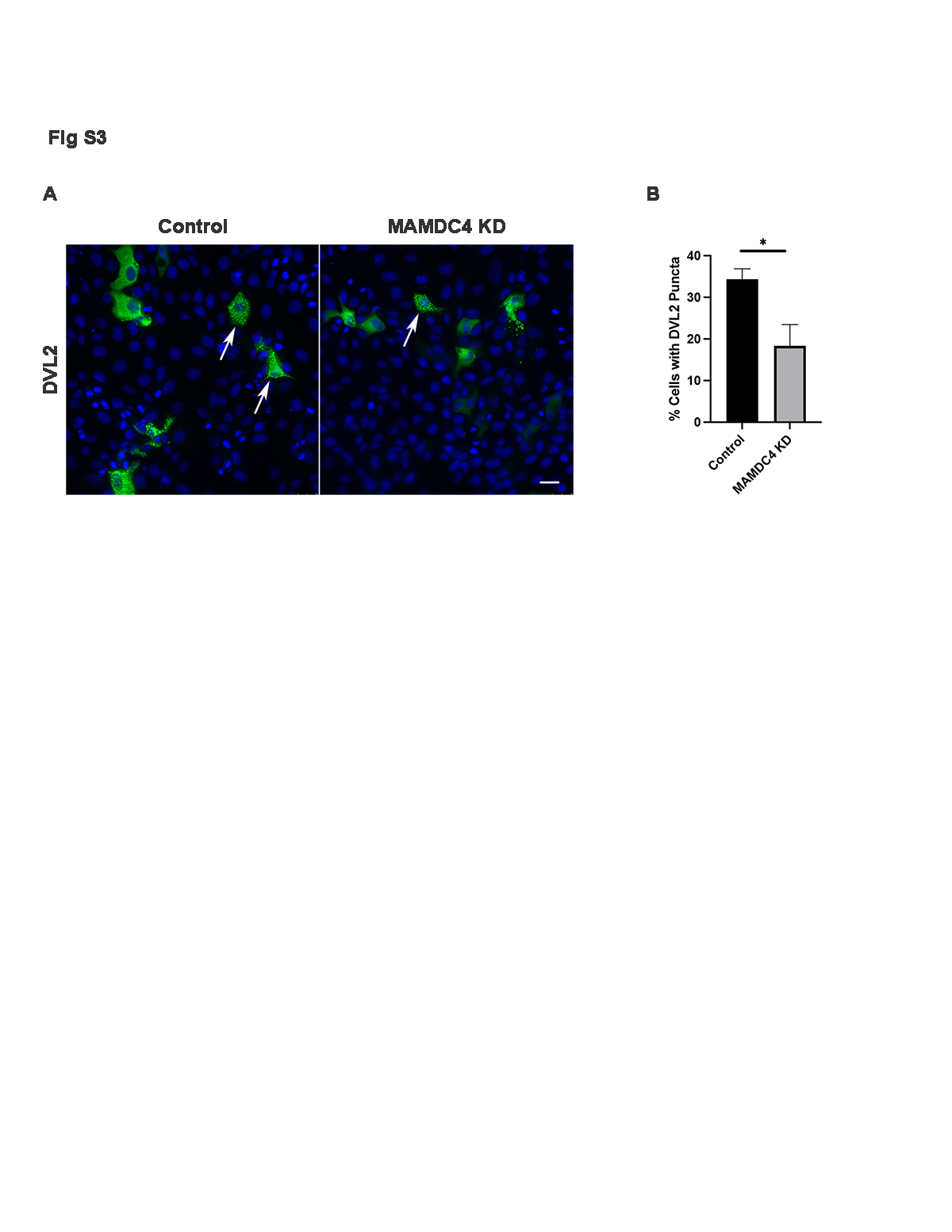

Supplement: S3 Fig — MDCK cells expressing control and or shRNA directed to MAMDC4 were transfected with DVL2. 48 hours post transfection cells were labelled with antibodies to DVL2 (green). There is a decrease in the percentage on cells with distinct DVL2 puncta (arrow) in MAMDC4 KD cells. 250–350 cells were assessed per condition, n = 3. *P<0.05. Statistical significance was determined by unpaired Student’s t-test. Scale bar: 25μm. (TIF) [file pone.0296003.s003.tif]

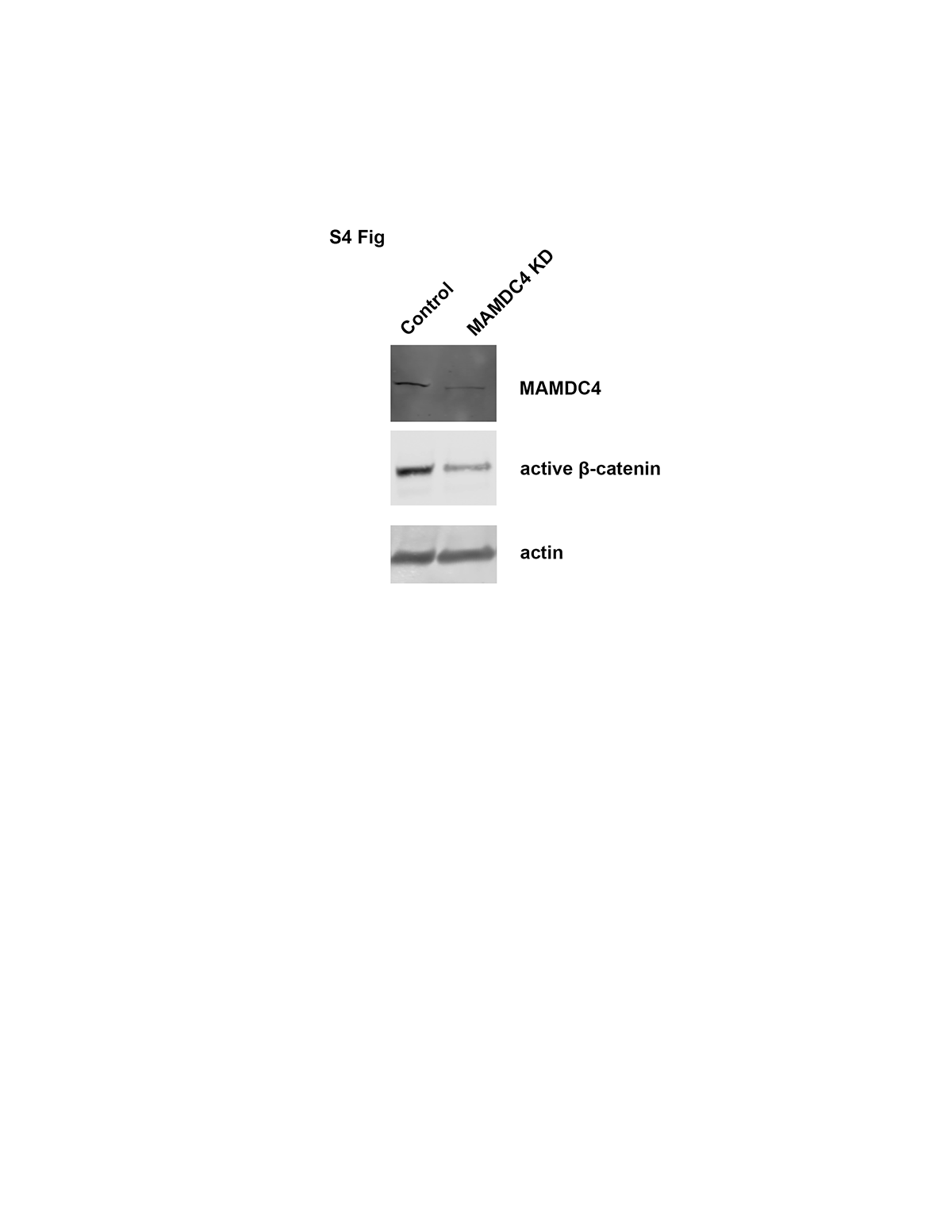

Supplement: S4 Fig — MAMDC4 and active β-catenin in HEK293 cells with MAMDC4 knock down (KD). (TIF) [file pone.0296003.s004.tif]
