# Supplementary material for: Regulation of YAP and Wnt signaling by the endosomal protein MAMDC4
Source: PLoS One. 2024 May 24;19(5):e0296003. doi: 10.1371/journal.pone.0296003 (PMC11125477; doi:10.1371/journal.pone.0296003)
Supplement: S1 File — (DOCX) [file pone.0296003.s005.docx]

# Request for Change to Authorship

Check to confirm you have read [*PLOS ONE*’s authorship policy](http://journals.plos.org/plosone/s/authorship).

The authorship criteria for *PLOS ONE*, summarized below, are based on those outlined by the International Committee of Medical Journal Editors (ICMJE):

1. Conception and design of the work, acquisition of data, or analysis and interpretation of data
2. Drafting the article or revising it critically for important intellectual content
3. Final approval of the version to be published
4. Agreement to be accountable for all aspects of the work

Authors should meet all of the criteria; the contributions of all authors will be disclosed in the final publication. Any contributions that fall short of the criteria should be named in the Acknowledgments section of the manuscript. It is your responsibility to ensure that anyone named in the Acknowledgments consents to being named.

Check to confirm that all authors (including those to be added or removed) consent to the changes detailed below.

| **Reason for change in author list**  Please briefly describe the reason for adding/removing an author. | Three authors were added when resubmitting the manuscript. All three authors preformed experiments and collected data critical to to the resubmisstion |
| --- | --- |

# Final manuscript information

| **Manuscript number**  e.g., PONE-D-17-00000 | PONE-D_23_05936R2 |
| --- | --- |
| **Complete author list, in correct order**  Please note any equal contributors with asterisks (*) or hashes (#) | Christopher M. Cox, Meng-Han Wu, Marco Padilla-Rodriguez, Isabella Blum, Samina Momtaz, Stefanie A.T. Mitchell1and Jean M. Wilson |
| [**Financial Disclosure**](http://journals.plos.org/plosone/s/disclosure-of-funding-sources) – including any additions/deletions necessary due to the change in authorship |  |
| [**Competing Interests**](http://journals.plos.org/plosone/s/competing-interests) – including any additions/deletions necessary due to the change in authorship |  |
| [**Acknowledgments statement**](http://journals.plos.org/plosone/s/submission-guidelines#loc-acknowledgments)  Please acknowledge any removed authors if they contributed to the study in any way, as well as members of any author groups who do not meet our authorship criteria. |  |

# Adding authors

## Individual author addition #1

| **Full name** | Samina Momtaz |
| --- | --- |
| **Email address** | saminamomtaz@arizona.edu |
| **Full affiliation** | Department of Cellular and Molecular Medicine, University of Arizona, Tucson, AZ 85724 |

| This person contributed to **all** of the following:   1. Conception and design of the work, acquisition of data, or analysis and interpretation of data 2. Drafting the article or revising it critically for important intellectual content 3. Final approval of the version to be published 4. Agreement to be accountable for all aspects of the work |  |
| --- | --- |
| **Specific contributions:** | |
| Conceptualization |  |
| Data Curation |  |
| Formal Analysis |  |
| Funding Acquisition |  |
| Investigation |  |
| Methodology |  |
| Project Administration |  |
| Resources |  |
| Software |  |
| Supervision |  |
| Validation |  |
| Visualization |  |
| Writing – Original Draft Preparation |  |
| Writing – Review & Editing |  |

## Individual author addition #2 (if applicable)

| **Full name** |  |
| --- | --- |
| **Email address** |  |
| **Full affiliation** |  |

| This person contributed to **all** of the following:   1. Conception and design of the work, acquisition of data, or analysis and interpretation of data 2. Drafting the article or revising it critically for important intellectual content 3. Final approval of the version to be published 4. Agreement to be accountable for all aspects of the work |  |
| --- | --- |
| **Specific contributions:** | |
| Conceptualization |  |
| Data Curation |  |
| Formal Analysis |  |
| Funding Acquisition |  |
| Investigation |  |
| Methodology |  |
| Project Administration |  |
| Resources |  |
| Software |  |
| Supervision |  |
| Validation |  |
| Visualization |  |
| Writing – Original Draft Preparation |  |
| Writing – Review & Editing |  |

## Author group addition (if applicable)

| **Group or consortium name** |  |
| --- | --- |
| **Author who represents group** |  |

# Removing authors

## Author removal #1

| **Full name** |  |
| --- | --- |

| This person **did not** contribute to all of the following:   1. Conception and design of the work, acquisition of data, or analysis and interpretation of data 2. Drafting the article or revising it critically for important intellectual content 3. Final approval of the version to be published 4. Agreement to be accountable for all aspects of the work |  |
| --- | --- |
| This person consents to being acknowledged in the published paper. |  |

## Author removal #2 (if applicable)

| **Full name** |  |
| --- | --- |

| This person **did not** contribute to all of the following:   1. Conception and design of the work, acquisition of data, or analysis and interpretation of data 2. Drafting the article or revising it critically for important intellectual content 3. Final approval of the version to be published 4. Agreement to be accountable for all aspects of the work |  |
| --- | --- |
| This person consents to being acknowledged in the published paper. |  |
