# Supplementary material for: Regulation of YAP and Wnt signaling by the endosomal protein MAMDC4
Source: PLoS One. 2024 May 24;19(5):e0296003. doi: 10.1371/journal.pone.0296003 (PMC11125477; doi:10.1371/journal.pone.0296003)

Figure 1 raw images

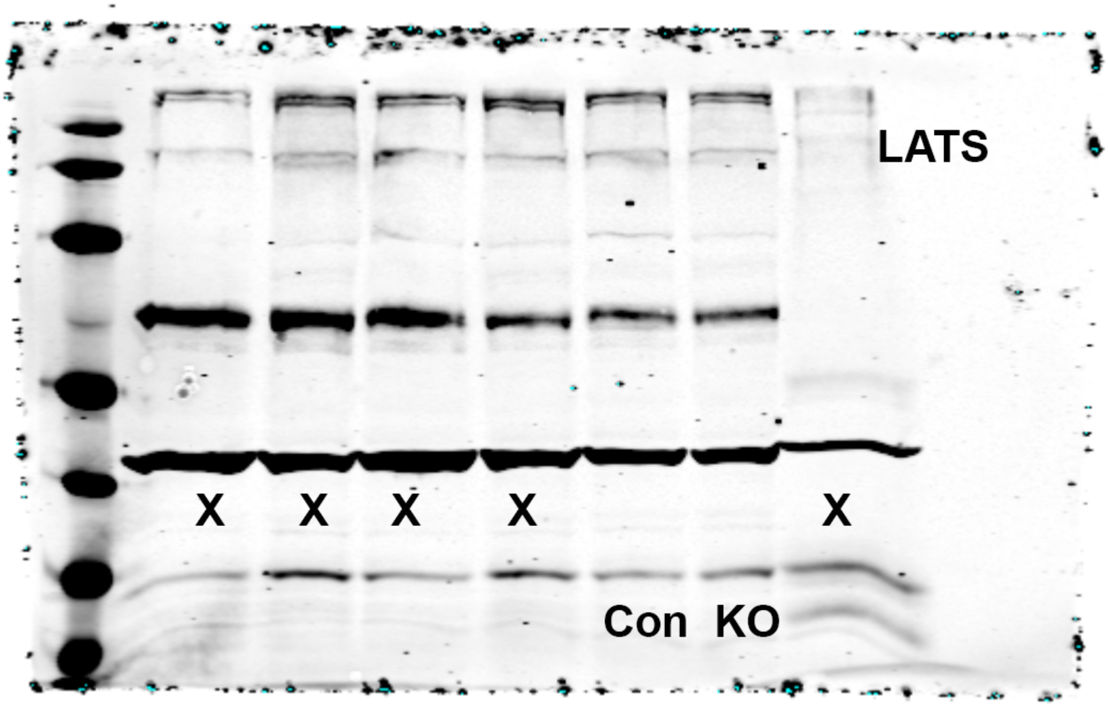

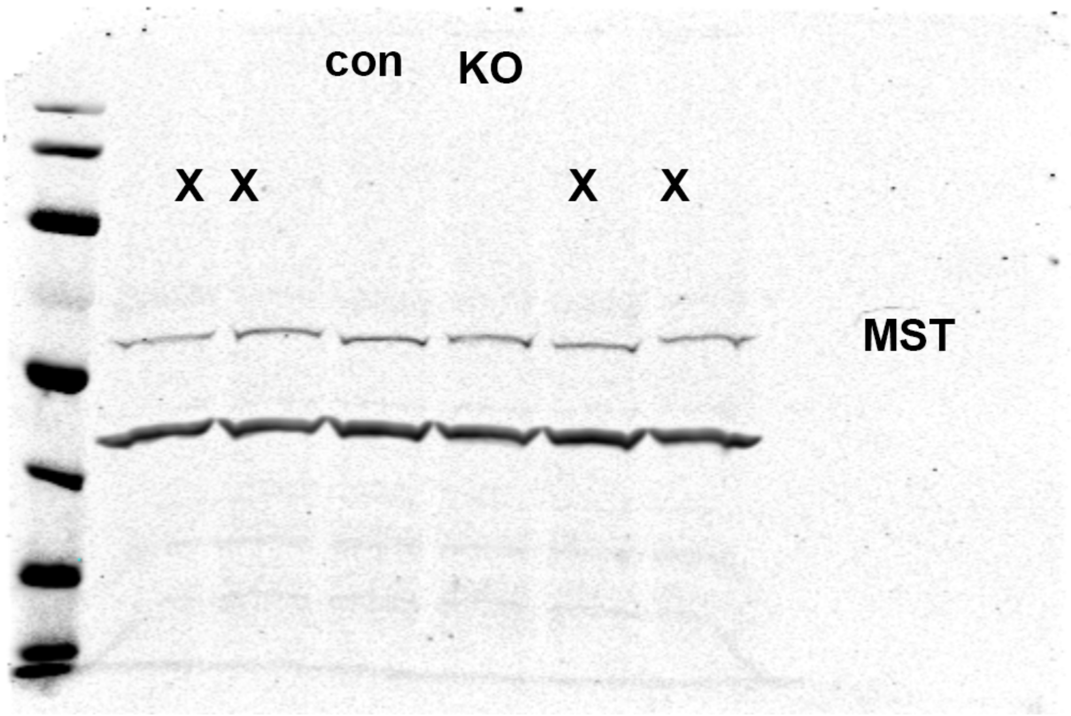

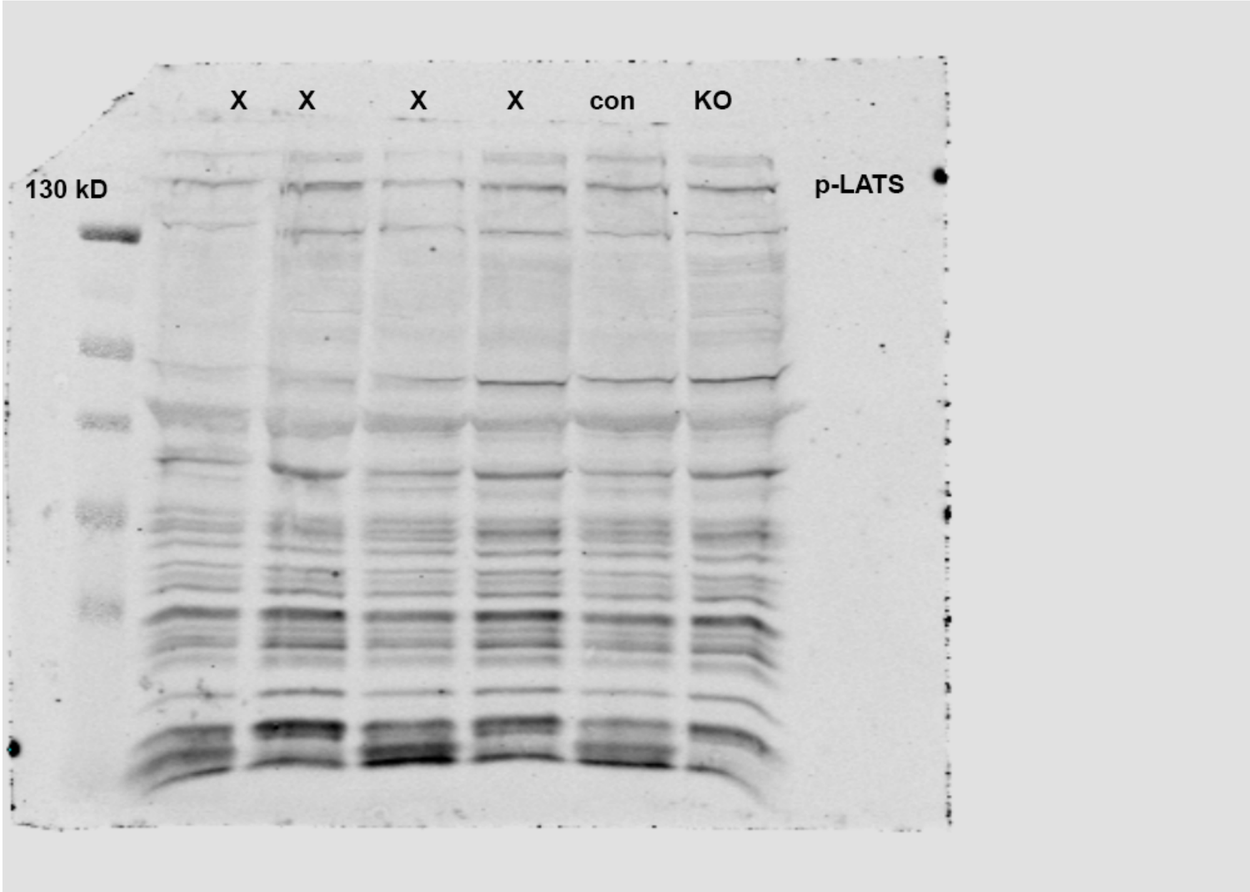
X X X X con KO
130 kD
p-LATS

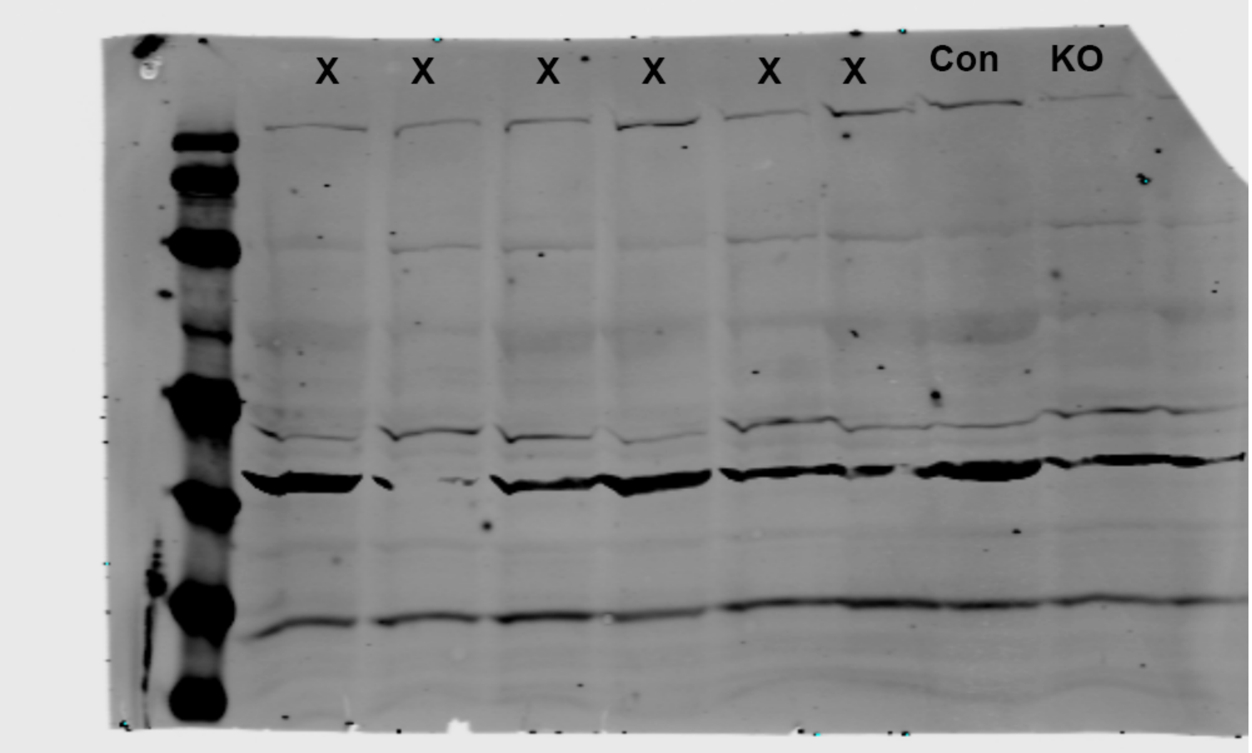
X X X X X X Con KO

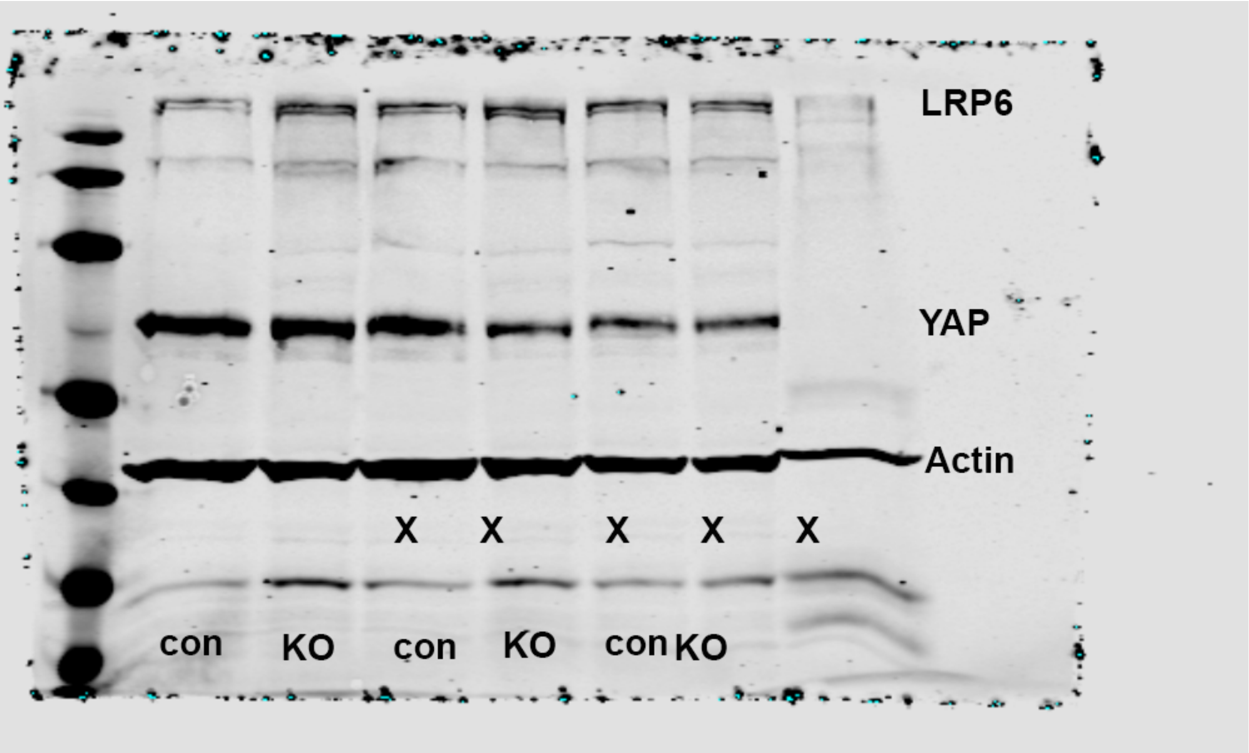

LRP6
YAP
Actin
X X X X X
con KO con KO con KO

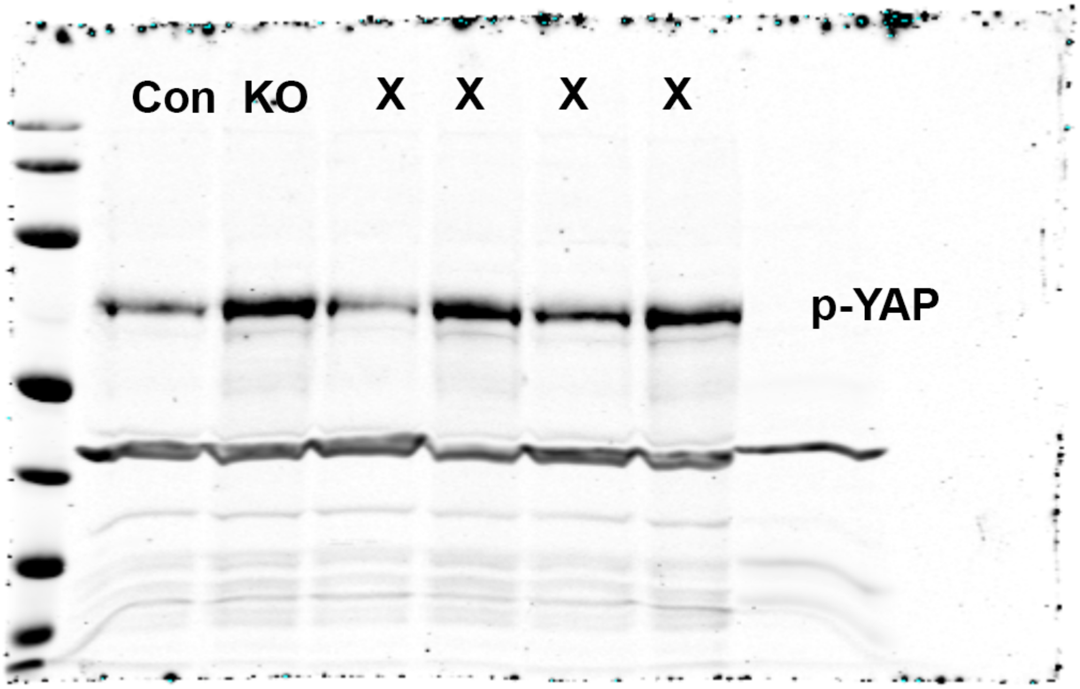

Con KO X X X X
p-YAP

Figure 2 raw images

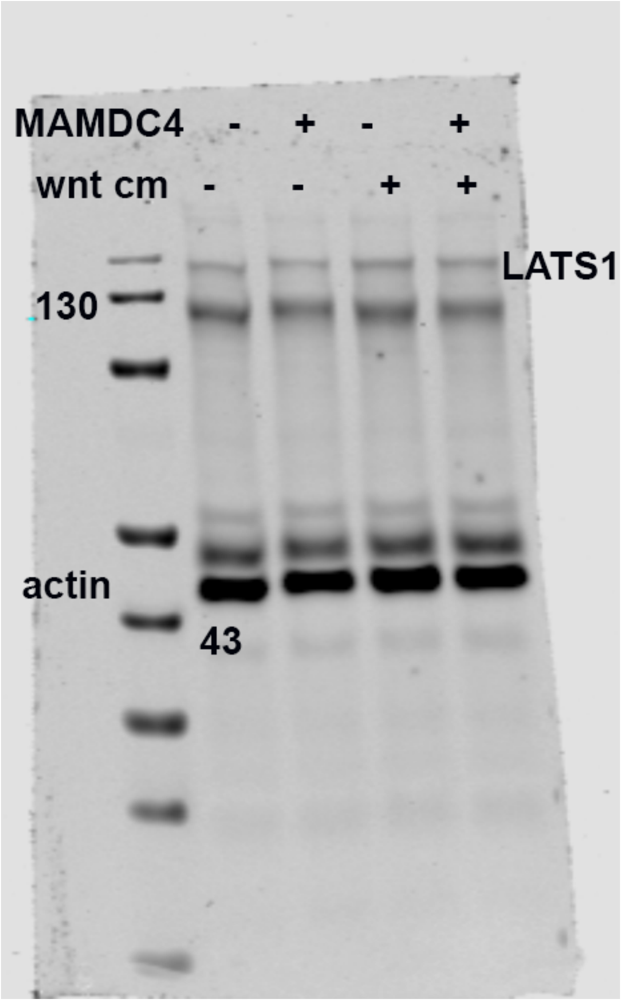

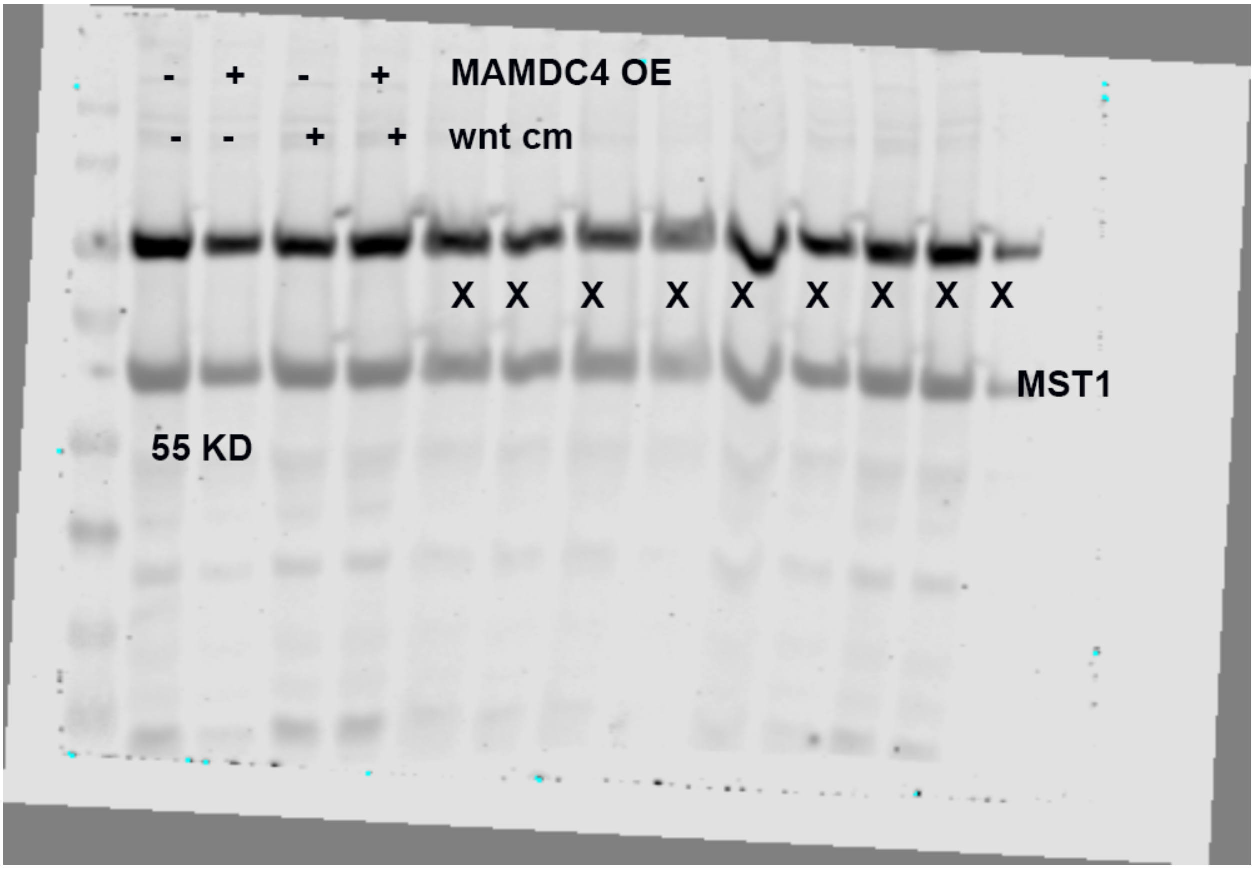
- + - + MAMDC4 OE
- - + + wnt cm
X X X X X X X X X
MST1
55 KD

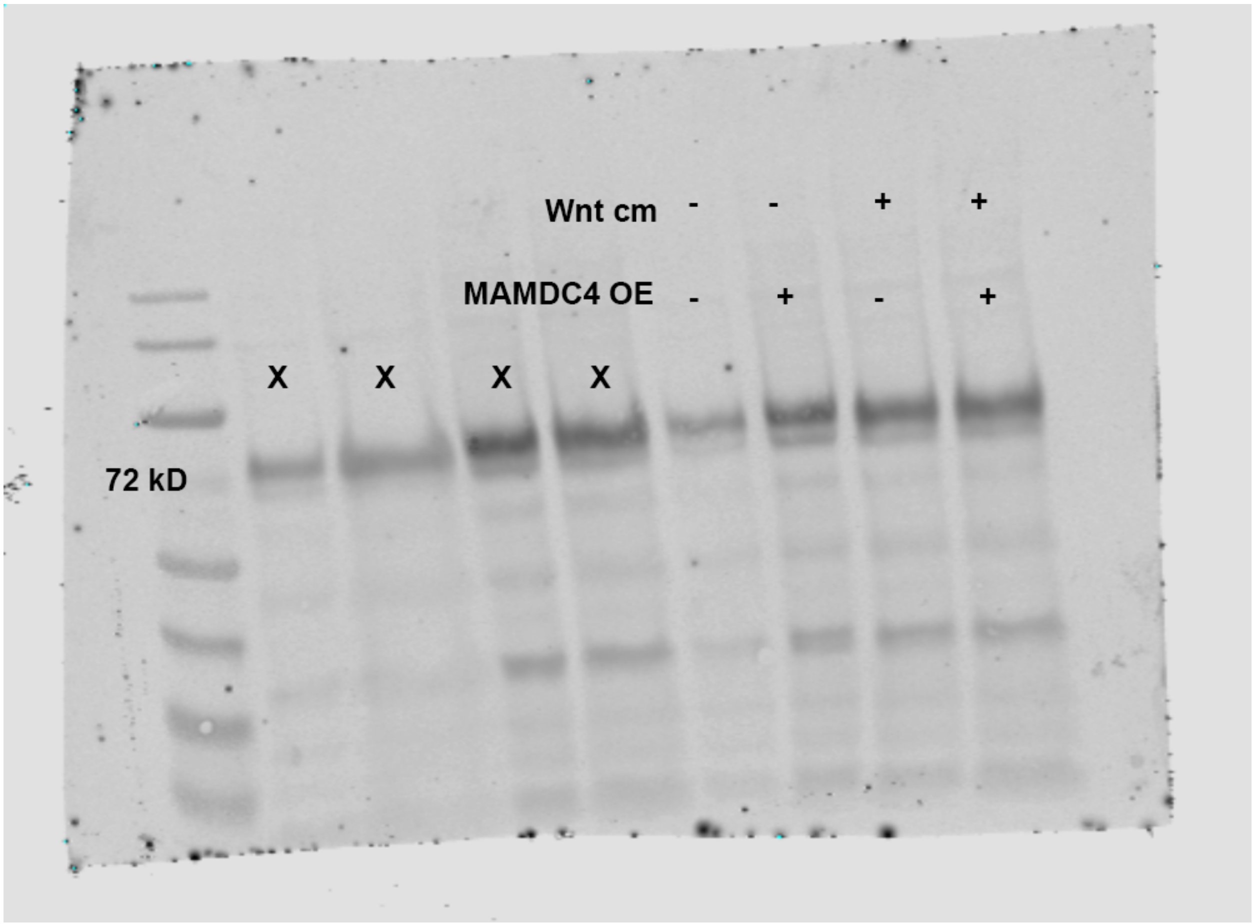
Wnt cm - - + +
MAMDC4 OE - + - +
X X X X
72 kD

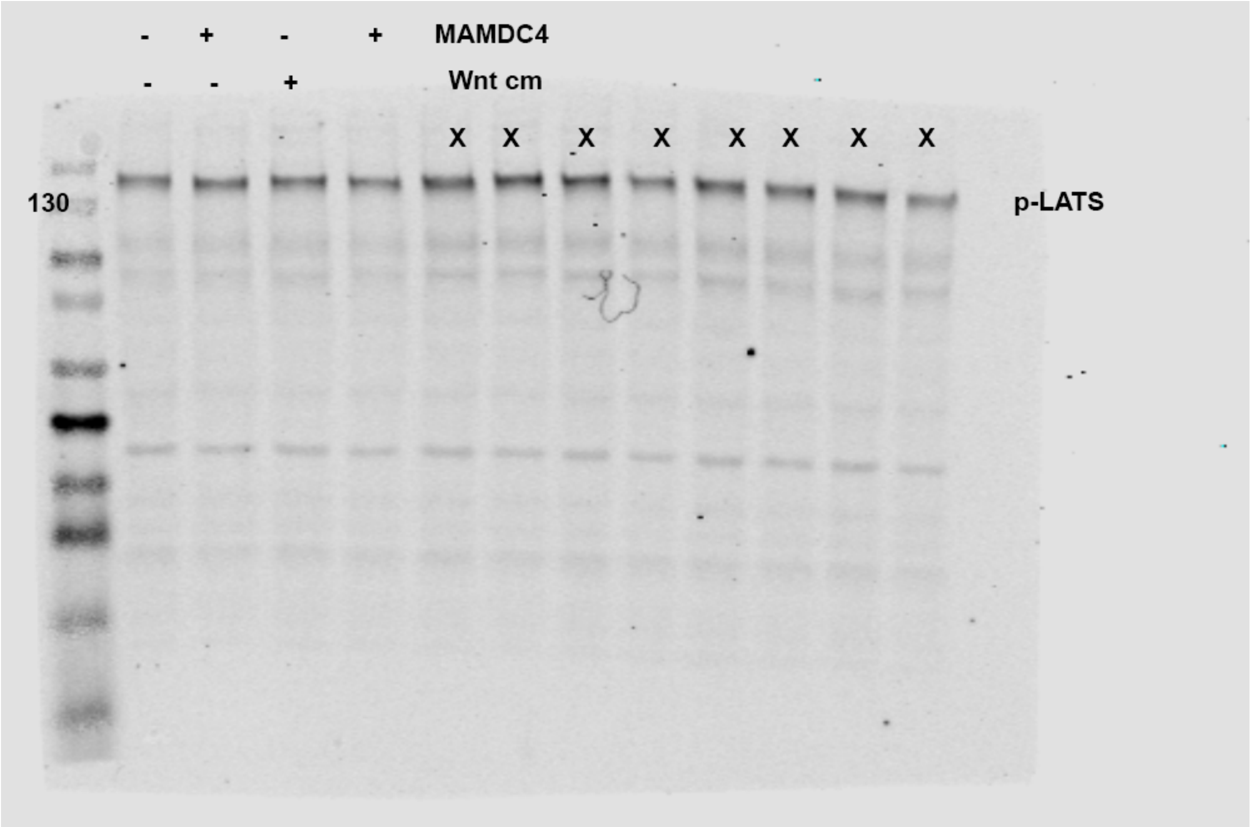
- + - + MAMDC4
- - + Wnt cm
X X X X X X X X
130
p-LATS

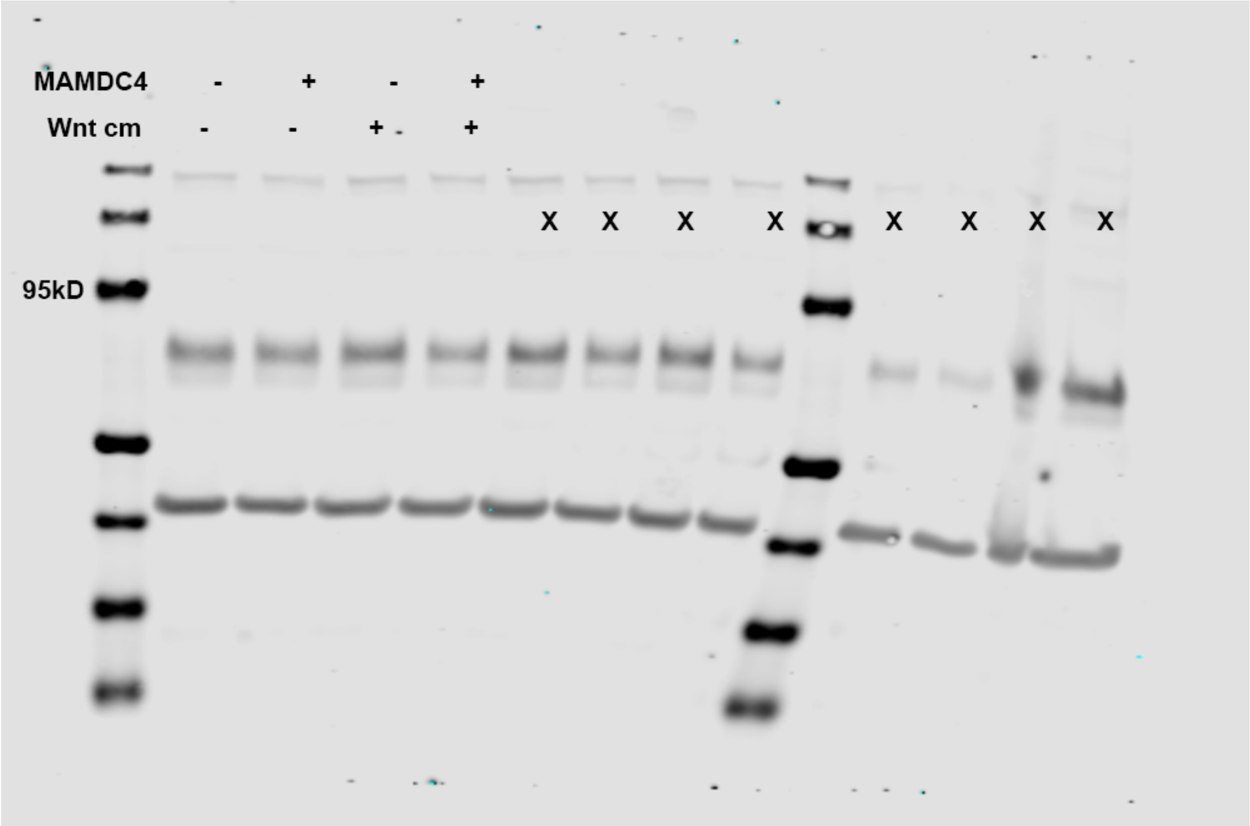
MAMDC4 - + - +
Wnt cm - - + +
X X X X X X X X
95kD

Figure 3 raw images

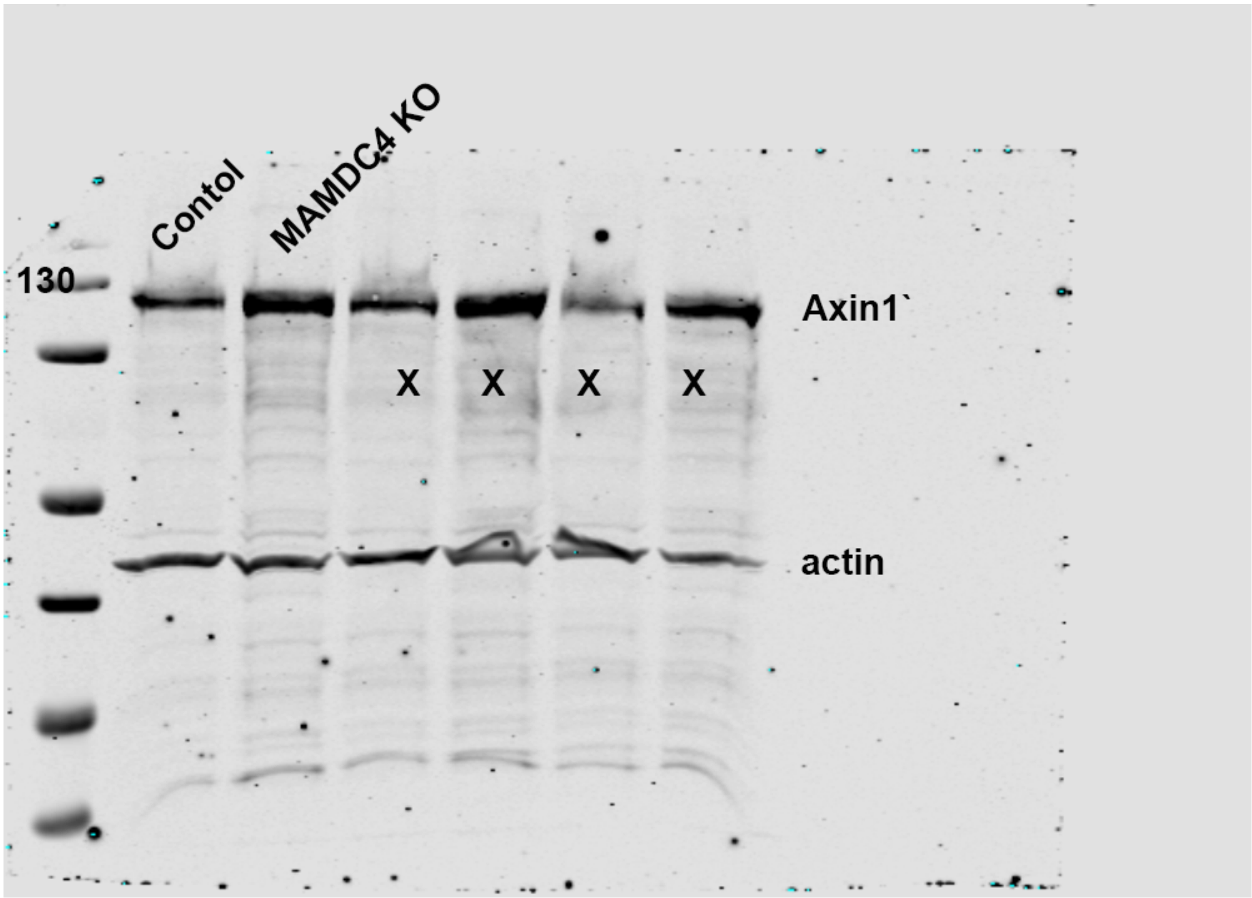

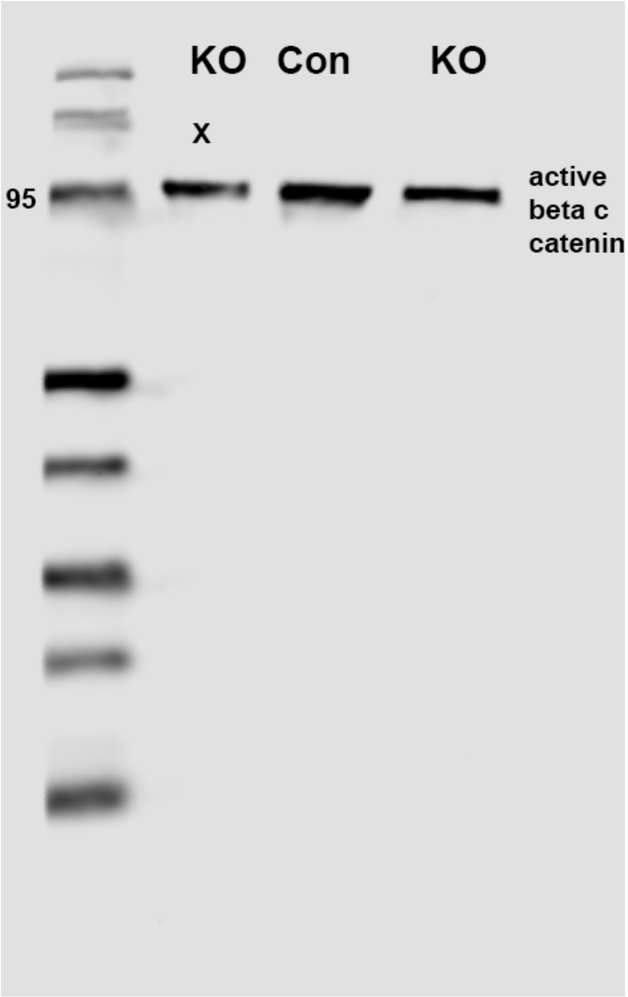
KO
Con
KO
x
95
active
beta c
catenin

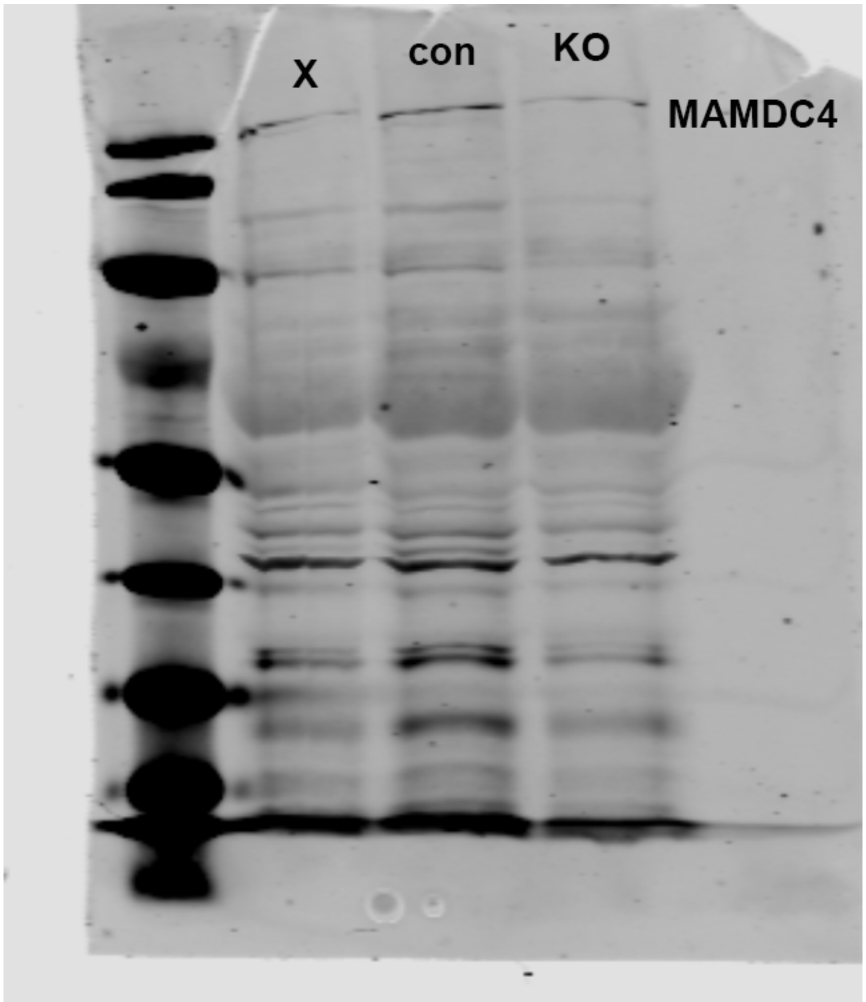
X
con
KO
MAMDC4

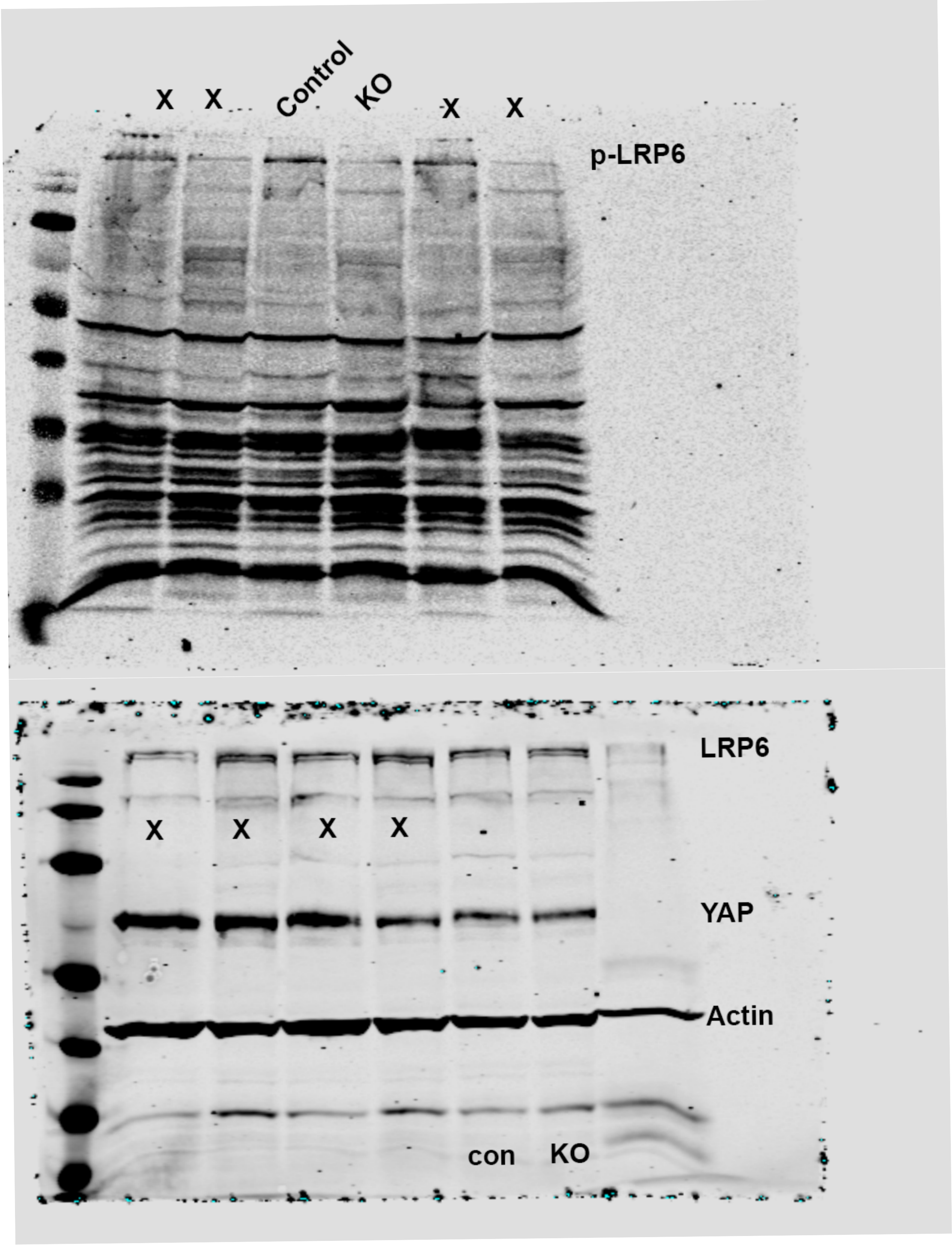

X
X
Control
KO
X
X
p-LRP6
LRP6
X
X
X
X
YAP
Actin
con
KO

Figure 4 raw images

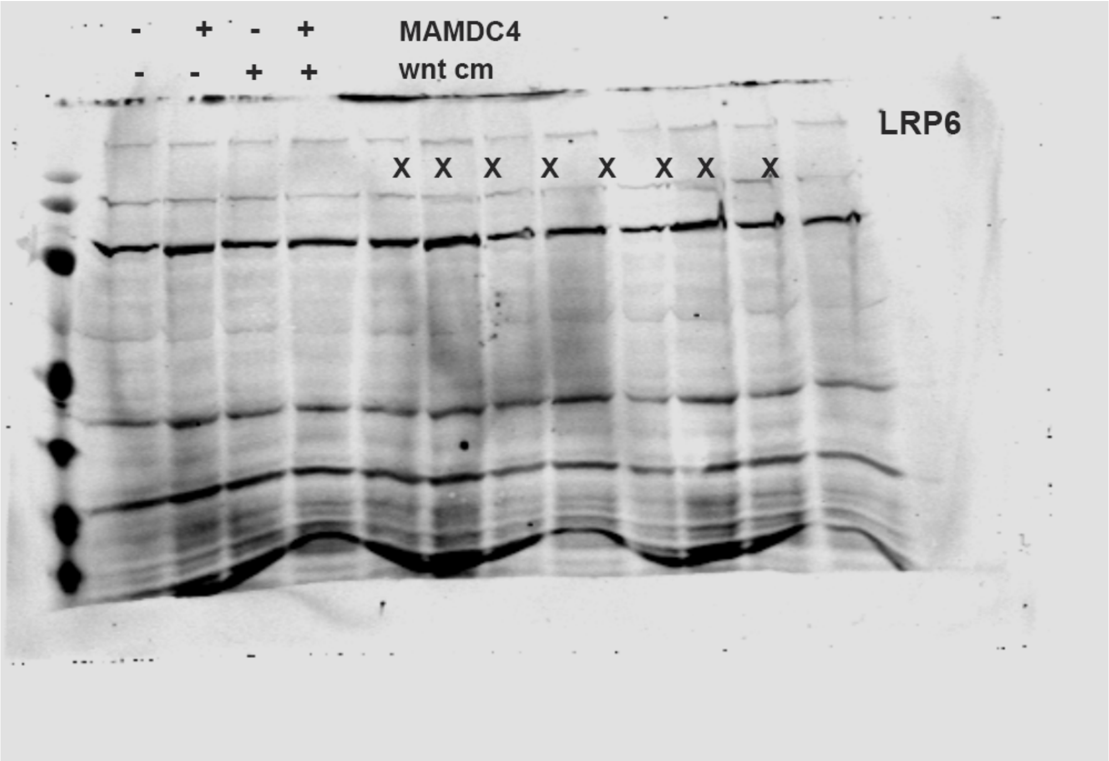

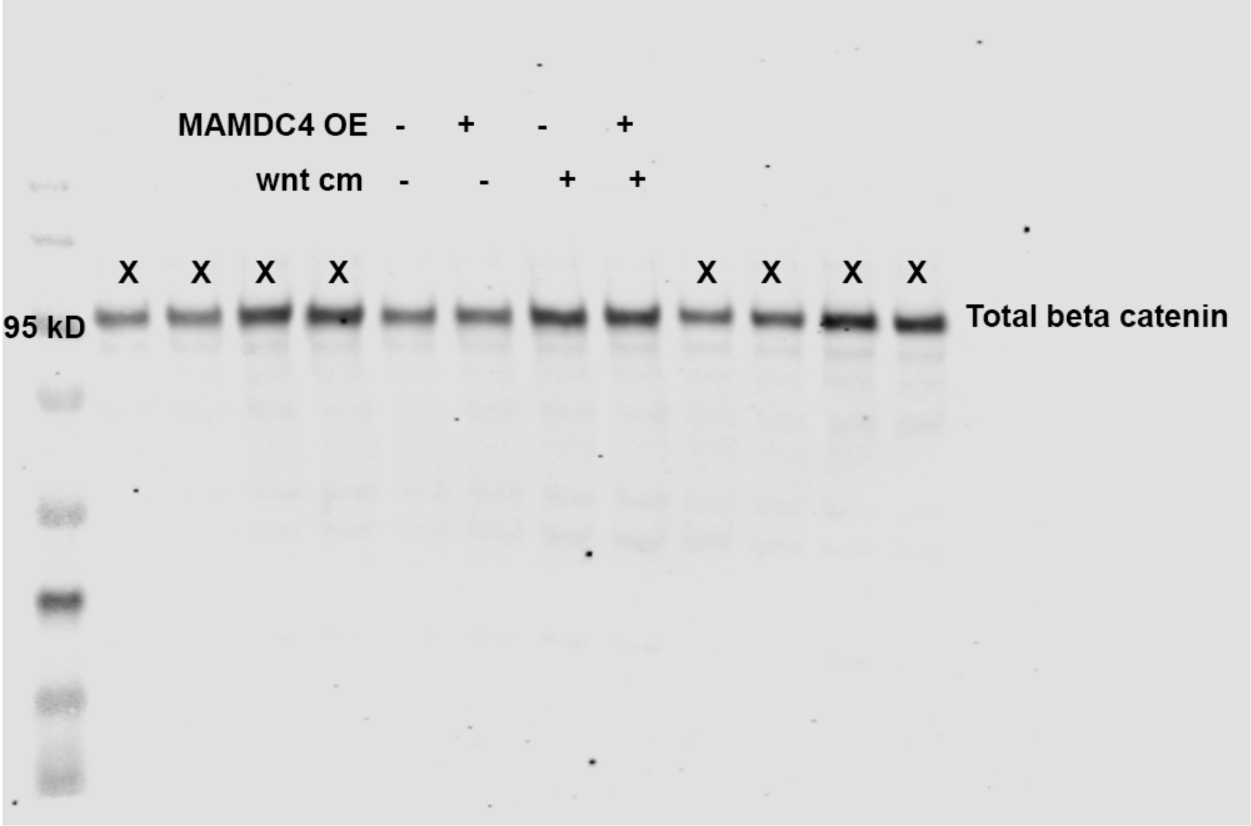

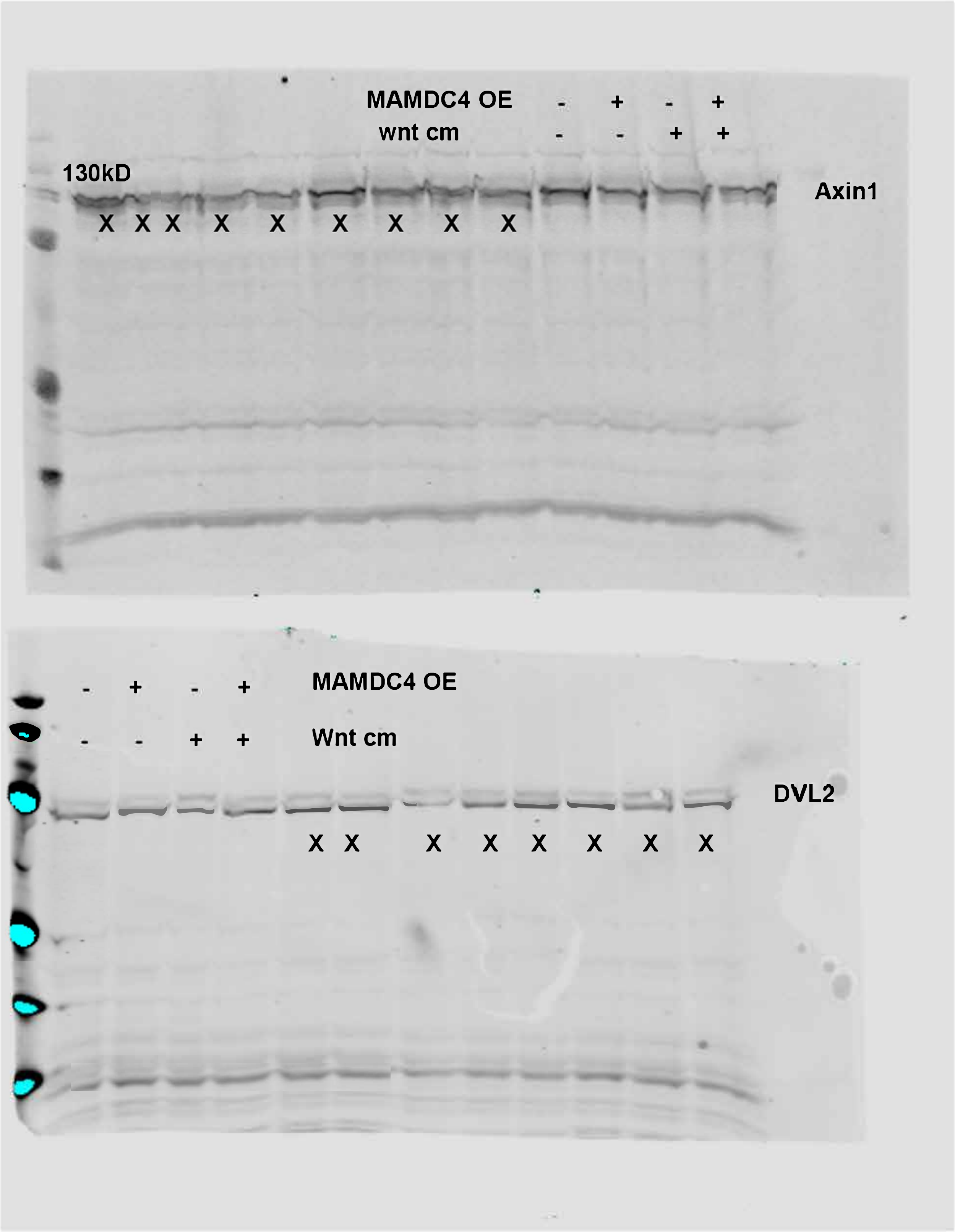
MAMDC4 OE - + - +
wnt cm - - + +
130kD
Axin1
X X X X X X X X X
- + - + MAMDC4 OE
- - + + Wnt cm
DVL2
X X X X X X X X

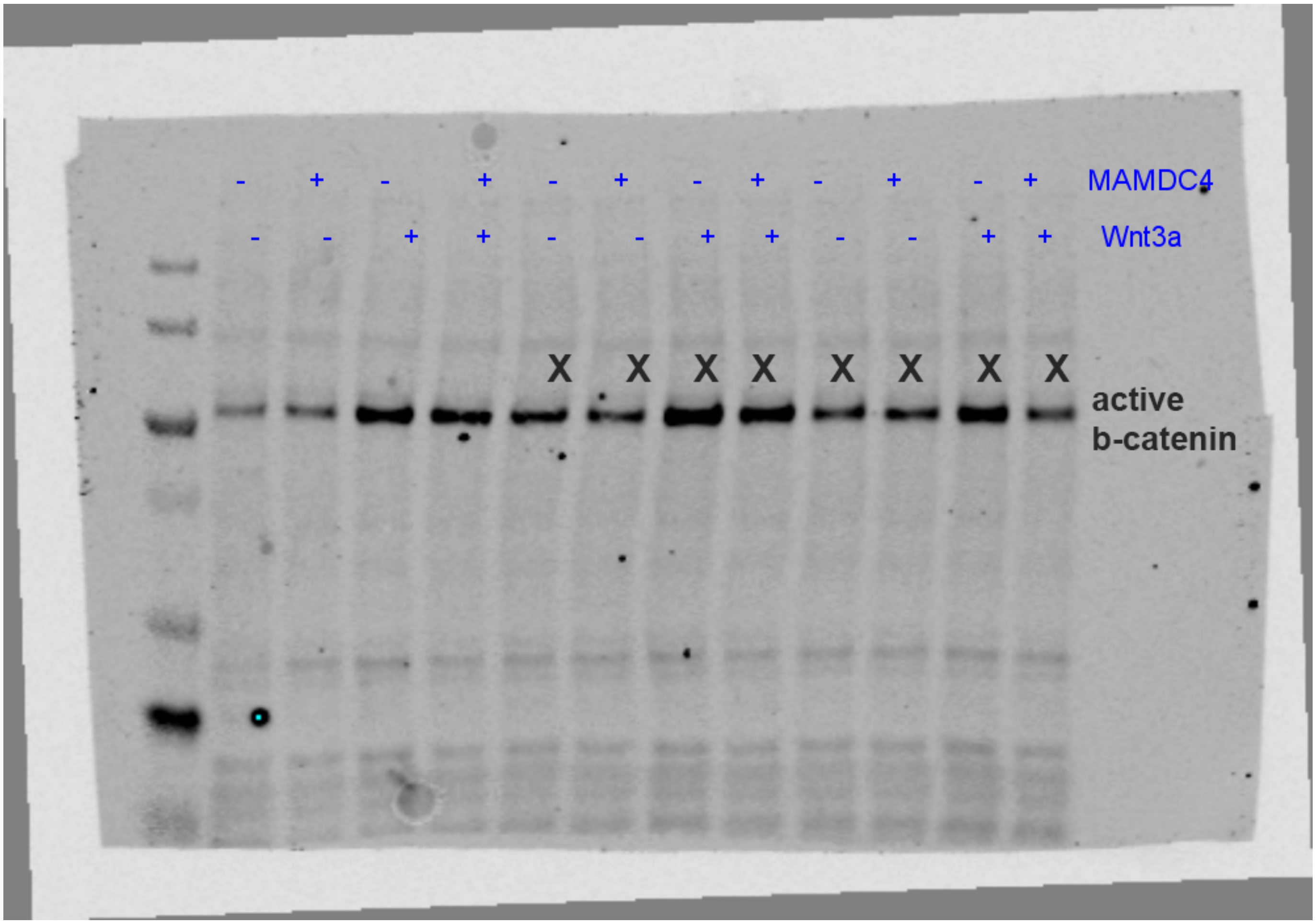

MAMDC4
Wnt3a
active
b-catenin

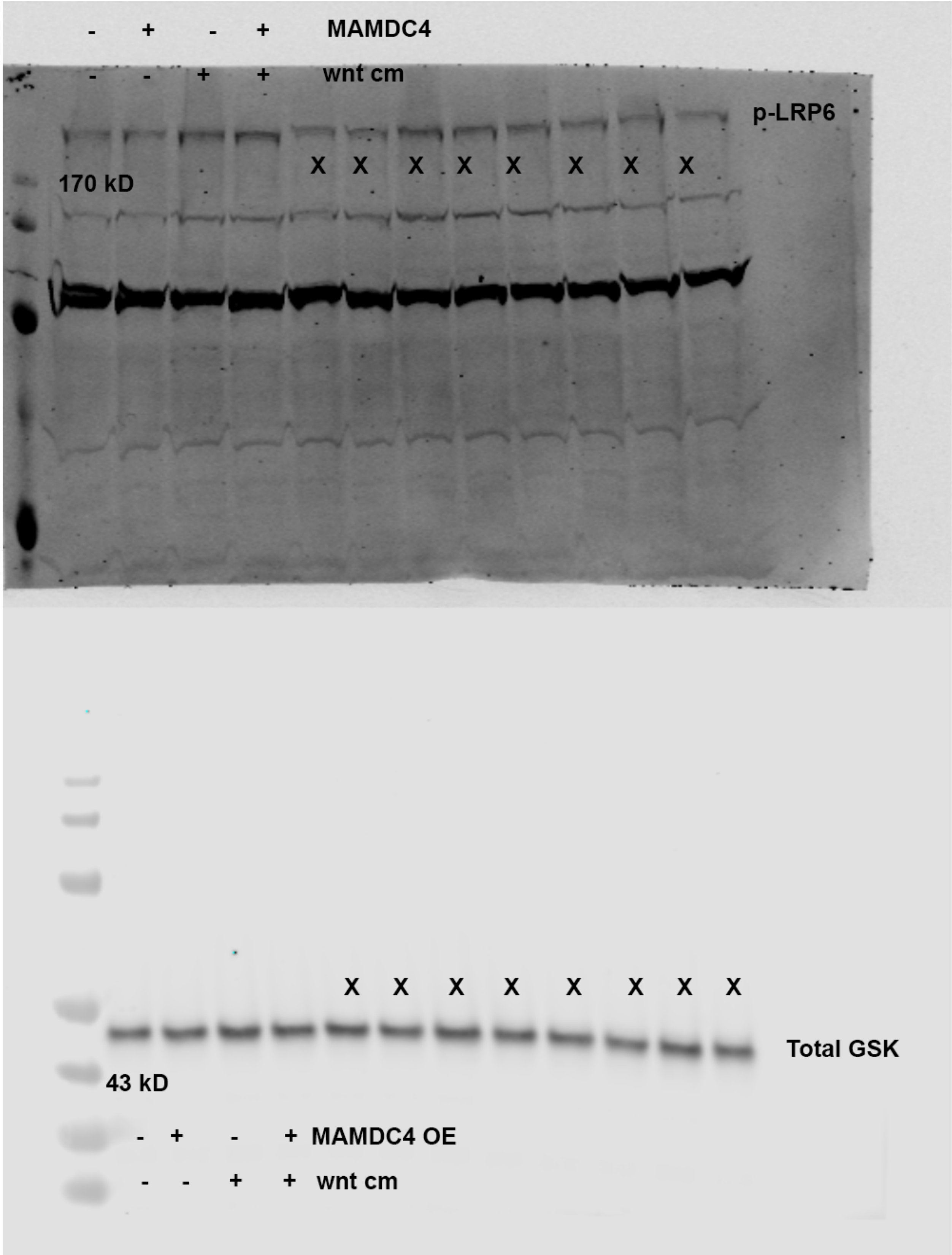
- + - + MAMDC4
- - + + wnt cm
p-LRP6
X X X X X X X X
170 kD
X X X X X X X X
Total GSK
43 kD
- + - + MAMDC4 OE
- - + + wnt cm

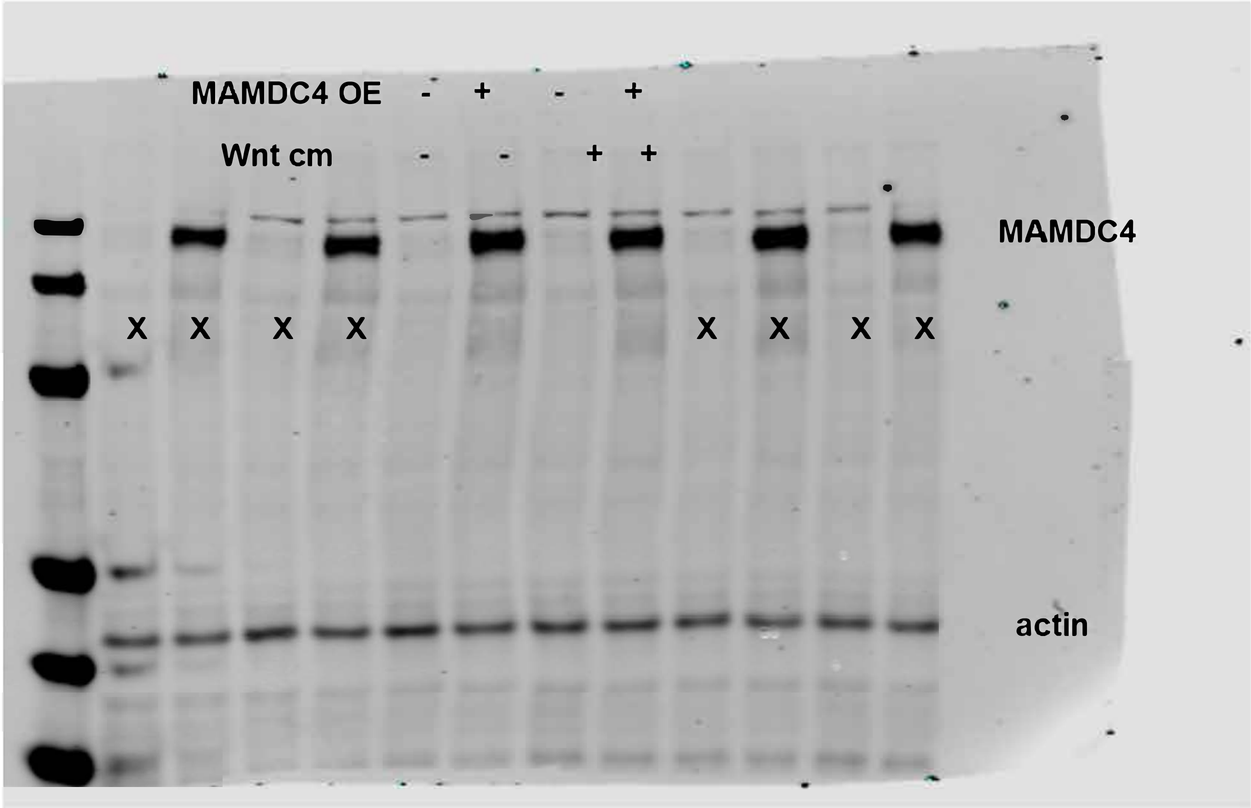
MAMDC4 OE - + - +
Wnt cm - - + +
MAMDC4
X X X X X X X X
actin

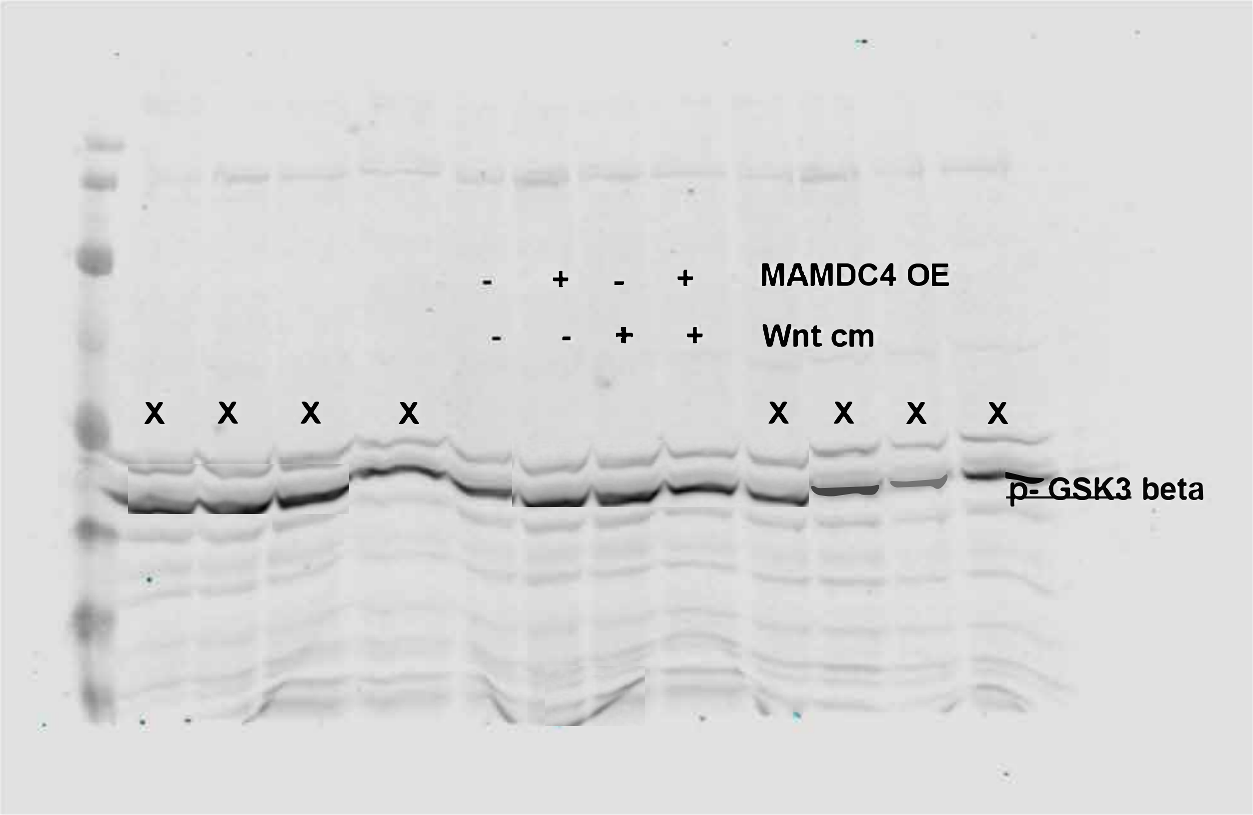
- + - + MAMDC4 OE
- - + + Wnt cm
X X X X X X X X
p- GSK3 beta

Figure 6 raw images

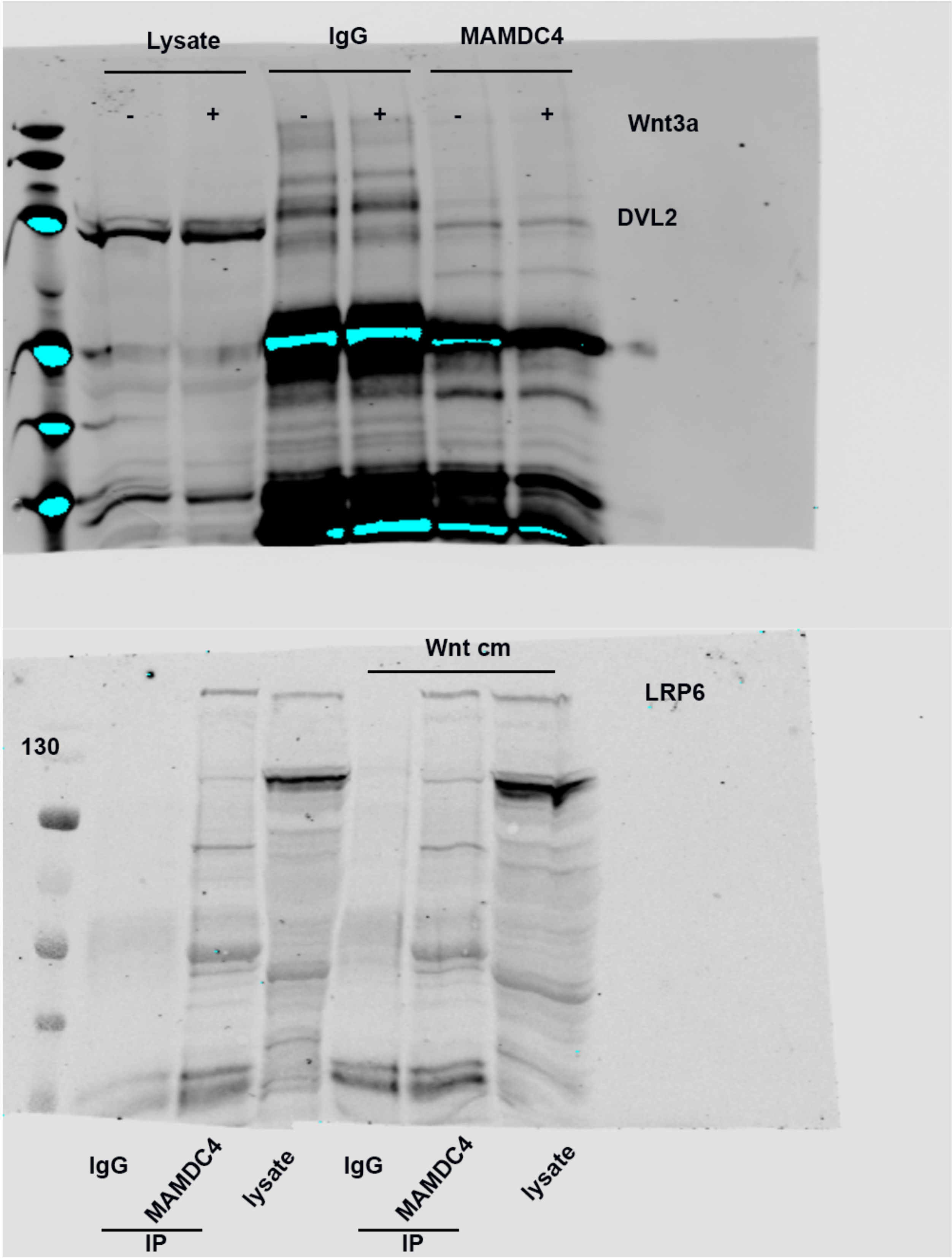

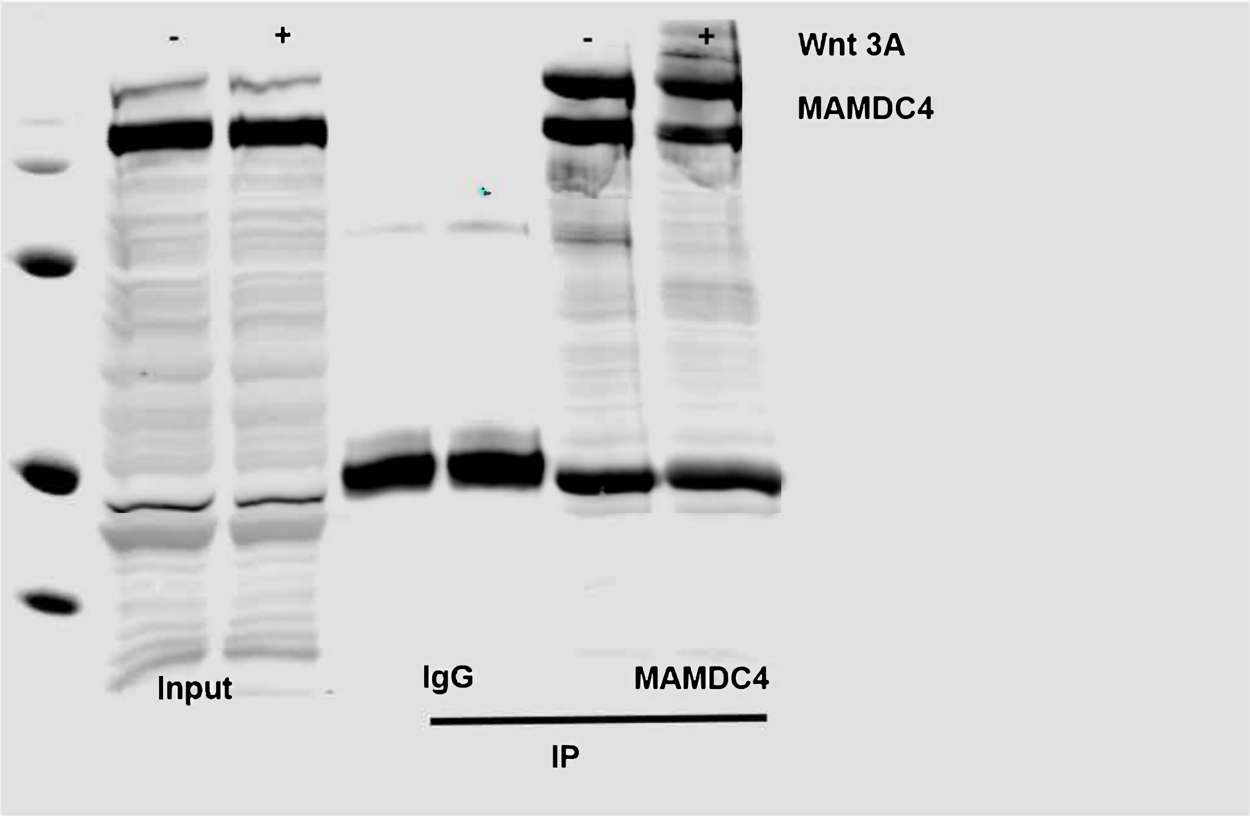

-
+
-
+
Wnt 3A
MAMDC4
Input
IgG
MAMDC4
IP

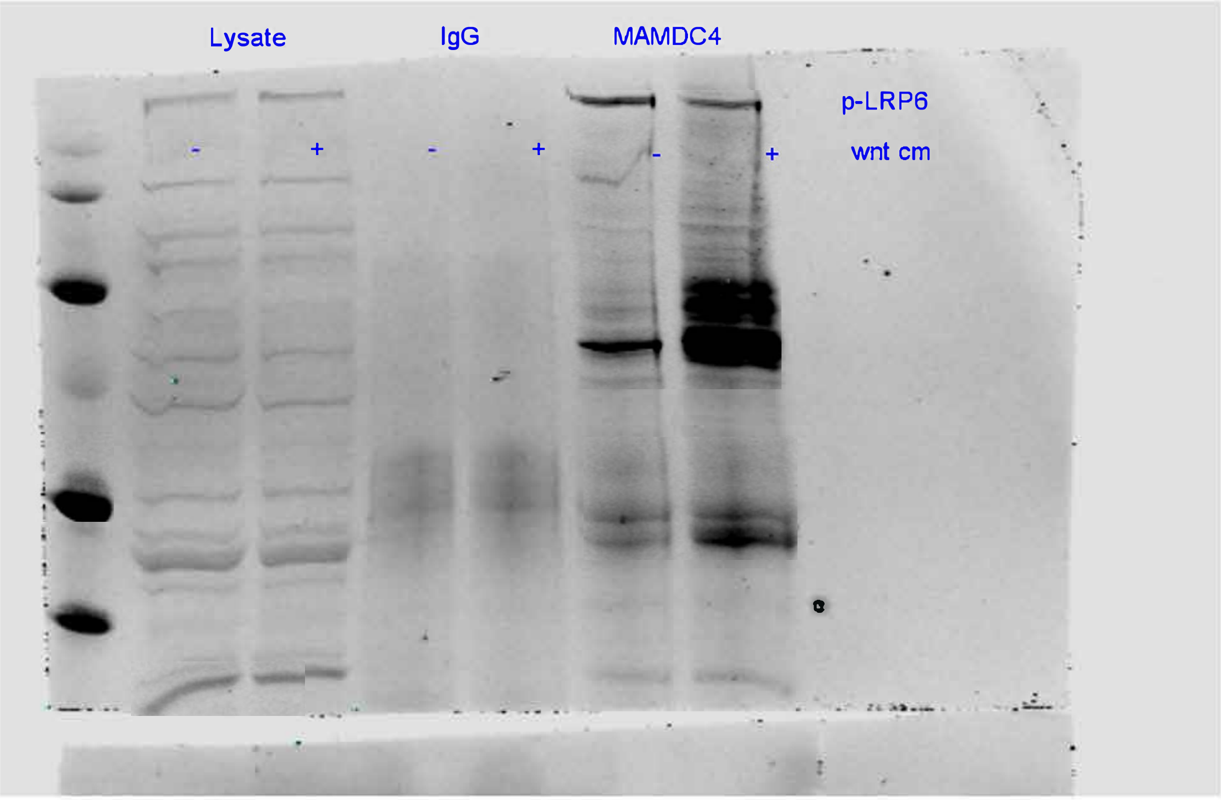

Lysate
IgG
MAMDC4
p-LRP6
-
+
-
+
-
+
wnt cm

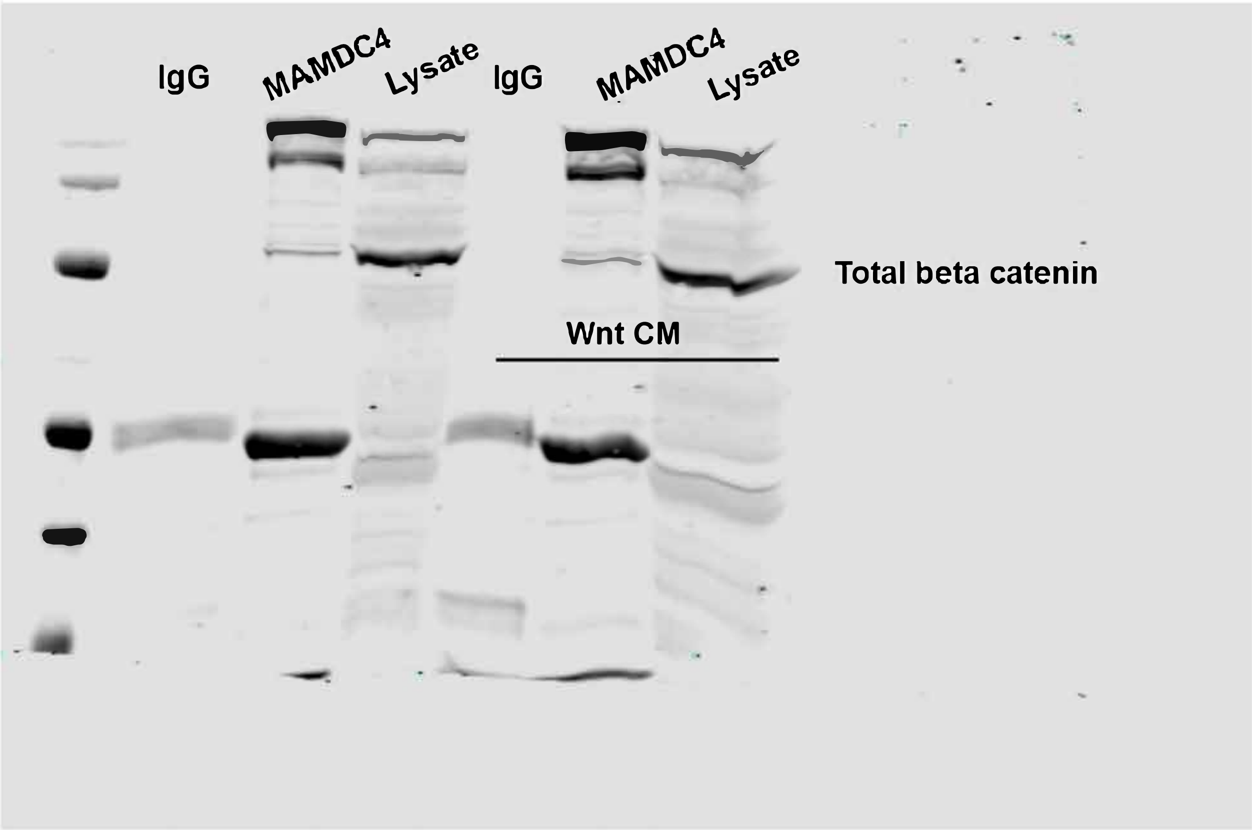
IgG
MAMDC4
Lysate
IgG
MAMDC4
Lysate
Total beta catenin
Wnt CM

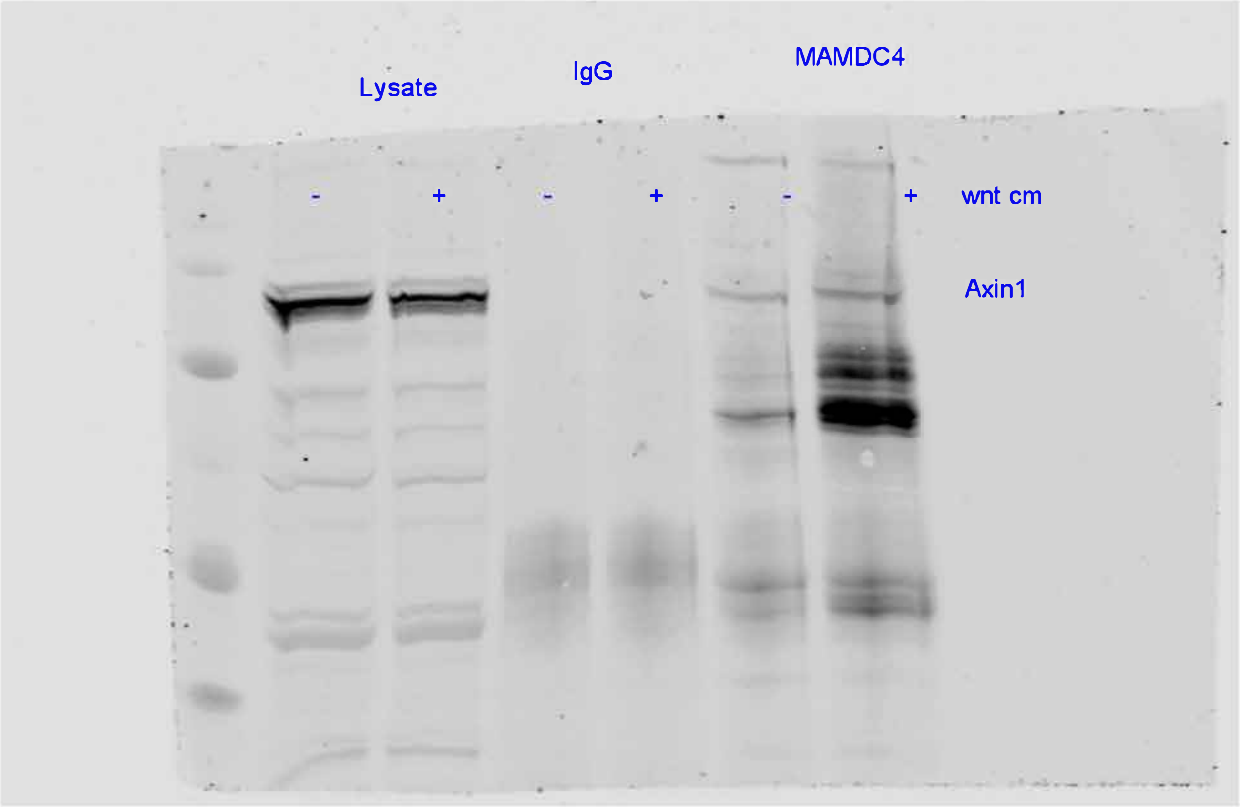
Lysate
IgG
MAMDC4
-
+
-
+
-
+
wnt cm
Axin1

Figure 7 raw images

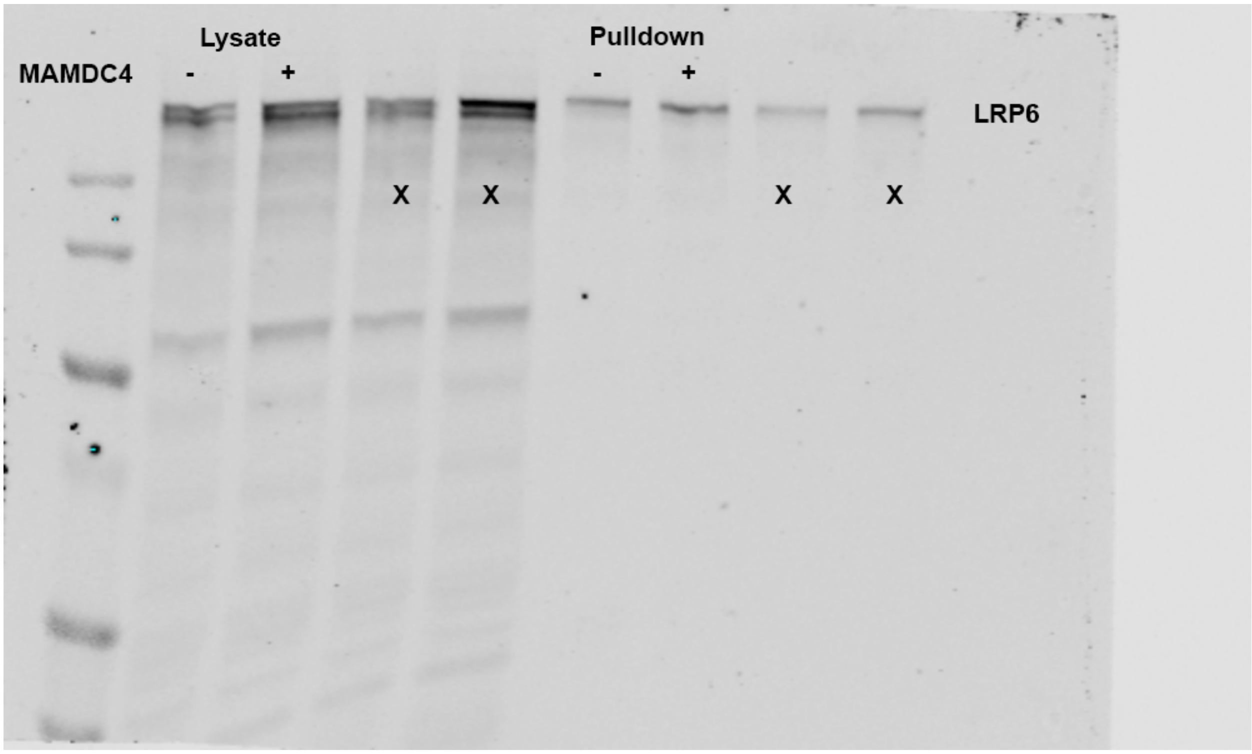

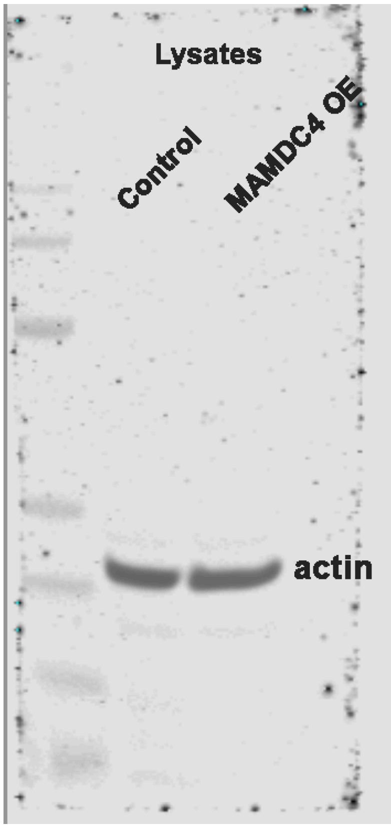

Figure 9 raw images

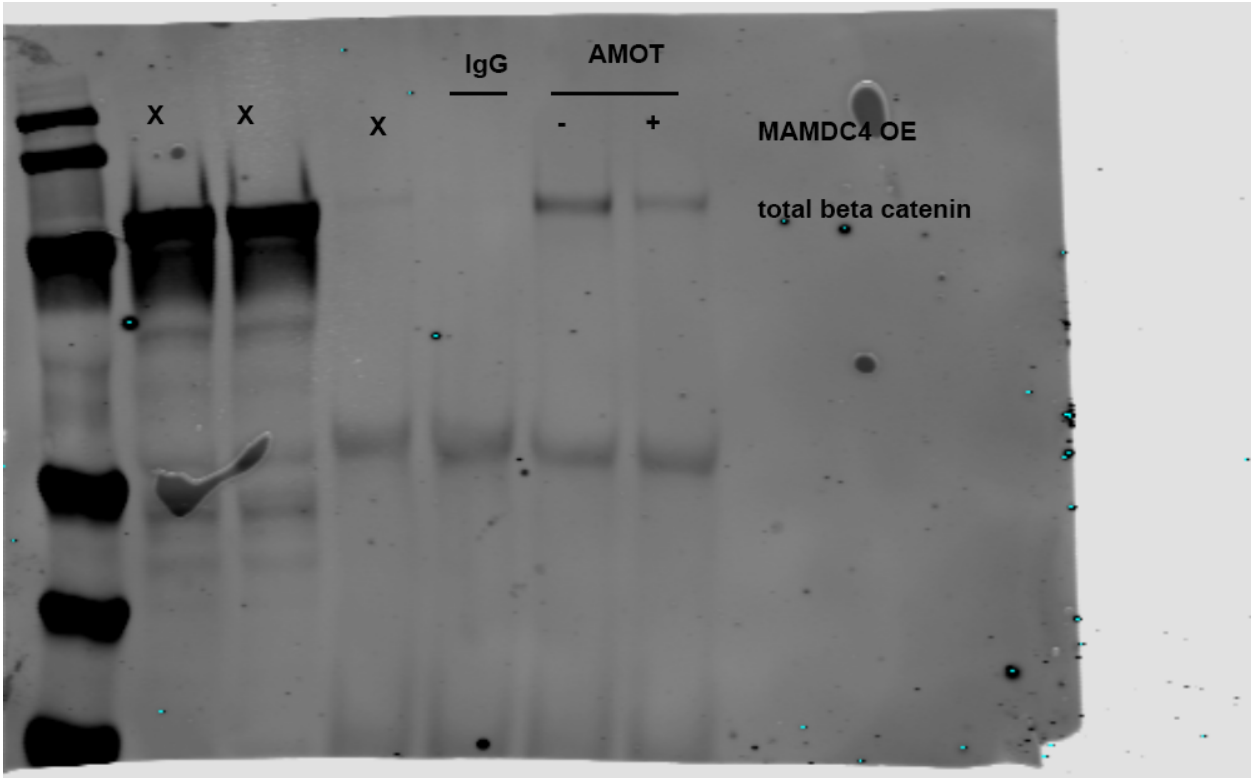

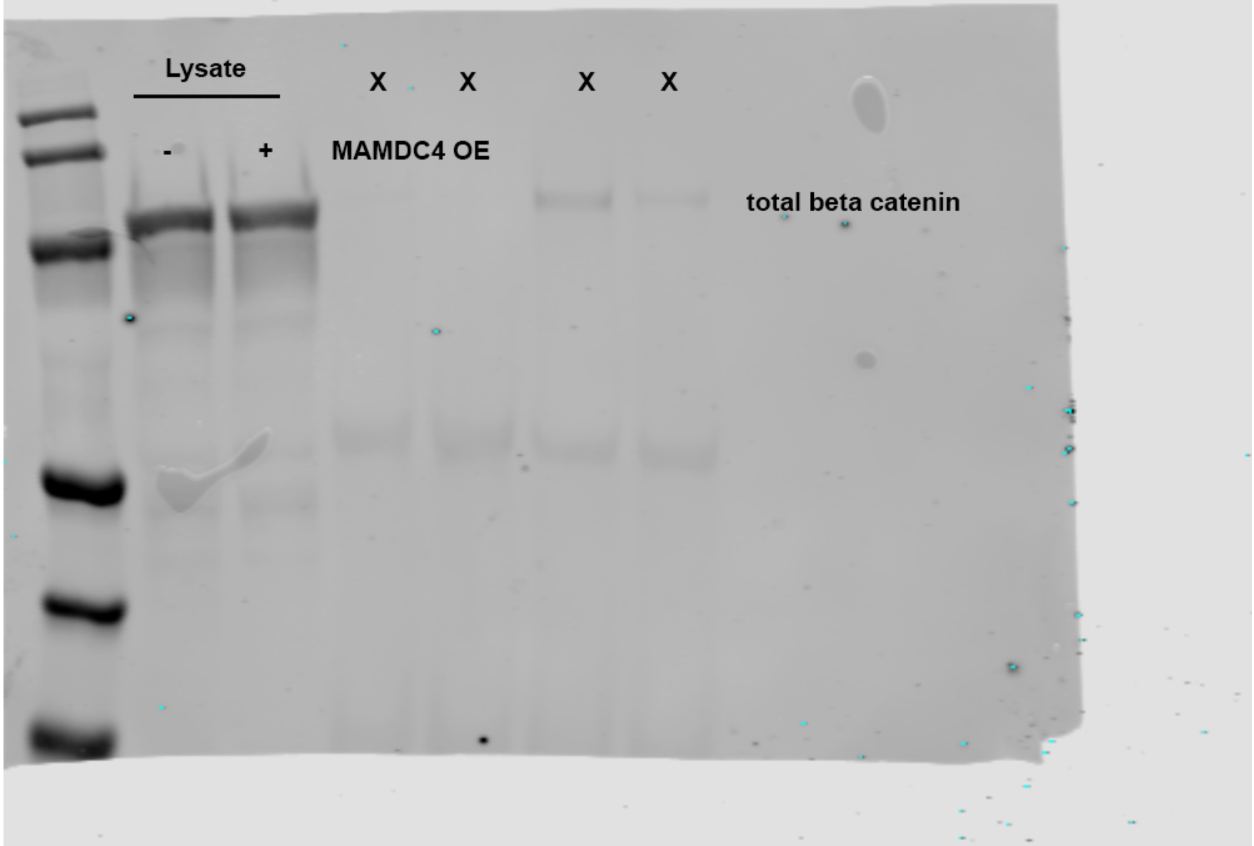

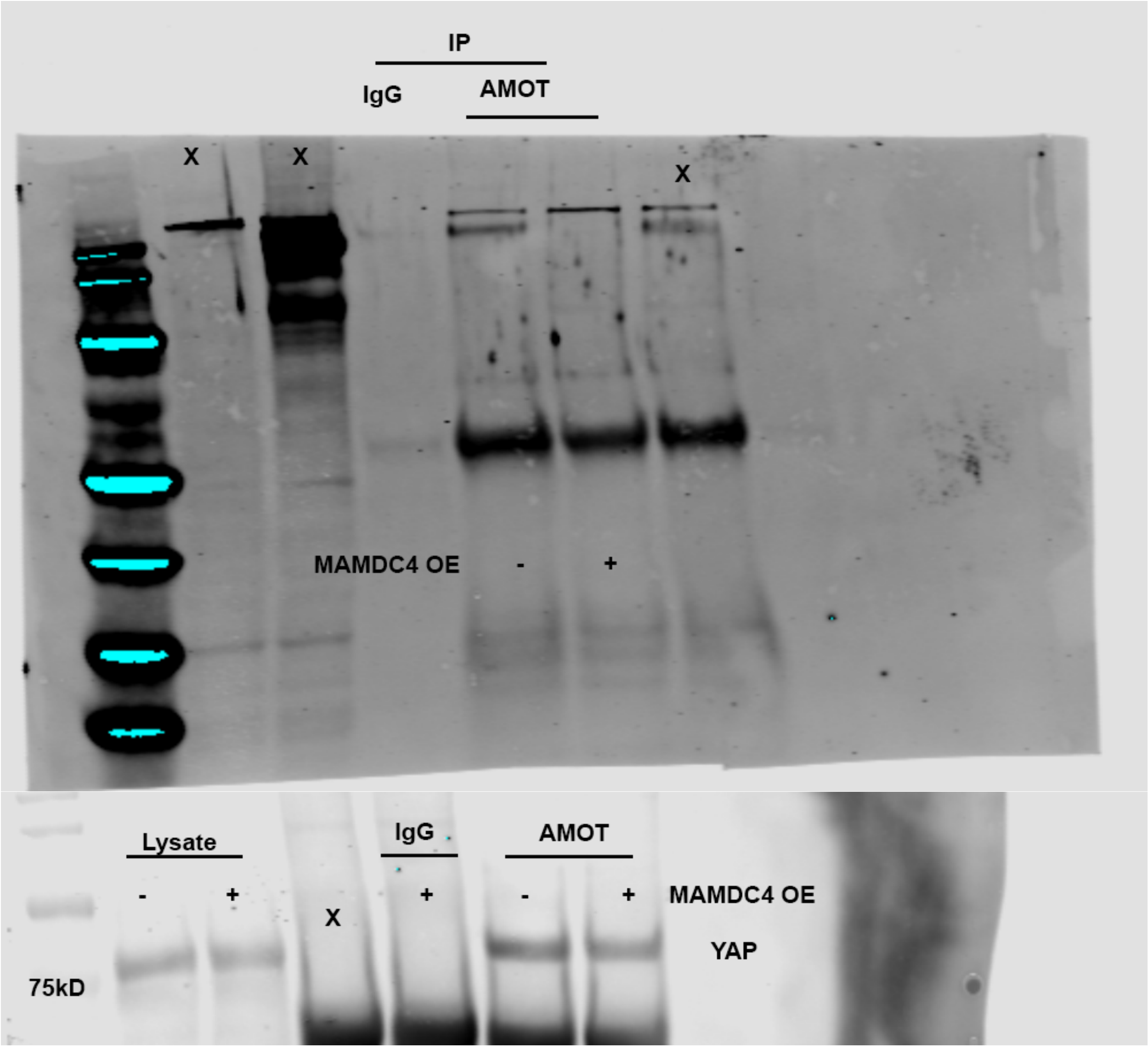
IP
IgG
AMOT
X
X
X
MAMDC4 OE
-
+
Lysate
IgG
AMOT
-
+
X
+
-
+
MAMDC4 OE
YAP
75kD

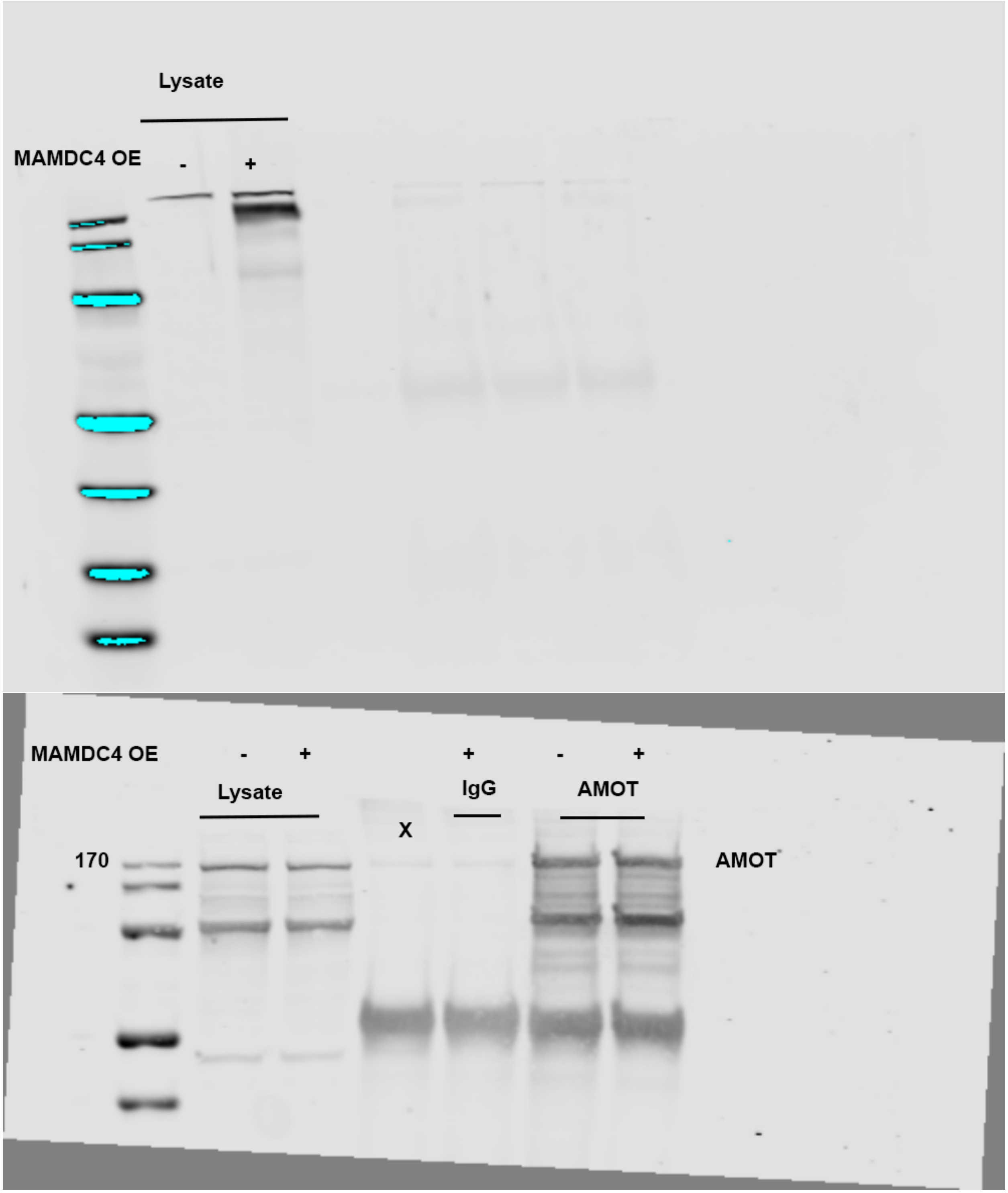
Lysate
MAMDC4 OE
-
+
MAMDC4 OE
-
+
+
-
+
Lysate
IgG
AMOT
X
170
AMOT

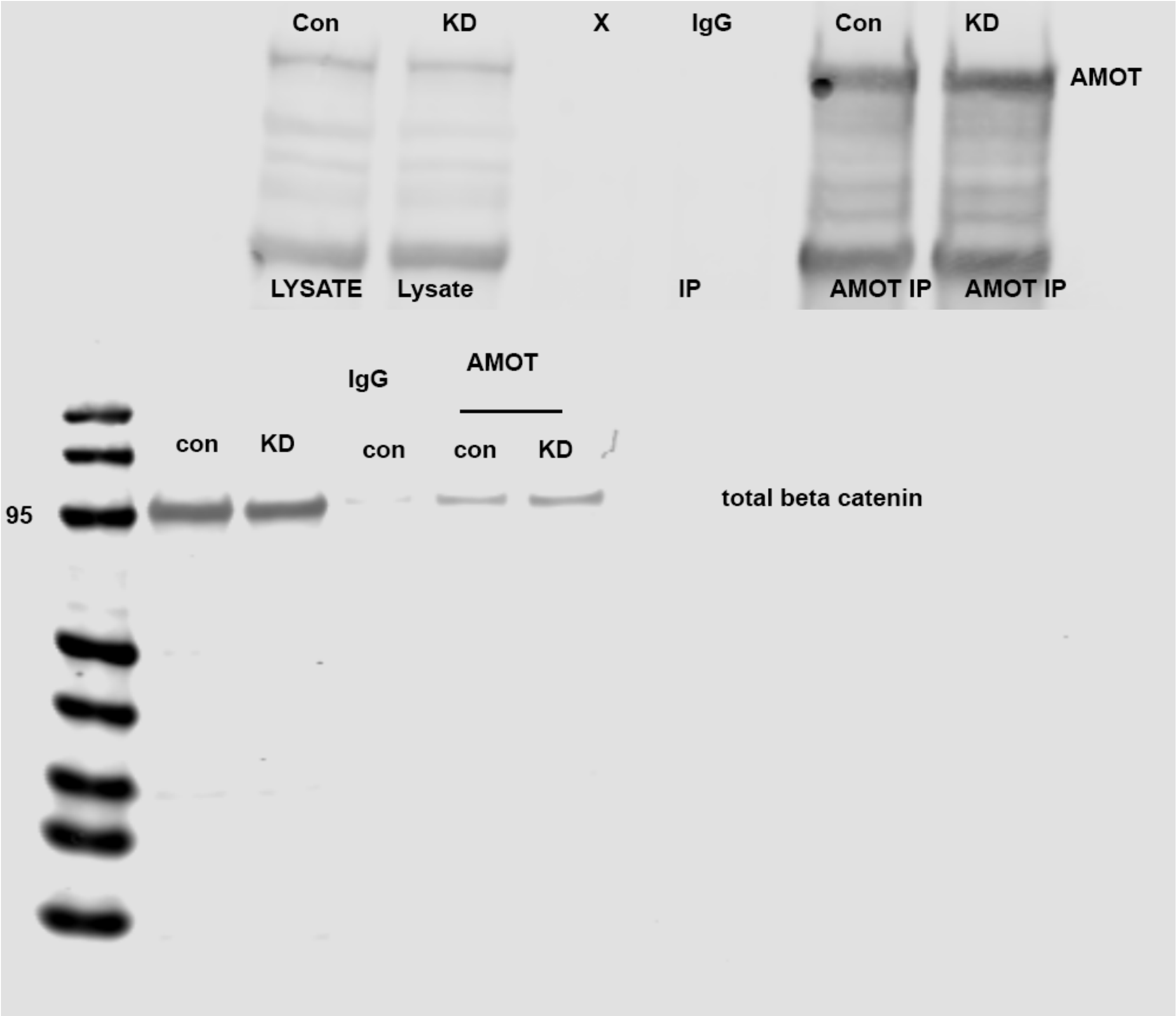
Con
KD
X
IgG
Con
KD
AMOT
LYSATE
Lysate
IP
AMOT IP
AMOT IP
IgG
AMOT
con
KD
con
con
KD
95
total beta catenin

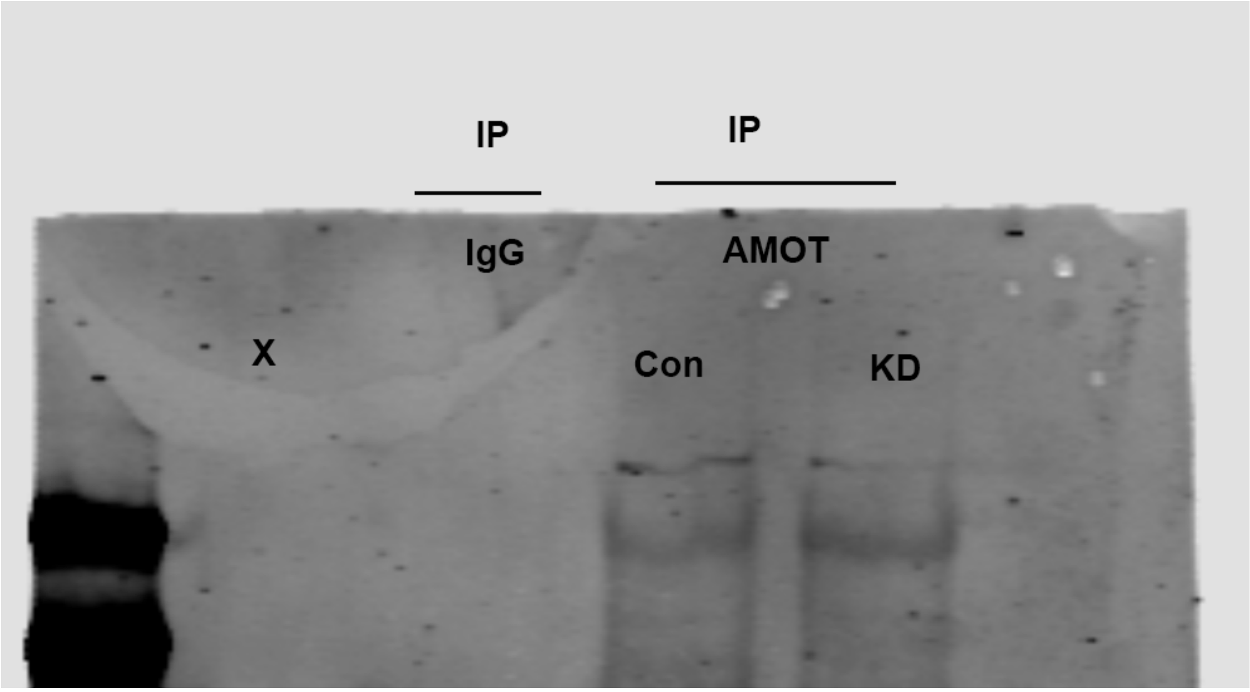
IP
IP
IgG
AMOT
X
Con
KD

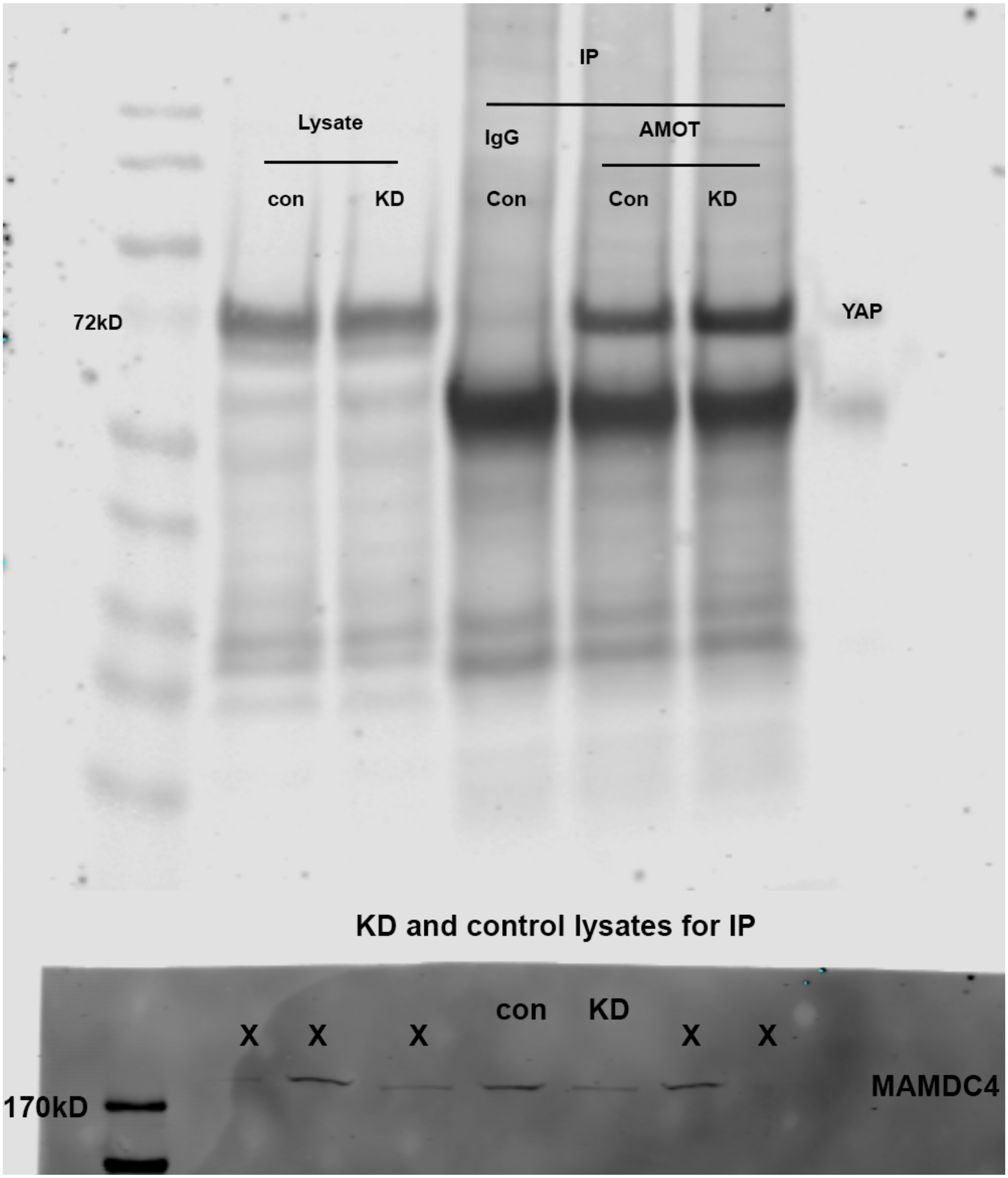

KD and control lysates for IP

Figure S2 raw images

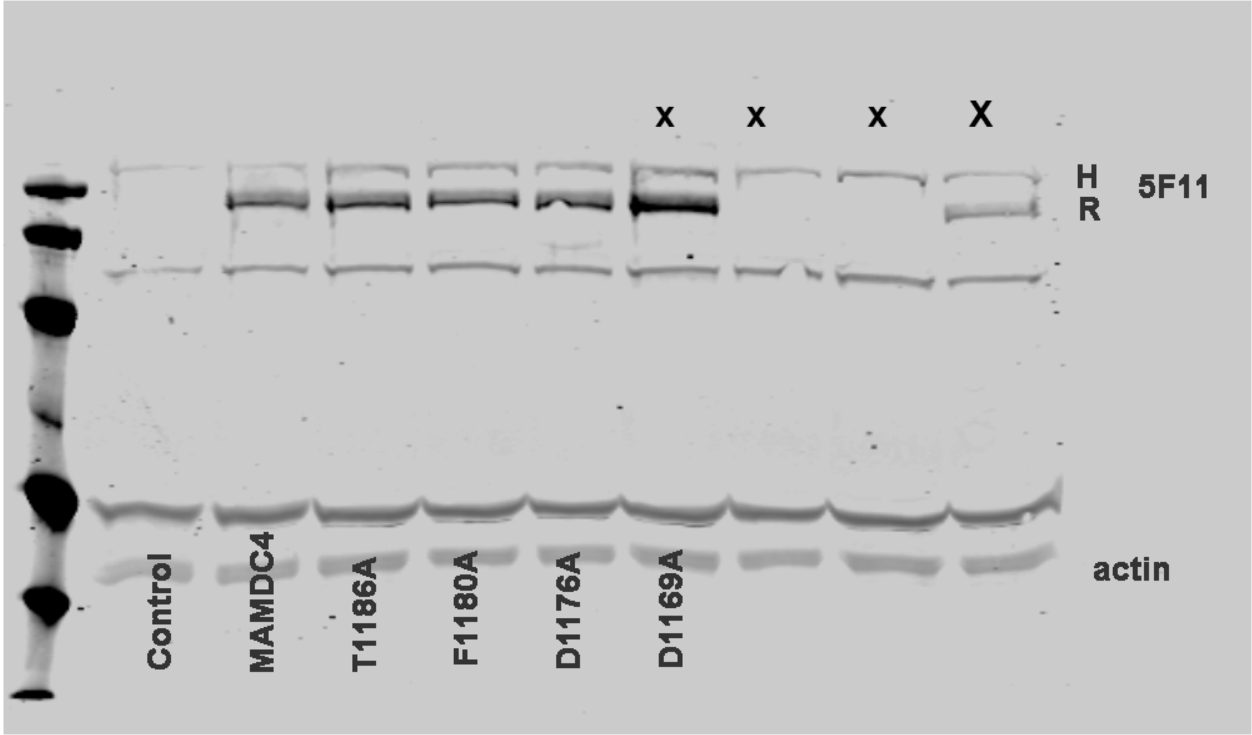

Figure S4 raw images

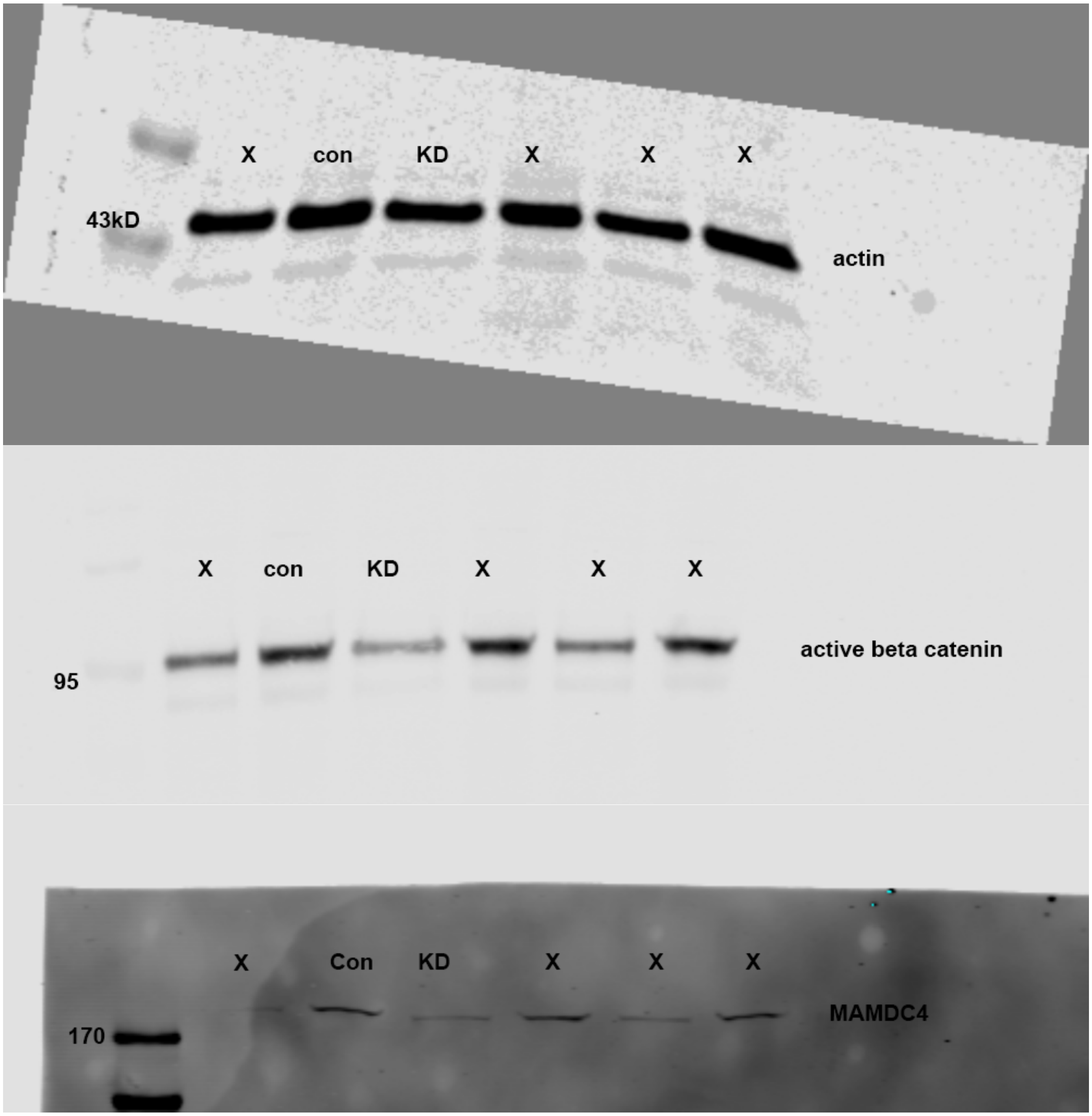

Supplement: S1 Raw images — (PDF) [file pone.0296003.s006.pdf]
